# Discovery of RC-752, a Novel Sigma-1 Receptor Antagonist with Antinociceptive Activity: A Promising Tool for Fighting Neuropathic Pain

**DOI:** 10.3390/ph16070962

**Published:** 2023-07-05

**Authors:** Giacomo Rossino, Annamaria Marra, Roberta Listro, Marco Peviani, Elena Poggio, Daniela Curti, Giorgia Pellavio, Umberto Laforenza, Giulio Dondio, Dirk Schepmann, Bernhard Wünsch, Martina Bedeschi, Noemi Marino, Anna Tesei, Hee-Jin Ha, Young-Ho Kim, Jihyae Ann, Jeewoo Lee, Pasquale Linciano, Marcello Di Giacomo, Daniela Rossi, Simona Collina

**Affiliations:** 1 Department of Drug Sciences, University of Pavia, 27100 Pavia, Italysimona.collina@unipv.it (S.C.); 2 Department of Biology and Biotechnology “L. Spallanzani”, University of Pavia, 27100 Pavia, Italy; 3 Human Physiology Unit, Department of Molecular Medicine, University of Pavia, 27100 Pavia, Italy; 4 Aphad SrL, Via della Resistenza, 65, 20090 Buccinasco, Italy; 5 Institut für Pharmazeutische und Medizinische Chemie, Westfälische Wilhelms-Universität Münster, Corrensstraße 48, D-48149 Münster, Germany; 6 BioScience Laboratory, IRCCS Istituto Romagnolo per lo Studio dei Tumori (IRST) “Dino Amadori”, 47014 Meldola, Italyanna.tesei@irst.emr.it (A.T.); 7 Medifron DBT, Seoul 08502, Republic of Korea; 8 Laboratory of Medicinal Chemistry, College of Pharmacy, Seoul National University, Seoul 08826, Republic of Korea; 9 JMackem Co. Ltd., Seoul 08826, Republic of Korea

**Keywords:** sigma 1 receptor antagonist, neuropathic pain, antinociceptive activity, zebrafish model

## Abstract

Neuropathic pain (NP) is a chronic condition resulting from damaged pain-signaling pathways. It is a debilitating disorder that affects up to 10% of the world’s population. Although opioid analgesics are effective in reducing pain, they present severe risks; so, there is a pressing need for non-opioid pain-relieving drugs. One potential alternative is represented by sigma-1 receptor (S1R) antagonists due to their promising analgesic effects. Here, we report the synthesis and biological evaluation of a series of S1R antagonists based on a 2-aryl-4-aminobutanol scaffold. After assessing affinity toward the S1R and selectivity over the sigma-2 receptor (S2R), we evaluated the agonist/antagonist profile of the compounds by investigating their effects on nerve growth factor-induced neurite outgrowth and aquaporin-mediated water permeability in the presence and absence of oxidative stress. (*R*/*S*)-**RC**-**752** emerged as the most interesting compound for S1R affinity (*K*_i_ S1R = 6.2 ± 0.9) and functional antagonist activity. Furthermore, it showed no cytotoxic effect in two normal human cell lines or in an in vivo zebrafish model and was stable after incubation in mouse plasma. (*R*/*S*)-**RC**-**752** was then evaluated in two animal models of NP: the formalin test and the spinal nerve ligation model. The results clearly demonstrated that compound (*R*/*S*)-**RC**-**752** effectively alleviated pain in both animal models, thus providing the proof of concept of its efficacy as an antinociceptive agent.

## 1. Introduction

Chronic pain represents one of the major therapeutic challenges nowadays and it can be divided into two categories: (i) nociceptive, which is more likely to be experienced and is due to injuries, cuts, and traumas and (ii) neuropathic pain (NP), which is caused by misfunction of the nerve pain signaling pathways [1,2]. This last type of chronic pain is a debilitating disorder that affects up to 10% of the world’s population and represents a significant burden for public health systems [3]. Neuropathic pain is often caused by diabetes, viral infections, chemotherapy, alcoholism, vitamin deficiency, and hypothyroidism [1,2,3,4]. Neuropathies correlated to viral infections have received great attention from the scientific community in recent times, as a consequence of the COVID-19 pandemic. In fact, the SARS-CoV-2 infection can cause neurological complications, including NP, that can last long after the acute phase of the disease [5,6,7,8]. Most common symptoms of NP include numbness of the limbs, stabbing pain, and burning pain, and they can all have a huge impact on a patient’s quality of life, compromising even simple tasks, which can eventually cause the insurgence of depression [3,9,10,11,12].

Traditional pain medications such as paracetamol, NSAIDs, and weak opioids are not effective in managing NP. The existing therapies for NP are based on repurposing drugs: the first-line treatments usually involve antidepressants such as duloxetine, amitriptyline, nortriptyline, and desipramine. Anticonvulsants such as pregabalin and gabapentin, or a combination of both, represent the most prescribed therapy for the treatment of different kinds of neuropathies (e.g., postherpetic neuralgia and peripheral diabetic neuropathy) [13,14]. Tramadol and tapentedol are second-line strategies, along with the application of capsaicin or lidocaine patches [15,16]. Stronger opioids are recommended as a last resort due to the several risks and side effects, such as respiratory depression, nausea, pharmacodynamic tolerance, and, importantly, the abuse potential [17]. The global non-opioid pain treatment market was valued at USD 13,200.6 million in 2020, and it is anticipated to grow at a CAGR of 16.8% between 2021 and 2030, with the COVID-19 pandemic expected to have both direct and indirect effects on this market [18]. 

However, meta-analyses revealed that only a small percentage of patients suffering from NP experience partial relief from the therapies mentioned [10]. Accordingly, continuous effort is needed in the search for new, safe, and more effective alternatives that are suitable for managing long-lasting and chronic NP conditions. The sigma-1 receptor (S1R) has emerged as one of the most promising targets currently under investigation and its implications for NP have been extensively investigated [19,20,21,22,23]. The S1R is found in various pain-modulating tissues and brain regions, including the dorsal spinal cord and root ganglion neurons. Studies have shown that the S1R may be involved in the modulation of diverse pain mechanisms, and its expression in the spinal cord is upregulated during the induction phase of neuropathic pain [24]. As a molecular chaperone, the S1R can influence the functionality of multiple ion channels, such as voltage-gated calcium channels and potassium channels, and the receptors involved in the transmission and amplification of nociceptive signals, and it can modulate the release of the neurotransmitters involved in pain signaling, such as glutamate and substance P [25,26,27]. As further evidence, NP is attenuated in S1R knockout (KO) mice [28]. S1R antagonists have been shown to exert antinociceptive effects in preclinical models of the inflammatory pain, ischemic pain, and neuropathic pain induced by nerve trauma or chemical injury [28,29,30,31,32]. Furthermore, S1R antagonists have the potential to counteract chemotherapy-induced peripheral neurotoxicity (CIPN), a pathologic condition that frequently occurs in cancer patients [33,34,35].

Over the years, several S1R antagonists have been proposed, but their poor selectivity toward the S2R does not make them suitable for therapeutical applications [36]. Indeed, S1R antagonists, which are also active on the S2R (pan-sigma modulators), can exert an anti-proliferative and pro-apoptotic activity which is potentially useful against various tumors [37,38,39]. This behavior may entail toxicity and safety issues that might prevent the use of pan-SR modulators for therapeutic applications other than those for cancer. Thus, for chronic pain treatment, selective S1R antagonists are demanded.

**Figure 1:**
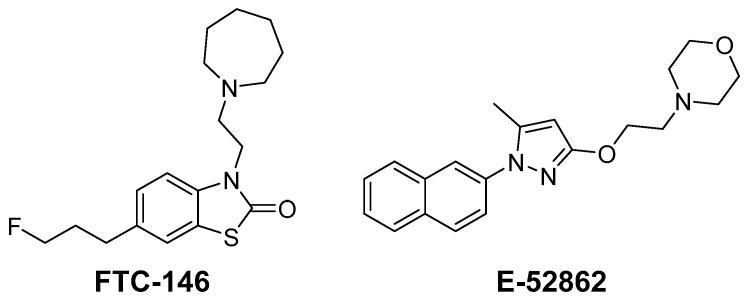
Sigma-1 receptor (S1R) antagonists with potential therapeutical activity for pain diagnosis and management.

The S1R antagonists [^18^F]FTC-146 [40] and E-52862 [41] (Figure 1) have undergone Phase 1 and Phase 2 clinical trials, respectively [42,43]. More specifically, the S1R radiotracer [^18^F]FTC-146 was successfully used in a positron emission tomography/magnetic resonance imaging (PET/MRI) application to identify the source of pain in patients suffering from complex regional pain syndrome (CRPS) and chronic sciatica by enabling the visualization of nerve damage in neuropathic conditions [40,44,45]. The selective S1R antagonist E-52862 was instead proved to be effective in ameliorating oxaliplatin-induced peripheral neuropathy in patients treated for colorectal cancer [46,47] and in the treatment of acute post-operative pain, post-herpetic neuralgia (PHN), and diabetic neuropathy [48,49].

Within this context and as a part of our ongoing research in the field of SR modulators, our goal is the discovery of novel S1R antagonists endowed with high therapeutic potential for the treatment of NP. To achieve this, a new library of S1R modulators was prepared, and their biological profile was investigated, following a funnel workflow to select the best candidate for the in vivo proof of concept. SR binding affinity and selectivity, as well as the agonist/antagonist functional profile of the S1R, were evaluated first. Then, toxicity and metabolic stability were assessed to prioritize the molecules on the basis of their in vitro developability profile, before the investigation in the animal models of NP (i.e., with the formalin-induced licking paw test and Chung’s model). 

## 2. Results and Discussion

### 2.1. Design and Synthesis

**Figure 2:**
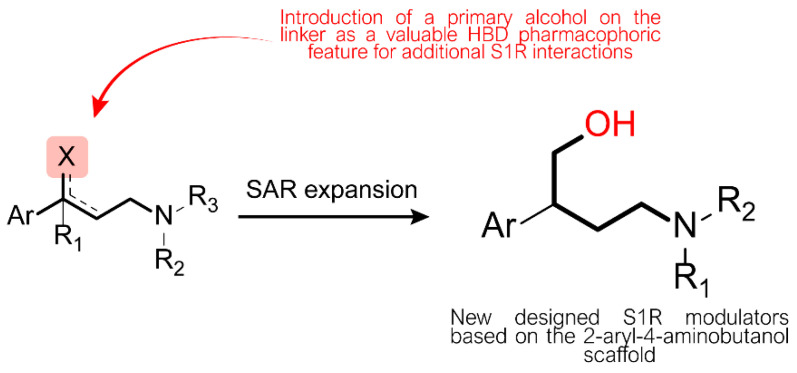
Design of new potential S1R modulators with 2-aryl-4-aminobutanol scaffold.

A wide number of SR modulators were designed by our group by combining different aromatic portions (i.e., naphth-2-yl, naphth-1-yl, biphen-4-yl, 6-hydroxy-, and 6-methoxy-naphth-2-yl); aminic moieties (i.e., N,N-dimethylamine, N-benzyl-N-methylamine, piperazine, morpholine, 4-benzylpiperidine/piperazine); and a linker spacer between the aromatic portion and the aminic moiety (i.e., arylalkenyl-, arylalkylamines, arylalkylaminoalcohols and arylalkylketone-) [50,51,52,53]. In the present paper, we expand the SAR around the 3-aryl-butanamine scaffold by introducing a primary alcohol on the linker as a valuable HBD pharmacophoric feature for additional S1R interactions (Figure 2) [54]. 

**Scheme 1:**
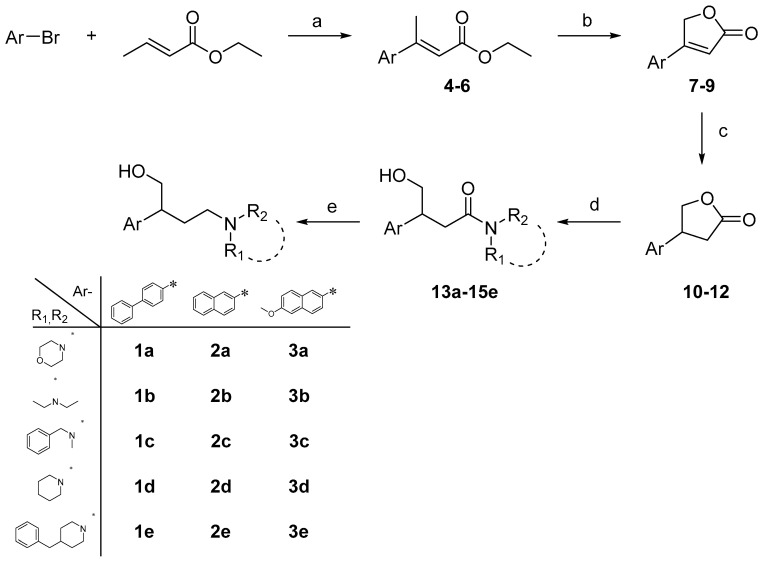
Reagents and conditions: (a) 4-phenyl-bromo-benzene (for **4**, **7**, **10**, and **13**), 2-bromo-naphtalene (for **5**, **8**, **11**, and **14**), or 5-methoxy-bromo-naphtalene (for **6**, **9**, **12**, and **15**) (1 equiv.), ethyl crotonate (1.5 equiv.), AcOEt (2 equiv.), Bu_4_NCl (2 equiv.), and Pd(OAc)_2_ (0.05 equiv.), anh. DMF, N_2_, 105 °C, 3–5 h, 51–59% yield; (b) **4**–**6** (1 equiv.), SeO_2_ (1.3 equiv.), CH_3_CN, mw T = 100 °C, W = 180, PSI = 200, run time = 15 min each cycle; (c) **7**–**9** (1 equiv.) HCO_2_NH_4_ (5 equiv.), Pd/C (35% wt), AcOEt/t-BuOH, mw T = 100 °C, W = 180, PSI = 200, run time = 5 min, 31–53% yield over two steps; (d) morpholine (for **13a**–**15a**), diethylamine (for **13b**–**15b**), N-benzyl-N-methylamine (for **13c**–**15c**), piperidine (for **13d**–**15d**), 4-benzylpiperidine (for **13e**–**15e**) (2.5 equiv.), AlCl_3_ (1.3 equiv.), **10**–**12** (1 equiv.), 1,2-dichloroetane, 0° C to r.t., 3–5 h, 40–99% yield; e) **13a**–**15e** (1 equiv.), LiAlH_4_ (2 equiv.), anh. THF, −15 °C to r.t., 2–4 h, 13–67% yield.

According to our previous SAR analysis, biphen-4-yl, naphth-2-yl, and 6-methoxy-naphth-2-yl were chosen as aromatic moieties for the hydrophobic interaction with the primary binding region of the S1R. Furthermore, it was known that the bulky 4-benzylpiperidine moiety significatively increases the affinity toward the S2R. Thus, to improve selectivity, only small aliphatic amines (i.e., morpholine, piperidine, diethylamino, and N-methyl-N-benzylamine) were considered (**1a**–**d**, **2a**–**d**, and **3a**–**d**, Scheme 1). The corresponding benzylpiperidine derivatives (**1e**, **2e**, and **3e**, Scheme 1) were prepared as well to support our previous observation. All the designed compounds passed the check for pan-assay interference compounds (PAINS) as evaluated with the in silico tool FAFDrugs4 [55]. 

Compounds **1a**–**3e** were prepared according to the synthetic pathway reported in Scheme 1. The first step consisted of a Heck reaction, which was exploited to introduce the first point of diversification: three different aryl bromides were reacted with methyl crotonate to afford the α, β-unsaturated esters **4**–**6**. The reaction was performed under phosphine-free conditions, employing tetraethyl ammonium chloride (TEAC) according to the procedures already described [56]. Consistently with the literature data, the major product obtained was the (*E*)-isomer, which is thermodynamically more stable and has the correct geometry for the formation of the lactone intermediates **7**–**9** [57]. These were obtained after an allylic oxidation promoted by SeO_2_ under microwave (mw) irradiation and the subsequent in situ cyclization. Afterwards, the conjugated double bond was reduced with ammonium formate as a hydrogen source employing Pd/C. The saturated lactones **10**–**12** were then subjected to aminolysis with the amines **a**–**d**. This reaction opened the five-membered cycle, generating the alcohol group characteristic of the whole series and introducing the second point of diversification. The amide group was finally reduced with LiAlH_4_ to afford the target compounds **1a**–**3e**. These were converted to their hydrochloride salts and obtained in an amount and a purity suitable for biological investigations.

### 2.2. Binding Affinity

(*R*/*S*)-**1a**–**3e** were first evaluated for their binding affinity toward the S1R and S2R in a radioligand displacement assay. Briefly, the assay was performed using homogenized guinea pig brain in the presence of [^3^H]-(+)-pentazocine as a selective S1R radioligand. Nonspecific binding was determined using non-radiolabeled (+)-pentazocine and haloperidol in excess. All the binding experiments were carried out in duplicate. Three independent experiments were performed for each compound to determine S1R affinity, whereas only the compounds displaying high affinity (*K*_i_ < 100 nM) toward the S2R were tested in triplicate. Haloperidol was used as a control compound in both assays. The resulting *K*_i_ values are reported in Table 1.

**Figure 3:**
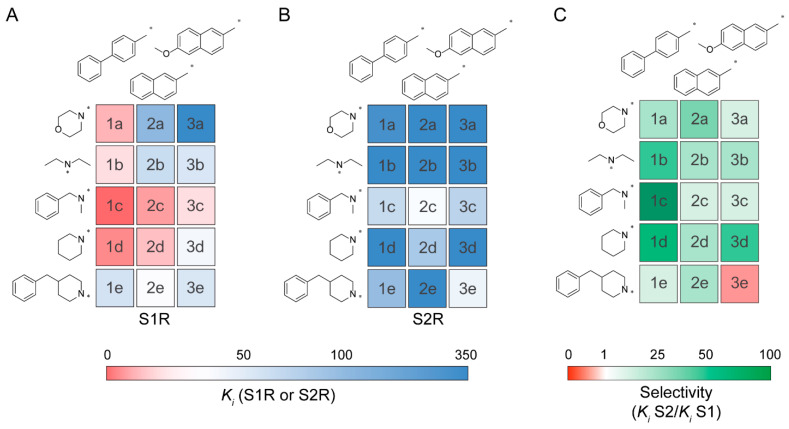
Structure–activity relationship (SAR) depiction for compounds **1a**–**3e** on S1R (**A**) and S2R (**B**) affinity and selectivity (**C**) by R-group analysis.

The consistent substitution pattern around the primary 2-aryl-4-aminobutanol scaffold facilitates the application of the R-group analysis for the SAR depiction and identification of key functionalities that positively or negatively affect affinity and selectivity (Figure 3).

Generally, both the aryl and amine groups play a key role in determining affinity and selectivity toward the S1R and S2R. Regarding S1R affinity, the most important issues to be highlighted are the following: (i) the biphenyl derivatives (**1a**–**d**) possessed higher affinity compared to the naphthyl ones (**2a**–**d**); (ii) within the whole **1**–**3** series, the S1R affinity seemed to be strictly correlated with the hydrophobicity of the amine moiety (the higher the lipophilicity, the higher the affinity, with the exception of the benzylpiperidine derivatives **1**–**3e**); and (iii) **1c** and **1d** emerged as the most active compounds of the whole library. The lipophilicity of the amine group also played a key role in determining the S2R/S1R selectivity, especially for the compounds belonging to series **1**. In fact, **1c** and **1d**, endowed with the highest S1R affinity, also resulted in the most selective ones. Conversely, within series **2** and **3** the selectivity was more variable and depended on the combination of the aryl and amine portions.

A brief separate comment is necessary for the 4-benzylpiperidine derivatives (**1e**, **2e**, and **3e**). As expected, 4-benzylpiperidine, while providing good affinity for the S1R also contributed significantly to reinforcing the binding for the S2R, leading to a pan-sigma modulator (**1e**) or a compound with a preference for the S2R (**3e**), thus confirming our previous experimental evidence [58]. 

### 2.3. Functional Profiling

According to their binding profile at the S1R and their selectivity over the S2R (*K*_i_ S1R < 15 nM and *K*_i_ S2R/S1R > 10), the compounds (*R*/*S*)-**1a**, (*R*/*S*)-**1c**, (*R*/*S*)-**1d**, and (*R*/*S*)-**2d** were chosen in order to study their activity at these receptors. The functional S1R profile was assessed in two cellular systems by evaluating the effect on the neurite outgrowth promoted by nerve growth factor (NGF) in PC12 cells and the water permeability mediated by aquaporins (AQPs) in HeLa cells, under normal and oxidative stress conditions. 

**Figure 4:**
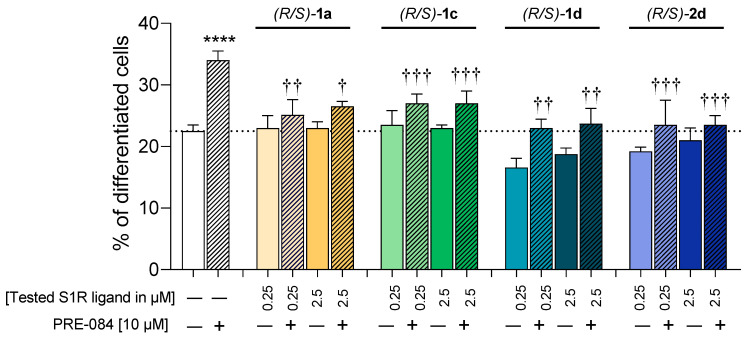
Effect of S1R ligands assessed with the PC12 differentiation assay. Two concentrations (0.25 µM and 2.5 µM) for each ligand were tested either alone or co-incubated with 10 µM PRE 084. Histograms represent the mean ± SEM of the percentage of differentiated cells from at least three different experiments. The dotted line represents the % of differentiated cells in untreated samples. Two-way ANOVA followed by Dunnett’s post hoc test: ****, *p* < 0.0001 versus Ctrl. †, *p* <0.05; ††, *p* < 0.01; †††, *p* < 0.001 vs. PRE-084.

In the PC12 neurite outgrowth assay, the S1R agonists enhanced the neuronal differentiation promoted by NGF, leading to an increase in neurite outgrowth and length with respect to NGF alone, whereas the S1R antagonists had no effect [59,60,61]. The compounds (*R*/*S*)-**1a**, (*R*/*S*)-**1c**, (*R*/*S*)-**1d**, and (*R*/*S*)-**2d** were assessed at 0.25 μM and 2.5 μM, alone or in co-administration with 10 μM PRE-084, a well-known S1R agonist [62]. NGF alone induced the differentiation in 22 ± 2% of the PC12 cells at 2.5 nM, whereas PRE-084 was able to promote the neurite outgrowth in the 36 ± 4% of the PC12 cells at 10 μM compared to the control. As reported in Figure 4, compounds (*R*/*S*)-**1a**, (*R*/*S*)-**1c**, (*R*/*S*)-**1d**, and (*R*/*S*)-**2d** did not significantly enhance the NGF-induced neurite outgrowth at either of the tested concentrations. Interestingly, the co-administration of compounds (*R*/*S*)-**1a**, (*R*/*S*)-**1c**, (*R*/*S*)-**1d**, and (*R*/*S*)-**2d** with PRE-084 significantly reduced the percentage of differentiated PC12 cells, indicating their ability to counteract the activity of the S1R agonist PRE-084 and thus suggesting that these four compounds have an S1R antagonist profile. Among the tested compounds, (*R*/*S*)-**1d** resulted in being the most effective in reducing the NGF-induced cell differentiation promoted by PRE-084.

**Figure 5:**
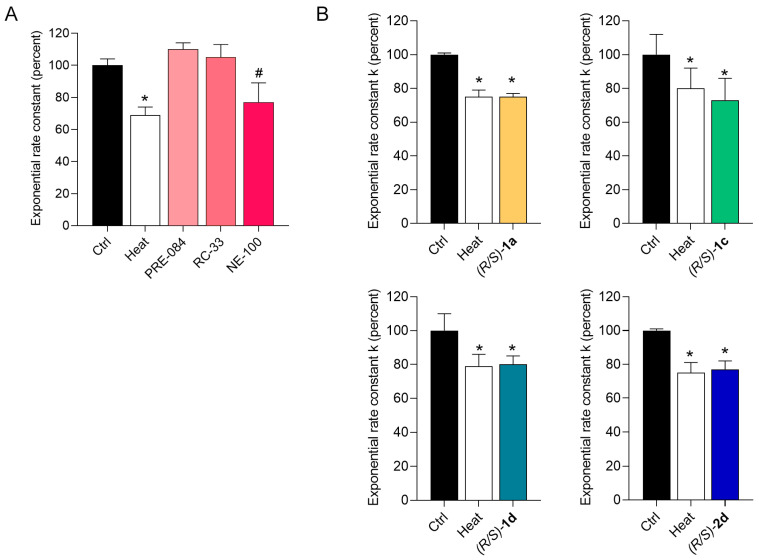
(**A**) Effect of known S1R agonists and antagonists on water permeability. (**B**) Effect of test compounds (*R*/*S*)-**1a**, (*R*/*S*)-**1c**, (*R*/*S*)-**1d**, and (*R*/*S*)-**2d** on water permeability. Cells were exposed to 150 mOsm osmotic gradients in three different conditions: untreated cells (Ctr), heat-stressed cells (Heat), and heat-stressed cells pre-treated with the S1R agonists and antagonists or test compounds (*R*/*S*)-**1a**, (*R*/*S*)-**1c**, (*R*/*S*)-**1d**, and (*R*/*S*)-**2d** at 20 μM final concentration. Bars represent the osmotic water permeability of HeLa cells expressed as percent of the exponential rate constant k. Values are means ± SEM of 4–15 single shots (time course curves) for each of 4–6 different experiments. (**A**) *, *p* < 0.05 vs. Ctr, Pre084, RC-33; #, *p* < 0.05 vs. Ctr (Repeated measures ANOVA, followed by Newman–Keuls *Q* test). Right: a, *p* < 0.05 vs. Ctr, ASME, RC33. (**B**) *, *p* < 0.05 vs. Ctr (repeated measures ANOVA, followed by Newman–Keuls *Q* test).

As an orthogonal assay to determine the functional profile of our compounds, the effect on AQP-mediated water permeability was investigated. AQPs are a family of transmembrane proteins involved in the transport of water and other small molecules (e.g., H_2_O_2_) through cell membranes. AQP functions are thus critical in ensuring reactive oxygen species (ROS) scavenging and cell survival [63,64]. Our group recently reported that S1R agonists can restore AQP-mediated water and H_2_O_2_ permeability in heat-stressed HeLa cells, thus counteracting oxidative stress, whereas S1R antagonists are unable to exert this beneficial effect [65]. Recently, the osmotic water permeability of AQPs has been demonstrated to be indicative of H_2_O_2_ permeability [63]. Heat treatment was used as a cell stressor and the measurements highlighted a significant decrease in water permeability in untreated stressed cells. Then, water permeability was measured for the cells pre-incubated with known S1R modulators (i.e., the agonists PRE-084 and RC-33 and the antagonist NE-100) [66,67] and the novel compounds (*R*/*S*)-**1a**, (*R*/*S*)-**1c**, (*R*/*S*)-**1d**, and (*R*/*S*)-**2d**. As reported in Figure 5A, the activity of known S1R modulators is consistent with our previous findings [65]. Briefly, the osmotic water permeability of HeLa cells was measured by a stopped-flow light scattering method and expressed as a percentage of k relative. In fact, two distinct profiles can be drawn: the S1R agonists PRE-084 and RC-33 are effective in restoring water permeability in heat-stressed cells, whereas the S1R antagonist NE-100 has no effect. Compounds (*R*/*S*)-**1a**, (*R*/*S*)-**1c**, (*R*/*S*)-**1d**, and (*R*/*S*)-**2d** displayed an effect clearly in line with that of S1R antagonists (Figure 5B). These results are in line with those obtained from the neurite outgrowth assay.

Overall, the effects on neurite outgrowth and AQP-mediated water permeability clearly confirm compounds (*R*/*S*)-**1a**, (*R*/*S*)-**1c**, (*R*/*S*)-**1d**, and (*R*/*S*)-**2d** as S1R antagonists, suggesting that the 2-aryl-4-aminobutanol structure represents a viable scaffold for the development of selective S1R antagonists. Based on the biological results achieved so far, (*R*/*S*)-**1c** and (*R*/*S*)-**1d** were selected for further investigation as they were the S1R antagonists endowed with the highest affinity toward the S1R and highest selectivity over the S2R.

### 2.4. In Silico Developability and Early Toxicity Studies 

In silico developability and early toxicity studies were performed with the aim of identifying the potential liabilities of the compound described herein in order to select the optimal compound for progression toward in vivo proof-of-concept studies and to reduce the attrition rate in the development phase. In this way, it is possible to reduce the number of animals necessary for in vivo investigations, in compliance with the three Rs principle (replacement, reduction, and refinement) [68,69]. 

**Figure 6:**
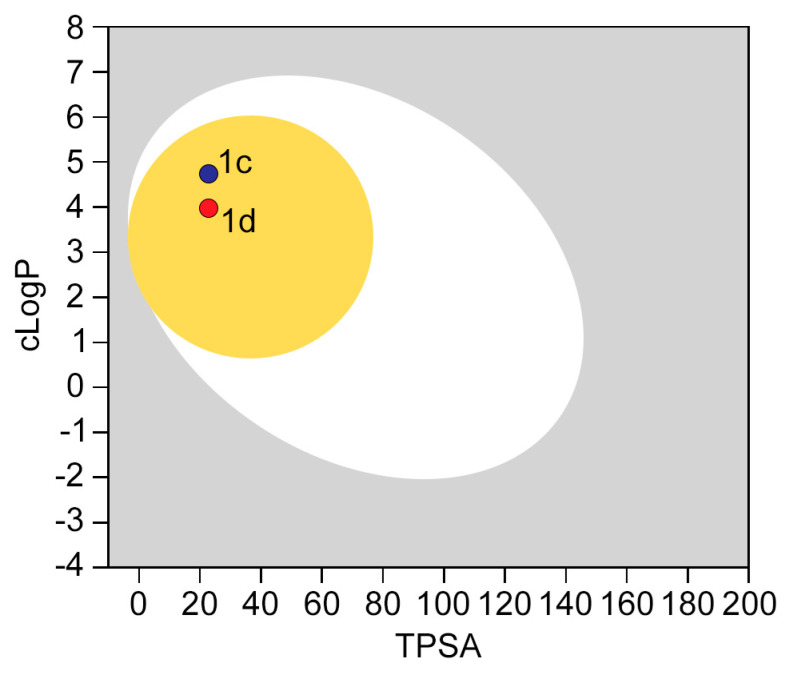
Boiled egg diagram for compounds **1c** and **1d**. The white area of the diagram represents the physicochemical space where molecules are most likely to be absorbed by the gastrointestinal tract, while the yellow area (resembling the yolk) indicates the physicochemical space where molecules are most likely to permeate to the brain. The dots have been color coded to indicate their prediction as a P-gp substrate. Compounds that are predicted to be P-gp substrates are represented in blue, while those that are not predicted to be P-gp substrates are represented in red.

The early in silico estimation of the drug-like properties was performed with the SwissADME free web tool [70]. Solubility, systemic absorption, metabolic stability, and compliance with Lipinski’s rule of five were considered. Particular attention was given to those attributes accounting for a successful CNS drug (i.e., cLogP, TPSA, and P-gp substrate propensity), since these molecules have to cross the blood–brain barrier (BBB) to exert their activity. The predicted physicochemical properties for compounds **1c** and **1d** are given in Figure 6 and Table S1. Briefly, **1d** was predicted to be absorbed per os and to be able to cross the BBB by passive diffusion, indicating an overall balanced physicochemical profile for the optimal brain exposure. Conversely, **1c** was associated with just one violation of Lipinski’s rule of five due to its lipophilicity (cLogP = 4.63), but more importantly, it was predicted to be a potential P-gp substrate, thus limiting its capability to reach pharmacological concentration in the CNS. 

To further support the choice of the best candidate to use in the in vivo studies on mice, the in vitro and in vivo toxicity, as well as the metabolic stability, were experimentally evaluated. 

The early toxicity assays included in vitro cytotoxicity against two human cell lines, namely MRC-5 (human normal lung fibroblasts) and CHME-5 (human microglial cells) and an in vivo zebrafish model. The cell lines MRC-5 and CHME-5 were exposed for 24, 48, and 72 h to increasing concentrations of the tested compounds (from 0.1 to 100 µM). As reported in Figure S1, no cytotoxicity was observed for either compound (*R*/*S*)-**1c** or compound (*R*/*S*)-**1d** at concentrations ≤10 μM. However, at the highest concentration of 100 μM, both compounds reduced the survival of CHME-5 and MRC-5 cells at all time points. Compound (*R*/*S*)-**1c** already showed evident cytotoxic effects at 100 µM after 24 h of exposure in both cell lines. Conversely, compound (*R*/*S*)-**1d** showed a slightly safer profile since no significative cytotoxicity in the CHME-5 and MRC-5 cell lines was observed after 24 and 48 h of exposure, and a survival rate higher than 50% was still detected at 72 h (Figure S1).

**Figure 7:**
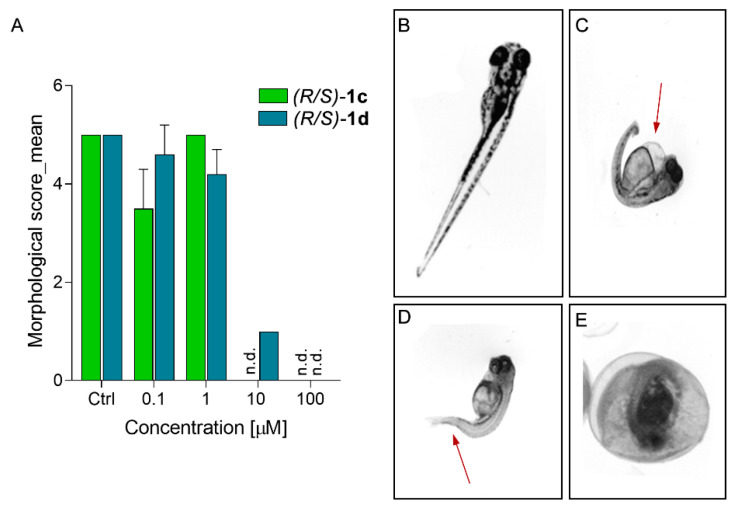
Morphological score (**A**) obtained from the average values of the sample score sheet (Table S2) and representative body shape morphology of zebrafish observed during treatment with a high dose of compounds (*R*/*S*)-**1c** and (*R*/*S*)-**1d:** normal body shape. The experiment was performed in octuplicate, and data were assessed using a sample score sheet, as reported by Brannen KC and colleagues (**B**); abnormal hearth morphology: heart severely enlarged (**C**); abnormal body shape: severely curved tail (**D**); abnormal development: embryo died before complete development (**E**). The main morphological alterations observed are pointed out with red arrow. Exposure time: 120 h, 5 dpf.

The in vivo toxicity was evaluated using the predictive zebrafish animal model, which is suitable for screening drugs for potential toxicity in the nervous, cardiovascular, and digestive system [71]. Zebrafish embryos were exposed to increasing concentrations (from 0.1 to 100 μM) of compounds (*R*/*S*)-**1c** and (*R*/*S*)-**1d** immediately after the eggs’ fertilization. The mortality rate and the kind of developmental deficiencies were evaluated at 5 days post-fertilization (dpf). As reported in Figure 7A, a slight dose-dependent toxicity in the zebrafish embryos was observed for both compounds. All the embryos exposed to the lowest concentrations of the test compounds (0.1 μM and 1 μM) showed a normal and vital phenotype compared to the control. A decrease in the embryo vitality rate and an increase in the entity of damages to the vital structures became more evident at higher concentrations (Figure 7A, Table S2). Compound (*R*/*S*)-**1c** induced embryo death (100% of embryos) at 10 μM, whereas (*R*/*S*)-**1d** caused the death of 5 embryos out of 10 at the same concentration. Moreover, among the living embryos, morphological abnormalities, mainly affecting the shape of the tail, heart, and skin pigmentation, were observed (Figure 7B–E, Table S2). Th embryos showed short and up-curved tails, an enlarged volume of heart, and poor pigmentation. Furthermore, the swim bladder was not well inflated, and oedema was evident. Lastly, both compounds induced the death of all the zebrafish embryos at the highest dose tested (100 μM). 

Compound (*R*/*S*)-**1c** at low concentrations (0.1 μM) caused a minimal variation in morphology (score < 4) that was related to a potentially recoverable developmental delay or anomaly. Conversely, compound (*R*/*S*)-**1d** did not cause evident (score 5) or slight alteration (score 4) of anatomical structures at 0.1 μM or 1 μM, respectively, and induced severe and multiple malformations (score 1) only at 10 μM (Figure 7A). 

To sum up, these results suggested a very low toxicity profile of both compounds at the doses employed; the compounds showed their S1R antagonist activity in the functional assay (i.e., <2.5 μM), with compound (*R*/*S*)-**1d** possessing an overall safer profile compared to (*R*/*S*)-**1c**.

To sum up, at concentrations <2.5 μM both compounds showed a clear S1R antagonist activity, and in the same concentration range they presented a very low toxicity profile, with compound (*R*/*S*)-**1d** possessing an overall safer profile compared to (*R*/*S*)-**1c**.

The in vitro metabolic stability of compound (*R*/*S*)-**1d** was evaluated in mouse plasma and hepatic microsomes. It demonstrated a good metabolic stability in mouse plasma, with approximately 100% of the compound remaining unaltered after 240 min of incubation. Conversely, it exhibited a high clearance in mouse hepatic microsomes under oxidative conditions (Cli of 192 ± 7 µL/min/mg and a t_1/2_ of 7.2 ± 0.3 min) (Figure S2). 

The overall good profile as a safe and effective S1R antagonist, as well as the metabolic stability, prompted us to select (*R*/*S*)-**1d**, called (*R*/*S*)-**RC**-**752** from now on, as a suitable candidate for further in vivo proof-of-concept characterization. 

Of note, our stringent preclinical funnel workflow allowed us to operate in accordance with the three Rs principle: computational simulations and in vitro models (on target, on cell, and on zebrafish models) were exploited to replace the use of animals in the identification of the most promising candidate. Accordingly, we selected just one compound to be tested in vivo, thus reducing the number of animals necessary to achieve reliable results. Lastly, according to the refinement principle, we planned and performed the in vivo experiments while ensuring that the stress caused to the animals was kept as low as possible (e.g., using anesthesia during animal surgery in Chung’s model, as described in the experimental section) and weighing it up against the gain in knowledge and the potential benefits.

### 2.5. In Vivo Antinociceptive Studies

#### 2.5.1. Assessment of the Antinociceptive Activity in the Formalin Test

**Figure 8:**
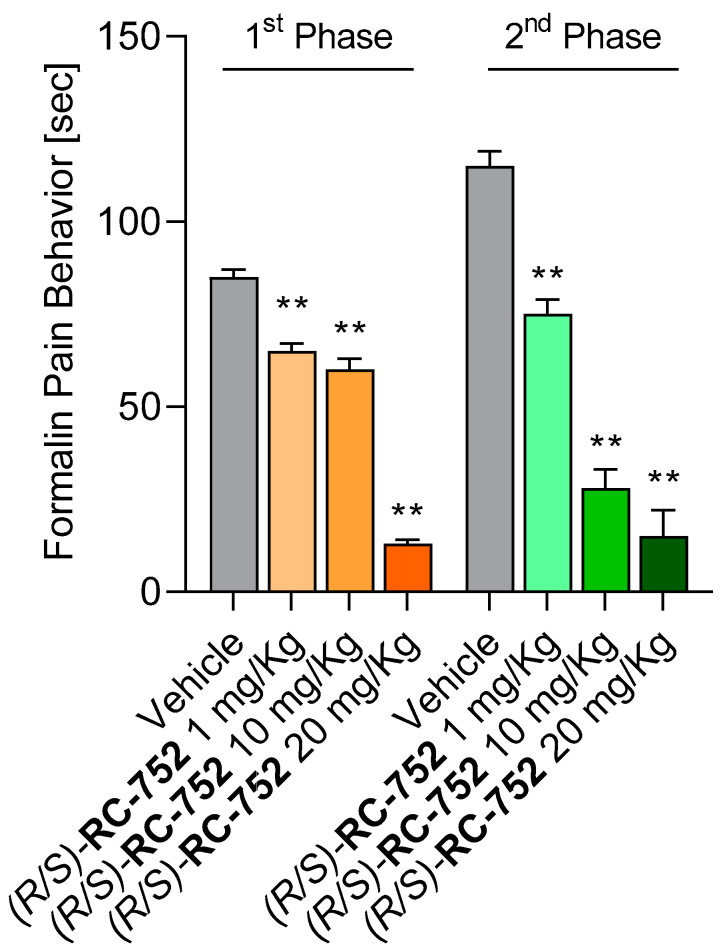
Effect of intraperitoneal (i.p.) injection of (*R*/*S*)-**RC**-**752** (1, 10, or 200 mg/kg) on the first (0−5 min) and second (20−30 min) phases of the formalin test, measured as time spent by the mice in paw licking. The test compound or vehicle was injected 30 min before the intraplantar injection of formalin. The data are expressed as the means ± SEM of 8 mice per group. Two-way ANOVA followed by post hoc Bonferroni test: ** *p* < 0.01 vs. the respective groups of mice injected with the vehicle.

The formalin test was chosen as a tonic pain model for the assessment of the potential analgesic activity of (*R*/*S*)-**RC**-**752** in mice. This is a model of persistent pain in which the nociceptive response produced by formalin is quantified by measuring the time spent by the mouse licking the injected paw. The intraplantar administration of formalin (2%, 20 μL) produces a biphasic nocifensive behavioral response (i.e., biting or licking the injected hind paw). The acute nociceptive phase, reflecting the chemical activation of sensory C-fibers, lasts for the first 5 min, while the second inflammatory phase takes place between 20 and 30 min and corresponds with the development of nociceptive sensitization in the dorsal horn of the spinal cord. (*R*/*S*)-**RC**-**752** was administered 30 min before formalin, at doses of 1, 10, and 20 mg/Kg intraperitoneally (i.p.). As reported in Figure 8, (*R*/*S*)-**RC**-**752** showed an undeniable dose-dependent antinociceptive activity during both the first and the second phases of the formalin test, resulting in an ED_50_ of 13.03 mg/Kg and 2.17 mg/Kg, respectively. These data show that (*R*/*S*)-**RC**-**752** was more effective in decreasing the inflammatory response than the acute activation of nociceptive fibers (first phase) and thus led to a facilitated state of the nociceptive system (second phase).

#### 2.5.2. Assessment of the Antinociceptive Activity in Chung’s Neuropathic Pain Model

To further confirm the antinociceptive activity of (*R*/*S*)-**RC**-**752**, Chung’s neuropathic pain model (or ligation of the spinal nerve test) was exploited.

**Figure 9:**
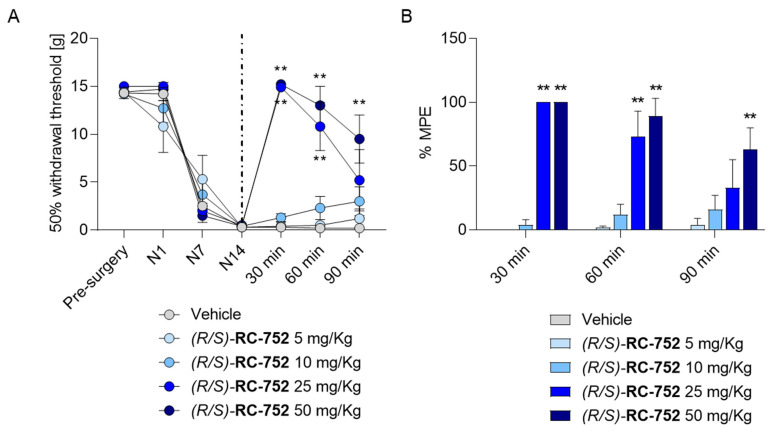
Effect of (*R*/*S*)-**RC**-**752** on nociceptive reaction assessed using the spinal nerve ligation model in rats. (**A**) The mechanical allodynia was induced using von Frey filaments and was expressed as 50% of paw withdrawal before surgery and at 1, 7, and 14 days after nerve ligation (N1, N7, and N14) and at 30, 60, and 90 min after administration of vehicle or (*R*/*S*)-**RC**-**752** at 5, 10, 25, and 50 mg/Kg. The dotted line represents the beginning of the treatment with test compound. (**B**) The antinociceptive activity of 5, 10, 25, and 50 mg/Kg of (*R*/*S*)-**RC**-**752** at 30, 60, and 90 min after administration was expressed as percentage of maximum possible effect (MPE). The data are expressed as the means ± SEM. One-way ANOVA followed by Bonferroni’s post hoc test: ** *p* < 0.01 vs. the respective groups of rats injected with the vehicle.

This procedure involves the surgical binding or tying off of the left L5 and L6 spinal nerves in rats and causes long-lasting behavioral signs of mechanical allodynia and ongoing pain. Mechanical allodynia refers to the perception of pain caused by normally non-painful stimuli, such as touch or pressure. After two weeks of the surgery (N14, Figure 9A), the rats were treated with the vehicle or compound (*R*/*S*)-**RC**-**752**, administered i.p. at doses of 5, 10, 25, and 50 mg/Kg. Mechanical stimulation was then induced using von Frey filaments, a set of calibrated nylon fibers that are used to apply mechanical pressure to the skin or other tissues to measure pain sensitivity. The mechanical threshold for pain was established with the “up and down method”. The effectiveness of the potentially pain-relieving (*R*/*S*)-**RC**-**752** was evaluated 30, 60, and 90 min after compound administration. The results clearly indicated a dose-dependent antinociceptive effect promoted by (*R*/*S*)-**RC**-**752** (Figure 9). No significative effect was observed at the lower doses of 5 and 10 mg/Kg. Conversely, at the doses of 25 and 50 mg/Kg, (*R*/*S*)-**RC**-**752** was able to restore the normal nociceptive functionality (comparable to the value before surgery) at 30 min after administration, and the antinociceptive effect was sustained up to 60 min. At the highest dose (50 mg/Kg), this antinociceptive effect was significatively extended up to 90 min. 

To sum up, both the formalin test and Chung’s model indicated that (*R*/*S*)-**RC**-**752** exerted antinociceptive effects in a dose-dependent manner. Further characterization and deepening of the pharmacological profile of **RC**-**752** will be needed as it enters the lead optimization stage. One point that deserves to be investigated is the potential sex specificity in the analgesic effects of our lead compound. In fact, it has been reported that the S1R is involved in sex-specific heat shock response in a rat model of renal ischemia/reperfusion injury [72]. However, in the case of pain-signaling modulation by the S1R, other studies suggest that there is no significant difference between the sexes [20]. Therefore, the use of male mice only, in the present work, can be considered a reasonable compromise for the obtainment of the in vivo proof of concept of the efficacy of (*R*/*S*)-**RC**-**752**.

## 3. Conclusions

In the present work, we developed a new series of selective S1R antagonists based on a 2-aryl-4-aminobutanol scaffold. Compound (*R*/*S*)-**RC**-**752** emerged as the most interesting compound, showing the most balanced profile in terms of pharmacological activity and developability. It has high affinity toward the S1R, selectivity over the S2R subtype, and a clear antagonist profile, as assessed by the NGF-induced neurite outgrowth assay in PC12 cells and AQP-mediated water permeability experiments in HeLA cells. Moreover, (*R*/*S*)-**RC**-**752** exhibited no cytotoxic effect in the two normal human cell lines or in the in vivo zebrafish model and resulted in being stable after incubation in mouse plasma. Therefore, (*R*/*S*)-**RC**-**752** was evaluated in two animal models of NP: the formalin test and the spinal nerve ligation model, showing analgesic effects in both of the animal models, thus furnishing the proof of concept of its efficacy as an antinociceptive agent. 

To conclude, in the present work (*R*/*S*)-**RC**-**752** emerged as the lead compound. As several causes can induce NP, ranging from diabetes to viral infections and chemotherapy, this disabling disorder still represents an unmet medical need. Although two S1R antagonists are currently under clinical evaluation, expanding the drug candidates against NP increases the chances of finding an effective and safe therapy for each type of NP while taking into account the peculiar status of the patient. Accordingly, future perspectives include the further optimization of the (*R*/*S*)-**RC**-**752** structure to enhance its metabolic stability and the designing and evaluating of a small number of new analogs constructed to limit major metabolic liabilities, while maintaining strong interactions with the target and S1R antagonist profile, with the final aim being the identification of novel antinociceptive agents. 

## 4. Experimental Section

### 4.1. Chemistry

#### General procedures

The reagents and solvents for the synthesis, TLC, and NMR analyses were purchased from commercial suppliers (Merck, Milan, Italy) and used without further purification. Silica gel for flash chromatography (60 Å; 230–400 Mesh) was purchased from Merk. Solvents were evaporated at reduced pressure with the Heidolph Laborota 4000 Efficient equipment. Analytical thin layer chromatography (TLC) analyses were carried out on silica gel pre-coated glass-backed plates (TLC Silica Gel 60 F_254_; Merk) impregnated with a fluorescent indicator and visualized with the instrument MinUVIS DESAGA^®^ Sarstedt-GRUPPE by ultraviolet (UV) radiation from a UV lamp (λ = 254 and 366 nm) or by stain reagents such as ninhydrin. NMR was measured at room temperature (15–25 °C) on a Bruker Advance 400 MHz spectrometer, using tetramethyl silane (TMS) as an internal standard and a BBI 5 mm probe. All raw FID files were processed with the Top Spin program from Bruker and the spectra were analyzed using the MestReNova 6.0.2 program from Mestrelab Research S.L. Chemical shifts are expressed in parts per million (ppm; δ scale). The ^1^H-NMR spectroscopic data were reported as follows: chemical shift in ppm (multiplicity; coupling constants *J* (Hz), integration intensity). The multiplicities are abbreviated with s (singlet), d (doublet), t (triplet), q (quartet), m (multiplet), and brs (broad signal). The chemical shift of all the symmetric signals is reported as the center of the resonance range. The ^13^C-NMR spectroscopic data were reported as follows: chemical shift in ppm.

All the reactions performed under inert conditions were conducted with dry glassware, previously flamed with a Bunsen burner, and fitted with a rubber septum, under a nitrogen atmosphere. Liquid reagents and air/moisture-sensitive and dry solvents were added using plastic syringes with a metal needle that was previously conditioned with nitrogen. Solid reagents were transferred by opening the rubber septum under a nitrogen flow or solubilizing them in the appropriate dry solvents. Low temperatures were reached with cooling agents, such as ice (0 °C), a mixture of ice, methanol (MeOH), and sodium chloride (NaCl) (−18 °C), or a mixture of solid carbon dioxide and acetone (−78 °C), that were placed in a Dewar flask suitable for the reaction. All the reactions conducted under microwave irradiation were performed in a microwave mono-mode oven specifically for organic synthesis (Discover^®^ LabMate instrument, CEM Corporation, Matthews, NC, USA). The progress and ending of the reaction were monitored by TLC; in addition, the final products were analyzed with ^1^H and ^13^C nuclear magnetic resonance (NMR).

The purity of the final compounds was assessed by UPLC-UV-ESI/MS. Analyses were carried out on an Aquity UPLC Waters LCQ FLEET system using an ESI source operating in positive ion mode, controlled by ACQUIDITY PDA and 4 MICRO (Waters). Analyses were run on an ACQUITY BEH C18 (50 × 2.1 mm, 1.7 µm particle size) column at 40 °C with a gradient elution (solvent A: water containing 0.1% of formic acid; solvent B: methanol containing 0.1% of formic acid; gradient: 10% B in A to 100% B in 3 min, followed by isocratic elution 100% B for 1.5 min, with return to the initial conditions in 0.2 min) at a flow rate of 0.5 mL/min. The chromatograms were recorded at 254 nm wavelength, unless otherwise specified. All the final synthesized compounds had 95% or higher purity. The optical rotation values were measured on the Jasco photoelectric polarimeter DIP 1000 with a 0.5 dm quartz cell.

##### General Procedure for the Synthesis of **4**–**6**

AcOEt (2 equiv.), Bu_4_NCl (2 equiv.), and Pd(OAc)_2_ (0.05 equiv.) are placed in a two-necked round-bottom flask, previously dried and purged with N_2_. The reactants are dissolved in dry DMF (40 mL), and ethyl crotonate (1.5 equiv.) is added under vigorous stirring. Then, a solution of the aryl bromide (1 equiv.) in dry DMF (20 mL) is added dropwise through a dropping funnel while increasing the temperature from 50 °C to 75 °C over 15 min. Upon completion of the aryl bromide addition, the temperature is further increased to 105 °C, and the reaction is kept under stirring until completion (3–5 h), which is determined via TLC (n-hex/AcOEt 96:4). Afterwards, the reaction mixture is diluted with Et_2_O (100 mL) and extracted with water (3 × 160 mL). The aqueous phase is then re-extracted with Et_2_O. The reunited organic phases are dried over Na_2_SO_4_, filtered, and concentrated under reduced pressure. Finally, the crude is purified through flash chromatography on silica gel, eluting with n-hex/AcOEt 96:4.

##### Synthesis of ethyl (E)-3-([1,1’-biphenyl]-4-yl)but-2-enoate (**4**)

White solid. Yield: 59%; mp 82.0−84.0 °C; Rf = 0.30 (n-hex/AcOEt 96:4). ^1^H-NMR (400 MHz, CDCl_3_): δ 1.36 (t, *J* = 7.1 Hz, 3H), 2.65 (d, *J* = 1.2 Hz, 3H), 4.26 (q, *J* = 7.1 Hz, 2H), 6.24 (d, *J* = 1.2 Hz, 1H), 7.39 (t, *J* = 7.3 Hz, 1H), 7.48 (t, *J* = 7.6 Hz, 2H), 7.61 (m, 6H). ^13^C-NMR (101 MHz; CDCl_3_): δ 14.38, 17.81, 59.88, 116.99, 126.79, 127.05, 127.16, 127.65, 128.87, 140.30, 140.97, 141.86, 154.90, 166.90.

##### Synthesis of ethyl (E)-3-(naphthalen-2-yl)but-2-enoate (**5**)

White solid. Yield: 53%; mp 54.0 °C; Rf = 0.31 (n-hex/AcOEt 96:4). ^1^H-NMR (400 MHz, CDCl_3_): δ 1.37 (t, *J* = 7.1 Hz, 3H), 2.72 (d, *J* = 1.3 Hz, 3H), 4.28 (q, *J* = 7.1 Hz, 2H), 6.33 (d, *J* = 1.3 Hz, 1H), 7.52–7.54 (m, 2H), 7.63 (dd, *J* = 8.6, 1.9 Hz, 1H), 7.90–7.84 (m, 3H), 7.98 (d, *J* = 1.6 Hz 1H). ^13^C-NMR (101 MHz; CDCl_3_): δ 14.40, 17.93, 59.91, 117.55, 123.97, 125.94, 126.50, 126.69, 127.59, 128.15, 128.51, 133.14, 133.50, 139.38, 155.23, 166.90.

##### Synthesis of ethyl (E)-3-(6-methoxynaphthalen-2-yl)but-2-enoate (**6**)

White solid. Yield: 51%; mp 78.0 °C; Rf = 0.21 (n-hex/AcOEt 96:4). ^1^H-NMR (400 MHz, CDCl_3_): δ 1.36 (t, *J* = 7.1 Hz, 3H), 2.70 (d, *J* = 1.2 Hz, 3H), 3.95 (s, 3H), 4.26 (q, *J* = 7.1 Hz, 2H), 6.30 (d, *J* = 1.3 Hz, 1H), 7.14 (d, *J* = 2.5 Hz, 1H), (m, 2H), 7.18 (dd, J=8.9, 2.5 Hz, 1H), 7.61 (dd, *J* = 8.6, 1.9 Hz, 1H), 7.73 (d, *J* = 8.7 Hz, 1H), 7.77 (d, *J* = 8.9 Hz, 1H) 7.91 (d, *J* = 1.6 Hz, 1H). ^13^C-NMR (101 MHz; CDCl_3_): δ 14.40, 17.78, 55.35, 59.83, 105.57, 116.64, 119.36, 124.45, 125.81, 126.94, 128.55, 130.06, 134.84, 137.04, 155.28, 158.38, 167.02.

##### General Procedure for the Synthesis of **7***–***9**

One gram of α,β-unsaturated ester (**4**–**6**) is placed in an 80 mL vessel for microwave (mw) irradiation and solubilized with CH_3_CN (30 mL). SeO_2_ (1.3 equiv.) is added to the reaction mixture, and the vessel is subject to two heating cycles in the mw oven under the following conditions: T = 100 °C, W = 180, PSI = 200, run time = 15 min each cycle. The consumption of the starting material is monitored via TLC eluting with n-hex/AcOEt 90:10, whereas the formation of the unsaturated lactone is revealed by eluting with 100% DCM. The reaction mixture is filtered on a paper filter and concentrated under reduced pressure. The solid obtained is washed with Et_2_O at 0 °C, then solubilized in AcOEt at r.t., filtered, and concentrated again under reduced pressure. The crude thus obtained is subjected to the subsequent reaction without further purification. An analytic sample was purified for spectroscopic characterization.

##### Synthesis of 4-([1,1’-biphenyl]-4-yl)furan-2(5H)-one (**7**)

Yellow solid. Rf = 0.38 (n-hex/AcOEt 70:30). ^1^H-NMR (400 MHz, CDCl_3_): δ 5.27 (d, *J* = 1.7 Hz, 2H), 6.43 (t, *J* = 1.7 Hz, 1H), 7.41–7.53 (m, 3H), 7.59–7.67 (m, 4H), 7.69–7.73 (m, 2H). ^13^C-NMR (101 MHz; CDCl_3_): δ 71.03, 112.84, 126.98, 127.09, 127.87, 128.33, 128.45, 129.06, 139.59, 144.57, 163.48, 173.91.

##### Synthesis of 4-(naphthalen-2-yl)furan-2(5H)-one (**8**)

Yellow powder. Rf = 0.36 (n-hex/AcOEt 70:30). ^1^H-NMR (400 MHz, CDCl_3_): δ 5.35 (d, *J* = 1.7 Hz, 2H), 6.48 (t, *J* = 1.7 Hz, 1H), 7.56–7.63 (m, 3H), 7.88–7.94 (m, 4H). ^13^C-NMR (101 MHz; CDCl_3_): δ 71.11, 113.31, 123.27, 126.45, 127.00, 127.30, 127.95, 128.16, 128.77, 129.25, 132.88, 134.67, 163.76, 173.92.

##### Synthesis of 4-(6-methoxynaphthalen-2-yl)furan-2(5H)-one (**9**)

Yellow powder. Rf = 0.38 (n-hex/AcOEt 70:30). ^1^H-NMR (400 MHz, CDCl_3_): δ 3.96 (s, 3H), 5.32 (s, 2H), 6.42 (s, 1H), 7.17–7.24 (m, 2H), 7.58 (d, *J* = 8.4 Hz, 1H), 7.75–7.86 (m, 3H). ^13^C-NMR (101 MHz; CDCl_3_): δ 55.47, 71.07, 106.00, 112.13, 120.17, 123.88, 124.79, 126.26, 127.93, 128.24, 130.32, 136.30, 159.48, 163.94, 174.14.

##### General Procedure for the Synthesis of **10***–***12**

The unsaturated lactone (**7**–**9**) is placed in an 80mL vessel suitable for mw irradiation and solubilized in AcOEt (25mL) under gentle heating. Then, *t*-BuOH (7.9 mL/mmol), HCO_2_NH_4_ (5 equiv.), and Pd/C (35% wt) are added to the vessel. The reaction mixture is subjected to mw irradiation under the following conditions: T = 100 °C, W = 180, PSI = 200, run time = 5 min. The formation of the desired product is monitored via TLC (n-hex/AcOEt 70:30). Upon completion, the reaction is filtered on a paper filter and concentrated at reduced pressure. The crude is purified by flash chromatography on silica gel, eluting with n-hex/AcOEt 70:30.

##### Synthesis of 4-([1,1’-biphenyl]-4-yl)dihydrofuran-2(3H)-one (**10**)

White powder. Yield over two steps: 53%; Rf = 0.37 (n-hex/AcOEt 70:30). ^1^H-NMR (400 MHz, CDCl_3_): δ 2.74 (dd, *J* = 17.5, 8.4 Hz, 1H), 2.98 (dd, *J* = 17.5, 8.4 Hz, 1H), 3.86 (q, *J* = 8.4 Hz, 1H), 4.33 (t, *J* = 8.4 Hz, 1H), 4.72 (t, *J* = 8.4 Hz, 1H), 7.32–7.41 (m, 3H), 7.43–7.52 (m, 2H), 7.55–7.65 (m, 4H). ^13^C-NMR (101 MHz; CDCl_3_): δ 35.74, 40.84, 74.00, 127.03, 127.15, 127.55, 127.83, 128.87, 138.40, 140.35, 140.77, 176.30.

##### Synthesis of 4-(naphthalen-2-yl)dihydrofuran-2(3H)-one (**11**)

White powder. Yield over two steps: 33%; Rf = 0.37 (n-hex/AcOEt 70:30). ^1^H-NMR (400 MHz, CDCl_3_): δ 2.81 (dd, *J* = 17.5, 8.3 Hz, 1H), 3.02 (dd, *J* = 17.5, 8.3 Hz, 1H), 3.97 (q, *J* = 8.3 Hz, 1H), 4.40 (dd, *J* = 9.0, 8.3 Hz, 1H), 4.76 (dd, *J* = 9.0, 8.3 Hz, 1H), 7.36 (dd, *J* = 8.6, 1.8 Hz, 1H), 7.48–7.56 (m, 2H), 7.69 (s, 1H), 7.81–7.93 (m, 3H). ^13^C-NMR (101 MHz; CDCl_3_): δ 35.69, 41.22, 73.94, 124.47, 125.51, 126.26, 126.69, 127.69, 127.72, 129.15, 132.71, 133.41, 136.77, 176.36.

##### Synthesis of 4-(6-methoxynaphthalen-2-yl)dihydrofuran-2(3H)-one (**12**)

White powder. Yield over two steps: 31%; Rf = 0.31 (n-hex/AcOEt 70:30). ^1^H-NMR (400 MHz, CDCl_3_): δ 2.79 (dd, *J* = 17.5, 8.3 Hz, 1H), 3.00 (dd, *J* = 17.5, 8.3 Hz, 1H), 3.87–4.01 (m, 4H), 4.37 (t, *J* = 8.3 Hz, 1H), 4.74 (t, *J* = 8.3 Hz, 1H), 7.15 (s, 1H), 7.20 (d, *J* = 8.9 Hz, 1H), 7.32 (d, *J* = 8.4 Hz, 1H), 7.61 (s, 1H), 7.73 (d, *J* = 8.6 Hz, 1H), 7.78 (d, *J* = 8.6 Hz, 1H). ^13^C- NMR (101 MHz; CDCl_3_): δ 35.75, 41.10, 55.35, 74.08, 105.66, 119.54, 124.97, 125.34, 127.91, 128.85, 129.15, 133.89, 134.35, 157.95, 176.47.

##### General Procedure for the Synthesis of **13a***–***15d**

A solution of amine **a**–**d** (2.5 equiv.) in 1,2-dichloroetane (DCE, 2.9 mL) is added dropwise to a flask containing AlCl_3_ (1.3 equiv.) under stirring at 0 °C. The reaction mixture is then heated at r.t. and, after 15 min, a solution of the saturated lactone **10**–**12** (1 equiv.) in DCE (6.3 mL) is added. The reaction is monitored via TLC (eluting with n-hex/AcOEt 70:30 to verify consumption of the starting material and with AcOEt/MeOH 90:10 to detect the target product) and 5 or more equiv. of amine **a**–**d** are added, if necessary, to bring the reaction to completion. After 3–5 h the mixture is diluted with DCM and ice-cold water (5 and 10 mL, respectively), maintained under stirring for 30 min, and then filtered through celite and concentrated in vacuo. The crude is finally purified by flash chromatography on silica gel, eluting with n-hexane/EtOAc 70:30 and then n-hexane/EtOAc 95:5. 

##### Synthesis of 3-([1,1’-biphenyl]-4-yl)-4-hydroxy-1-morpholinobutan-1-one (**13a**)

White solid. Yield: 60%; Rf = 0.46 (AcOEt/MeOH 90:10). ^1^H-NMR (400 MHz, CDCl_3_): δ 2.76 (dd, *J* = 15.5 Hz, 5.5 Hz, 1H) 2.87 (dd, 15.5 Hz, 8.0 Hz, 1H) 3H), 2.90–3.10 (br 1H) 3.37–3.57 (m, 4H) 3.58–3.74 (m, 5H) 3.85 (dd, *J* = 10.9 Hz, 7.2 Hz, 1H) 3.91 (dd, *J* = 10.9 Hz, 5.5 Hz, 1H) 7.26–7.41 (m, 3H) 7.43–7.50 (m, 2H) 7.54–7.65 (m, 4H). ^13^C-NMR (101 MHz; CDCl3): δ 36.59, 42.15, 44.00, 46.25, 66.47, 66.82, 67.23, 127.01, 127.31, 127.49, 128.09, 128.80, 140.03, 140.68, 141.05, 170.96.

##### Synthesis of 3-([1,1’-biphenyl]-4-yl)-N,N-diethyl-4-hydroxybutanamide (**13b**)

Yellow solid. Yield: 78%; Rf = 0.63 (AcOEt/MeOH 90:10). ^1^H-NMR (400 MHz, CDCl_3_): δ 1.13 (t, *J* = 6.8 Hz, 3H), 1.16 (t, *J* = 6.8 Hz, 3H), 2.78 (dd, *J* = 15.4 Hz, 4.7 Hz, 1H), 2.87 (dd, *J* = 15.4, 8.7 Hz, 1H), 3.30 (q, J = 7.2 Hz, 2H) 3.40 (q, *J* = 7.2 Hz, 2H) 3.45–3.55 (m, 2 H) 3.85 (dd, *J* = 10.8 Hz,7.4 Hz, 1H) 3.90 (dd, *J* = 10.8 Hz, 5.2 Hz, 1H) 7.31–7.39 (m, 3H) 7.42–7.50 (m, 2H) 7.54–7.64 (m, 4H). ^13^C-NMR (101 MHz; CDCl_3_): δ 12.99, 14.29, 37.37, 40.62, 42.36, 44.00, 67.71, 127.01, 127.23, 127.41, 128.01, 128.77, 139.81, 140.78, 141.66, 171.72.

##### Synthesis of 3-([1,1’-biphenyl]-4-yl)-N-benzyl-4-hydroxy-N-methylbutanamide (**13c**)

White solid. Yield: 93%; Rf = 0.67 (AcOEt/MeOH 90:10). ^1^H-NMR (400 MHz, CDCl_3_): δ 2.85–3.01 (m, 6H), 3.58 (br s, 1H), 3.84–3.96 (m, 2H), 4.54–4.69 (m, 2H), 7.07–7.65 (m, 14H). ^13^C-NMR (101 MHz; CDCl_3_): δ 34.23, 35.13, 36.93, 37.31, 43.95, 51.13, 53.54, 67.50, 67.64, 126.30, 127.02, 127.43, 127.96, 128.08, 128.13, 128.63, 128.78, 129.00, 136.23, 136.99, 139.89, 140.77, 141.31, 172.58.

##### Synthesis of 3-([1,1’-biphenyl]-4-yl)-4-hydroxy-1-(piperidin-1-yl)butan-1-one (**13d**)

White solid. Yield: 40%; Rf = 0.60 (AcOEt/MeOH 90:10). ^1^H-NMR (400 MHz, CDCl_3_): δ 142–1.60 (br, 4H) 1.60–1.69 (m, 2H) 2.81 (dd, *J* = 15.5 Hz, 5.0 Hz, 1H) 2.88 (dd, *J* = 15.5 Hz, 8.5 Hz, 1H) 3.30–3.70 (m, 6H) 3.84 (dd, *J* = 10.9 Hz, 7.4 Hz, 1H) 3.90 (dd, *J* = 10.9 Hz, 5.4 Hz, 1H) 7.31–7.39 (m, 3H) 7.42–7.49 (m, 2H) 7.53–7.64 (m, 4H). ^13^C-NMR (101 MHz; CDCl_3_): δ 24.43, 25.53, 26.40, 37.36, 43.03, 43.96, 46.98, 67.69, 127.02, 127.23, 127.44, 128.04, 128.77, 139.86, 140.80, 141.57, 170.72.

##### Synthesis of 4-hydroxy-1-morpholino-3-(naphthalen-2-yl)butan-1-one (**14a**)

White powder. Yield: 69%; Rf = 0.47 (AcOEt/MeOH 90:10). ^1^H-NMR (400 MHz, CDCl_3_): δ 2.77–2.85 (m, 2H), 2.93 (br dd, *J* = 15.4 Hz, 7.9 Hz, 1H), 3.35–3.51 (br m, 3H), 3.54–3.69 (br m,6H), 3.87–4.01 (br m,2H), 7.39 (d, J = 8.4 Hz, 1H), 7.44–7.53 (br m, 2H), 7.70 (br s, 1H), 7.78–7.87 (br m, 3H). ^13^C-NMR (101 MHz, CDCl_3_): δ 36.58, 42.13, 44.45, 46.23, 66.44, 66.79, 67.16, 125.79, 125.97, 126.20, 126.29, 127.63, 127.65, 128.52, 132.54, 133.49, 139.42, 170.94.

##### Synthesis of N,N-diethyl-4-hydroxy-3-(naphthalen-2-yl)butanamide (**14b**)

White solid. Yield: 99%; Rf = 0.64 (AcOEt/MeOH 90:10). ^1^H-NMR (400 MHz, CDCl_3_): δ 1.14 (br d, *J* = 6.6 Hz, 6H), 2.83–2.97 (m, 3H), 3.28 (br d, *J* = 6.4 Hz, 2H), 3.40 (br d, *J* = 6.4 Hz, 2H), 3.62 (br s, 1H), 3.89–3.97 (m, 2H), 7.40 (d, *J* = 8.2 Hz, 1H), 7.43–7.53 (m, 2H), 7.70 (br s, 1H), 7.76–7.89 (m, 3H). ^13^C-NMR (101 MHz, CDCl3): δ 12.97, 14.27, 37.41, 40.65, 42.38, 44.46, 67.65, 125.66, 126.03, 126.18, 127.62, 127.66, 128.44, 132.48, 133.51, 140.02, 171.74.

##### Synthesis of N-benzyl-4-hydroxy-N-methyl-3-(naphthalen-2-yl)butanamide (**14c**)

White solid. Yield: 50%; Rf = 0.69 (AcOEt/MeOH 90:10). ^1^H-NMR (400 MHz, CDCl_3_): δ 2.80–3.03 (m, 6H), 3.69 (br s, 1H), 3.86–3.98 (m, 2H), 4.45–4.72 (m, 2H) 7.11 (br s, 2H), 7.18–7-39 (m, 3H), 7.40–7.55 (m, 3H), 7.62–7.89 (m, 4H). ^13^C-NMR (101 MHz, CDCl_3_): δ 15.29, 34.23, 35.16, 36.92, 37.25, 44.44, 44.48, 51.09, 53.53, 65.86, 67.44, 67.52, 125.66, 125.71, 126.04, 126.13, 126.20, 126.27, 127.36, 127.61, 127.64, 127.70, 127.72, 127.87, 128.43, 128.48, 128.58, 128.98, 132.51, 132.55, 133.50, 133.53, 136.23, 136.94, 139.64, 139.67, 172.54, 172.75.

##### Synthesis of 4-hydroxy-3-(naphthalen-2-yl)-1-(piperidin-1-yl)butan-1-one (**14d**)

White solid. Yield: 77%; Rf = 0.61 (AcOEt/MeOH 90:10). ^1^H-NMR (400 MHz, CDCl_3_): δ 1.43–1.65 (m, 6H), 2.86 (dd, *J* = 15.5 Hz, 5.0 Hz, 1H), 2.93 (dd, *J* = 15.5 Hz, 8.5 Hz, 1H3.21 (br s, 1H), 3.36–3.45 (m, 2H), 3.90 (dd, *J* = 11.0 Hz, 7.3 Hz, 1H), 3.94 (dd, *J* = 11.0 Hz, 5.4 Hz, 1H), 7.40 (dd, *J* = 8.5 Hz, 1.6 Hz, 1H), 7.43–7.51(m, 2H), 7.7 (br s, 1H), 7.76–7.86 (m,3H). ^13^C-NMR (101 MHz, CDCl_3_): δ 24.42, 25.52, 26.39, 37.39, 43.01, 44.40, 46.97, 67.63, 125.65, 126.06, 126.17, 127.62, 127.65, 128.43, 132.49, 133.53, 139.99, 170.68.

##### Synthesis of 4-hydroxy-3-(6-methoxynaphthalen-2-yl)-1-morpholinobutan-1-one (**15a**)

White solid. Yield: 78%; Rf = 0.43 (AcOEt/MeOH 90:10). ^1^H-NMR (400 MHz, CDCl_3_): δ 2.79.2.93 (m, 3H), 3.32–3.49 (m, 3H), 3.49–3.71(m, 6H), 3.82–4.01 (m, 2H), 3.92 (s, 3H), 7.12–7.17 (m, 2H), 7.35 (d, *J* = 8.4 Hz, 1H), 7.61 (br s, 1H), 7.68–7.76 (m, 2H). ^13^C-NMR (101 MHz, CDCl_3_): δ 36.65, 42.11, 44.32, 46.24, 55.32, 66.43, 66.78, 67.20, 105.61, 119.13, 126.03, 126.46, 127.35, 128.97, 129.10, 133.65, 136.99, 157.61, 171.02.

##### Synthesis of N,N-diethyl-4-hydroxy-3-(6-methoxynaphthalen-2-yl)butanamide (**15b**)

White solid. Yield: 82%; Rf = 0.60 (AcOEt/MeOH 90:10). ^1^H-NMR (400 MHz, CDCl_3_): δ 1.11 (t, *J* = 7.8 Hz, 3H), 1.13 (t, *J* = 7.8 Hz, 1H), 2.81 (dd, *J* = 15.4, 4.7 Hz, 1H), 2.90 (dd, *J* = 15.4 Hz, 8.5 Hz, 1H), 3.27 (q, *J* = 7.2 Hz, 2H), 3.31–3.52 (br, 1H), 3.39 (q, *J* = 7.0 Hz, 2H), 3.54–3.61 (m, 1H), 3.86–3.97 (m, 2H), 3.92 (s, 3H), 7.17–7.12 (m, 2H), 7.36 (dd, *J* = 8.5 Hz, 1.8 Hz, 1H), 7.71 (d, *J* = 8.4 Hz, 1H), 7.72 (d, *J* = 8.4 Hz, 1H), 7.71 (dd, *J* = 8.5, 4.0 Hz, 2H). ^13^C-NMR (101 MHz, CDCl_3_): δ 12.98, 14.28, 37.47, 40.59, 42.34, 44.30, 55.31, 67.68, 105.60, 118.99, 125.86, 126.55, 127.25, 128.98, 129.14, 133.55, 137.68, 157.53, 171.78.

##### Synthesis of N-benzyl-4-hydroxy-3-(6-methoxynaphthalen-2-yl)-N-methylbutanamide (**15c**)

White powder. Yield: 62%; Rf = 0.63 (AcOEt/MeOH 90:10). ^1^H-NMR (400 MHz, CDCl_3_): δ 2.84–3.23 (m, 6H), 3.61–3.75 (m, 1H), 3.82–4.03 (m, 5H), 4.49 (d, *J* = 16.8 Hz, 0.34 H), 4.54 (d, *J* = 14.7 Hz, 0.66 H), 4.55 (d, *J* =16.8 Hz, 0.34 H), 4.65 (d, *J* =14.7 Hz, 0.66 H), 7.03–7.21(m, 4H), 7.22–7.42 (m, 4H), 7.55–7.75 (m, 3H). ^13^C-NMR (101 MHz; CDCl_3_): δ 34.19, 35.15, 37.01, 37.34, 44.27, 44.30, 51.08, 53.52, 55.33, 67.50, 67.59, 105.60, 105.63, 118.96, 119.01, 125.93, 126.09, 126.28, 126.54, 126.63, 127.26, 127.31, 127.35, 127.68, 127.89, 128.58, 128.96, 128.98, 129.02, 129.18, 129.19, 133.60, 133.63, 136.27, 136.98, 137.27, 137.29, 157.58, 172.62.

##### Synthesis of 4-hydroxy-3-(6-methoxynaphthalen-2-yl)-1-(piperidin-1-yl)butan-1-one (**15d**)

White solid. Yield: 75%; Rf = 0.61 (AcOEt/MeOH 90:10). ^1^H-NMR (400 MHz, CDCl_3_): δ 1.42–1.67 (m, 6H), 2.84 (dd, *J* = 15.5 Hz, 5.0 Hz, 1H), 2.90 (dd, *J* = 15.5 Hz, 8.5 Hz, 1H), 3.11–3.35 (br s, 1H), 3.36–3.45 (m, 2H), 3.50–3.65 (m,3H), 3.88 (dd, *J* = 10.9 Hz, 5.6 Hz, 1H), 3.92 (s, 3H), 3.93 (dd, *J* =10.9 Hz, 5.6 Hz, 1H), 7.17–7.12 (m, 2H), 7.35 (dd, *J* = 8.5, 1.7 Hz, 1H), 7.62 (br s, 1H), 7.70 (d, *J* = 8.8 Hz, 1H), 7.71 (d, *J* = 8.5 Hz, 1H). ^13^C-NMR (101 MHz, CDCl_3_): δ 24.42, 25.52, 26.39, 37.48, 43.00, 44.23, 46.97, 55.31, 67.71, 105.61, 118.99, 125.88, 126.54, 127.26, 129.00, 129.12, 133.57, 137.60, 157.53, 170.76.

##### General Procedure for the Synthesis of **1a***–***3e**

A solution of amide **13a**–**15e** (1 equiv.) in anhydrous THF (2 mL) is added dropwise to a stirred suspension of LiAlH_4_ (2 equiv.) in anhydrous THF (2 mL) at −15 °C. The reaction mixture is stirred at r.t. for 2–4 h and monitored via TLC (AcOEt/MeOH/Et_3_N 90:10:2.5). If necessary, one more equivalent of LiAlH_4_ is added to consume all the starting material. Upon completion, the reaction is cooled to 0 °C, slowly quenched with NH_4_Cl and stirred for 15 min at r.t. Then, diluted HCl is added to the flask until pH < 6, and the aqueous phase is washed with Et_2_O (3 × 30 mL). Na_2_CO_3_ is added to the aqueous phase until pH > 8 and then extracted with AcOEt (3 × 30 mL). The organic phase is dried over Na_2_SO_4_, filtered, and evaporated to dryness. Finally, the crude product is purified by flash chromatography and eluted with EtOAc/MeOH 90:10 and then with EtOAc/MeOH/Et_3_N 90:10:2.5, to yield the pure final compound.

##### Synthesis of 2-([1,1’-biphenyl]-4-yl)-4-morpholinobutan-1-ol (**1a**)

Colorless oil. Yield: 28%; Rf = 0.44 (EtOAc/MeOH/Et_3_N 90:10:2.5). ^1^H-NMR (400 MHz, CDCl_3_): δ 1.90–2.01 (m, 1H), 2.05–2.17 (m,1H), 2.45–2.62 (m, 4H), 2.65–2.78 (br m, 2H), 2.90–2.99 (m, 1H), 3.71–3.78 (m, 2H), 3.81–3.86 (m, 4H), 4.51–6.02 (br, 1H), 7.30 (dt, *J* = 8.2 Hz, 2.1 Hz, 2H), 7.35 (tt, *J* = 7.4 Hz, 1.6 Hz, 1H), 7.45 (tt, *J* = 7.6 Hz, 1.8 Hz, 1.6 Hz, 2H), 7.56 (dt, *J* = 8.2 Hz, 1.7 Hz, 2H), 7.57–7.62 (m, 2H). ^13^C-NMR (101 MHz, CDCl_3_): δ 30.93, 48.28, 53.44, 57.73, 66.41, 67.92, 126.99, 127.19, 127.35, 127.82, 128.75, 139.57, 140.81, 142.46. UPLC-ESI-MS: *m*/*z* calcd for C_20_H_26_NO_2_^+^ [M + H]^+^ 312.20; found: 312.03; t*_R_* = 1.56.

##### Synthesis of 2-([1,1’-biphenyl]-4-yl)-4-(diethylamino)butan-1-ol (**1b**)

Colorless oil. Yield: 86%; Rf = 0.40 (EtOAc/MeOH/Et_3_N 90:10:2.5). ^1^H-NMR (400 MHz, CDCl_3_): δ 0.93–1.37 (br 1H), 1.13 (br t, *J* = 6.7 Hz, 6H), 1.86–2.01 (m, 1H), 2.04–2.15 (m, 1H), 2.51–2.68 (m, 4H), 2.75–2.84 (m, 2H), 2.85–2.96 (m, 1H), 3.65–3.81 (m, 2H), 7.26–7.39 (br m, 3H), 7.44 (br t, *J* = 7.0 Hz, 2H), 7.55 (br d, *J* = 7.4 Hz, 2H), 7.60 (br d, *J* = 7.1 Hz, 2H). ^13^C-NMR (101 MHz, CDCl_3_): δ 10.75, 33.08, 46.28, 49.19, 52.45, 68.20, 127.00, 127.11, 127.24, 127.69, 128.72, 139.29, 140.93, 143.47. UPLC-ESI-MS: *m*/*z* calcd for C_20_H_28_NO^+^ [M + H]^+^ 298.22; found: 298.22; t*_R_* = 1.67. 

##### Synthesis of 3-([1,1’-biphenyl]-4-yl)-N-benzyl-4-hydroxy-N-methylbutanamine (**1c**)

Colorless oil. Yield: 23%; Rf = 0.61 (EtOAc/MeOH/Et_3_N 90:10:2.5). ^1^H-NMR (400 MHz, CDCl_3_): δ 1.67–1.74 (m, 0.35 H), 1.93–2.01 (m, 1H), 2.04–2.18 (m, 1H), 2.26 (s, 3H), 2.51 (dt, *J* = 12.2, 4.7 Hz, 1H), 2.47–2.55 (m, 1H), 2.65–2.74 (m, 1H), 2.87–2.98 (m, 1H), 3.57 (d, *J* = 12.8 Hz,1H), 3.67 (d, *J* =12.8 Hz, 1H), 3.70–3.75 (m, 0.65H), 3.77 (d, *J* = 6.2 Hz, 2H), 7.25–7.41 (m, 8H), 7.45 (t, *J* = 7.8 Hz, 2H), 7.54 (d, *J* = 8.3 Hz, 2H), 7.57–7.62 (m, 2H). ^13^C-NMR (101 MHz, CDCl_3_): δ 32.58, 41.35, 48.53, 56.78, 62.57, 68.00, 127.00, 127.12, 127.27, 127.47, 127.80, 128.49, 128.73, 129.45, 137.30, 139.38, 140.92, 143.04. UPLC-ESI-MS: *m*/*z* calcd for C_24_H_28_NO^+^ [M + H]^+^ 346.22; found: 346.05; t*_R_* = 1.93.

##### Synthesis of 2-([1,1’-biphenyl]-4-yl)-4-(piperidin-1-yl)butan-1-ol (**1d**)

Colorless oil. Yield: 30%; Rf = 0.41 (EtOAc/MeOH/Et_3_N 90:10:2.5). ^1^H-NMR (400 MHz, CDCl_3_): δ 1.51–1.55 (m, 2H), 1.64–1.81 (m, 5H), 1.87–1.98 (m, 1H), 2.06–2.19 (m, 1H), 2.32–2.77 (m, 6H), 2.86–2.94 (m, 1H), 3.68–3.78 (m, 2H), 7.29 (d, *J* = 8.2 Hz, 2H), 7.35 (tt, *J* = 7.3 Hz, 1.6 Hz, 1H), 7.44 (tt, *J* = 7.3 Hz, 1.6 Hz, 2H), 7.54 (dt, *J* = 8.3 Hz, 2.1 Hz, 2H), 7.57–7.62 (m, 2H). ^13^C-NMR (101 MHz, CDCl_3_): δ 24.15, 25.44, 32.45, 49.29, 54.38, 58.15, 68.17, 127.00, 127.10, 127.24, 127.67, 128.72, 139.30, 140.93, 143.46. UPLC-ESI-MS: *m*/*z* calcd for C_21_H_28_NO^+^ [M + H]^+^ 310.22; found: 310.12 t*_R_* = 1.68.

##### Synthesis of 2-([1,1’-biphenyl]-4-yl)-4-(4-benzylpiperidin-1-yl)butan-1-ol (**1e**)

Whitish solid. Yield: 34%; Rf = 0.45 (DCM/MeOH/Et_3_N 95:5:1). ^1^H NMR (400 MHz, CDCl_3_) δ 1.48 (m, 4H), 1.65 (s, 2H), 1.92 (s, 2H), 2.13 (m, 2H), 2.32–2.64 (m, 4H), 2.84 (dt, *J* = 8.7, 4.4 Hz, 1H), 2.99 (m, 1H), 3.20 (d, *J* = 11.5 Hz, 1H), 3.56–3.75 (m, 2H), 6.99–7.10 (m, 2H), 7.14 (d, *J* = 7.3 Hz, 1H), 7.17–7.29 (m, 7H), 7.36 (t, *J* = 7.6 Hz, 2H), 7.48 (dd, *J* = 15.5, 7.5 Hz, 4H). ^13^C NMR (100 MHz, CDCl_3_) δ 30.02, 31.80, 36.82, 39.69, 48.42, 53.13, 54.94, 64.78, 126.40, 126.45, 127.18, 127.90, 128.36, 128.43, 128.44, 129.10, 138.56, 140.58, 140.97, 141.87.UPLC-ESI-MS: *m*/*z* calcd for C_28_H_34_NO^+^ [M + H]^+^ 410.26; found: 410.21 t*_R_* = 1.81.

##### Synthesis of 4-morpholino-2-(naphthalen-2-yl)butan-1-ol (**2a**)

Colorless oil. Yield: 21%; Rf = 0.24 (EtOAc/MeOH/Et_3_N 90:10:2.5). ^1^H-NMR (400 MHz, CDCl_3_): 1.93–2.02 (m, 1H), 2.09–2.20 (m, 1H), 2.43–2.59 (m, 4H), 2.62–2.71 (br m, 2H), 3.00–3.09 (m, 1H), 3.73–3.86 (m, 6H), 7.36 (dd, *J* = 8.5 Hz, 1.8 Hz, 1H), 7.42–7.50 (m, 2H), 7.66 (s, 1H), 7.79–7.84 (m, 3H). ^13^C-NMR (101 MHz, CDCl_3_): δ 31.22, 48.86, 53.56, 57.78, 66.66, 67.90, 125.49, 125.75, 126.00, 126.08, 127.59, 128.26, 132.35, 133.54, 141.02. UPLC-ESI-MS: *m*/*z* calcd for C_18_H_24_NO^+^ [M + H]^+^ 286.18; found: 286.13; t*_R_* = 1.26.

##### Synthesis of 4-(diethylamino)-2-(naphthalen-2-yl)butan-1-ol (**2b**)

Colorless oil. Yield: 67%; Rf = 0.24 (EtOAc/MeOH/Et_3_N 90:10:2.5). ^1^H-NMR (400 MHz, CDCl_3_): δ 1.12 (t, *J* = 7.2 Hz, 6H), 1.95–2.05 (m, 1H), 2.08–2.22 (m, 1H), 2.49–2.70 (m, 4H), 2.73–2.85 (m, 2H), 2.97–3.05 (m, 1H), 3.73–3.83 (m, 2H), 7.37 (d, *J* = 8.5 Hz, 1.8 Hz, 1H), 7.42–7.51 (m, 2H), 7.67 (s, 1H), 7.77–7.86 (m, 3H). ^13^C-NMR (101 MHz, CDCl_3_): δ 10.81, 33.14, 46.30, 49.58, 52.47, 68.14, 125.34, 125.42, 125.97, 126.15, 127.56, 127.59, 128.10, 132.24, 133.57, 141.86. UPLC-ESI-MS: *m*/*z* calcd for C_18_H_26_NO^+^ [M + H]^+^ 272.20; found: 272.15; t*_R_* = 1.41.

##### Synthesis of 4-(benzyl(methyl)amino)-2-(naphthalen-2-yl)butan-1-ol (**2c**)

Colorless oil. Yield: 20%; Rf = 0.43 (EtOAc/MeOH/Et_3_N 90:10:2.5). ^1^H-NMR (400 MHz, CDCl_3_): δ 1.28 (t, *J* = 7.1 Hz, 0.33H), 1.69–1.72 (m, 0.33H), 2.27 (s, 3H), 1.99–2.08 (m, 1H), 2.14–2.25 (m, 1H), 2.26 (s, 3H), 2.46–2.55 (m, 1H), 2.66–2.75 (m, 1H), 3.01–3.09 (m, 1H), 3.54 (d, *J* =12.8 Hz, 1H), 3.70–3.73 (m,0.33H), 3.83 (d, *J* = 6.5 Hz, 2H), 7.28–7.40 (m, 6H), 7.42–7.51 (m, 2H), 7.65 (s, 1H), 7.77–7.86 (m, 3H). ^13^C-NMR (101 MHz, CDCl_3_): δ 32.54, 41.39, 48.89, 56.78, 62.57, 67.93, 125.40, 125.66, 126.00, 126.12, 127.42, 127.57, 128.17, 128.45, 129.41, 132.31, 133.55, 137.42, 141.38. UPLC-ESI-MS: *m*/*z* calcd for C_22_H_26_NO^+^ [M + H]^+^ 320.20; found: 320.09; t*_R_* = 1.73.

##### Synthesis of 2-(naphthalen-2-yl)-4-(piperidin-1-yl)butan-1-ol (**2d**)

Colorless oil. Yield: 64%; Rf = 0.26 (EtOAc/MeOH/Et_3_N 90:10:2.5). ^1^H-NMR (400 MHz, CDCl_3_): δ 1.43–1.55 (m, 2H), 1.60–1.77 (m, 4H), 1.92–2.02 (m, 1H), 2.12–2.24 (m, 1H), 2.31–2.78 (m, 6H), 2.97–3.07 (m, 1H), 3.78 (d, *J* = 6.5 Hz, 2H), 7.37 (dd, *J* = 8.5 Hz, 1.8 Hz, 1H), 7.41–7.51 (m, 2H), 7.66 (s, 1H), 7.77–7.89 (m, 3H). ^13^C-NMR (101 MHz, CDCl_3_): δ 24.21, 25.54, 32.56, 49.74, 54.42, 58.20, 68.13, 125.35, 125.97, 126.17, 127.56, 127.60, 128.10, 132.24, 133.57, 141.90. UPLC-ESI-MS: *m*/*z* calcd for C_19_H_26_NO^+^ [M + H]^+^ 284.20; found: 284.13; t*_R_* = 1.43.

##### Synthesis of 4-(4-benzylpiperidin-1-yl)-2-(naphthalen-2-yl)butan-1-ol (**2e**)

Whitish solid. Yield: 42%; Rf = 0.5 (DCM/MeOH/Et_3_N 95:5:1). ^1^H NMR (400 MHz, CDCl_3_) δ 1.40 (td, *J* = 12.3, 5.9 Hz, 2H), 1.51 (ddp, *J* = 10.9, 7.1, 3.6 Hz, 1H), 1.64 (ddd, *J* = 13.2, 6.7, 3.4 Hz, 2H), 1.76–1.96 (m, 2H), 2.11 (pd, *J* = 11.6, 10.6, 5.8 Hz, 2H), 2.40 (dt, *J* = 12.8, 4.7 Hz, 1H), 2.45–2.58 (m, 3H), 2.93 (dq, *J* = 12.7, 4.3 Hz, 2H), 3.16 (d, *J* = 11.4 Hz, 1H), 3.64–3.75 (m, 2H), 7.02–7.10 (m, 2H), 7.10–7.16 (m, 1H), 7.17–7.30 (m, 4H), 7.32–7.43 (m, 2H), 7.57 (d, *J* = 1.7 Hz, 1H), 7.72 (t, *J* = 7.9 Hz, 3H). ^13^C NMR (100 MHz, CDCl_3_) δ 30.29, 30.47, 36.44, 40.37, 47.75, 53.03, 54.47, 66.27, 126.00, 126.30, 126.36, 126.56, 127.03, 127.62, 127.90, 127.95, 128.42, 129.04, 133.62, 133.67, 139.28, 141.15. UPLC-ESI-MS: *m*/*z* calcd for C_26_H_32_NO^+^ [M + H]^+^ 374.25; found: 374.18; t*_R_* = 1.58.

##### Synthesis of 2-(6-methoxynaphthalen-2-yl)-4-morpholinobutan-1-ol (**3a**)

Colorless oil. Yield: 22%; Rf = 0.33 (EtOAc/MeOH/Et_3_N 90:10:2.5). ^1^H-NMR (400 MHz, CDCl_3_): δ 1.95–199 (m, 1H), 2.10 (m, 1H), 2.47–2.59 (m, 4H), 2.68 (br, 2H), 3.01 (m, 1H), 3.77–3.83 (m, 6H), 3.93 (s, 3H), 7.12–7.16 (m, 2H), 7.32 (dd, *J* = 8.5, 1.8 Hz, 1H), 7.59 (d, *J* = 1.3 Hz, 1H), 7.70 (dd, *J* = 8.6, 3.3 Hz, 2H). ^13^C-NMR (101 MHz, CDCl_3_): δ 31.23, 48.64, 53.55, 55.31, 57.80, 66.58, 66.65, 67.99, 105.59, 118.90, 125.59, 126.49, 127.11, 129.06, 133.39, 138.64, 157.42. UPLC-ESI-MS: m/z calcd for C_19_H_26_NO_3_^+^ [M + H]^+^ 316.19; found: 316.03; t*_R_* = 1.28.

##### Synthesis of 4-(diethylamino)-2-(6-methoxynaphthalen-2-yl)butan-1-ol (**3b**)

Colorless oil. Yield: 51%; Rf = 0.33 (EtOAc/MeOH/Et_3_N 90:10:2.5). ^1^H NMR (400 MHz, CDCl_3_) δ 1.14 (t, *J* = 7.2 Hz, 6H), 2.01 (m, 1H), 2.19 (m, 1H), 2.56–2.74 (m, 4H), 2.83 (m, 2H), 2.99 (m, 1H), 3.70–3.80 (m, 2H), 3.93 (s, 3H), 7.09–7.18 (m, 2H), 7.33 (dd, *J* = 8.4, 1.7 Hz, 1H), 7.60 (d, *J* = 1.3 Hz, 1H), 7.70 (d, *J* = 8.5 Hz, 2H). ^13^C-NMR (101 MHz, CDCl_3_): δ 10.80, 33.09, 46.20, 46.29, 49.33, 52.44, 55.29, 68.22, 105.58, 118.79, 125.28, 126.62, 126.95, 129.07, 133.26, 139.49, 157.32. UPLC-ESI-MS: *m*/*z* calcd for C_19_H_26_NO_2_^+^ [M + H]^+^ 302.21; found: 302.05; t*_R_* = 1.41.

##### Synthesis of 4-(benzyl(methyl)amino)-2-(6-methoxynaphthalen-2-yl)butan-1-ol (**3c**)

Colorless oil. Yield: 13%; Rf = 0.56 (EtOAc/MeOH/Et_3_N 90:10:2.5). ^1^H-NMR (400 MHz, CDCl_3_): δ 1.70 (m, 1H), 2.01 (m, 1H), 2.18 (m, 1H), 2.27 (s, 3H), 2.50 (m, 1H), 2.71 (m, 1H), 3.01 (m, 1H), 3.55 (d, *J* = 12.7Hz, 1H), 3.67–3.72 (m, 2H), 3.93 (s, 3H), 7.12–7.16 (m, 2H), 7.31–7.33 (m, 2H), 7.37 (m, 3H), 7.57 (d, *J* = 1.4Hz, 1H), 7.70 (d, *J* = 8.5Hz, 2H). ^13^C-NMR (101 MHz, CDCl_3_): δ 29.93, 30.96, 32.45, 41.31, 48.66, 55.32, 56.77, 62.46, 62.77, 68.03, 105.55, 118.86, 125.52, 126.60, 127.06, 127.54, 128.51, 129.01, 129.11, 129.50, 133.34, 138.95, 157.37. UPLC-ESI-MS: *m*/*z* calcd for C_23_H_28_NO_2_^+^ [M + H]^+^ 350.21; found: 349.97; t*_R_* = 1.70.

##### Synthesis of 2-(6-methoxynaphthalen-2-yl)-4-(piperidin-1-yl)butan-1-ol (**3d**)

Colorless oil. Yield: 33%; Rf = 0.36 (EtOAc/MeOH/Et_3_N 90:10:2.5). ^1^H-NMR (400 MHz, CDCl_3_): δ 1.25 (m, 1H), 1.52 (br, 2H), 1.73 (br, 4H), 2.01 (m, 1H), 2.19 (m, 1H), 2.51 (m, 2H), 2.61 (m, 1H), 2.66–2.77 (br, 2H), 2.99 (m, 1H), 3.77 (m, 2H), 3.92 (s, 3H), 7.11–7.16 (m, 2H), 7.32 (dd, *J* = 8.4, 1.7 Hz, 1H), 7.58 (d, *J* = 1.2 Hz, 1H), 7.69 (d, *J* = 8.5 Hz, 2H). ^13^C-NMR (101 MHz, CDCl_3_): δ 24.12, 25.40, 32.32, 49.36, 54.35, 55.29, 58.12, 68.17, 105.49, 105.57, 118.80, 125.26, 126.62, 126.96, 129.07, 133.27, 139.39, 157.33. UPLC-ESI-MS: *m*/*z* calcd for C_20_H_28_NO_2_^+^ [M + H]^+^ 314.21; found: 314.03; t*_R_* = 1.44.

##### Synthesis of 4-(4-benzylpiperidin-1-yl)-2-(6-methoxynaphthalen-2-yl)butan-1-ol (**3e**)

Whitish solid. Yield: 26%; Rf = 0.45 (DCM/MeOH/Et_3_N 95:5:1). ^1^H NMR (400 MHz, CDCl_3_) δ 1.51 (m, 3H), 1.65 (m, 2H), 1.95 (m, 2H), 2.16 (m, 2H), 2.40–2.63 (m, 3H), 2.86–3.11 (m, 2H), 3.20 (m, 1H), 3.59–3.78 (m, 2H), 3.84 (s, 3H), 6.99–7.09 (m, 4H), 7.13 (t, *J* = 7.3 Hz, 1H), 7.17–7.28 (m, 5H), 7.50 (d, *J* = 1.8 Hz, 1H), 7.61 (d, *J* = 8.6 Hz, 2H). ^13^C NMR (100 MHz, CDCl_3_) δ 30.20, 30.47, 36.31, 40.63, 47.69, 53.36, 54.55, 55.26, 66.27, 106.20, 118.39, 125.95, 126.29, 126.34, 127.57, 128.33, 129.04, 129.32, 129.70, 133.47, 139.05, 141.12, 157.51. UPLC-ESI-MS: *m*/*z* calcd for C_27_H_34_NO_2_^+^ [M + H]^+^ 404.26; found: 404.15; t*_R_* = 1.53.

### 4.2. Receptor Binding Studies

The following are the materials employed for the receptor binding studies. Guinea pig brains are commercially available (Harlan-Winkelmann, Borchen, Germany). Homogenizers: Elvehjem Potter (B. Braun Biotech International, Melsungen, Germany) and Soniprep^®^ 150 (MSE, London, UK). Centrifuges: cooling centrifuge model Eppendorf 5427R (Eppendorf, Hamburg, Germany) and high-speed cooling centrifuge model Sorvall^®^ RC-5C plus (Thermo Fisher Scientific, Langenselbold, Germany). Multiplates: standard 96-well multiplates (Diagonal, Muenster, Germany). Shaker: self-made device with adjustable temperature and tumbling speed (scientific workshop of the institute). Harvester: MicroBeta^®^ FilterMate 96 Harvester. Filter: Printed Filtermat Typ A and B. Scintillator: Meltilex^®^ (Typ A or B) solid state scintillator. Scintillation analyzer: MicroBeta^®^ Trilux (all Perkin Elmer LAS, Rodgau-Jügesheim, Germany).

#### 4.2.1. Preparation of Membrane Homogenates from Guinea Pig Brain 

Five guinea pig brains were homogenized with the potter (500–800 rpm, 10 up and down strokes) in 6 volumes of cold 0.32 M sucrose. The suspension was centrifuged at 1200× *g* for 10 min at 4 °C. The supernatant was separated and centrifuged at 23,500× *g* for 20 min at 4 °C. The pellet was resuspended in 56 volumes of buffer (50 mM TRIS, pH 7.4) and centrifuged again at 23,500× *g* (20 min, 4 °C). This procedure was repeated twice. The final pellet was resuspended in 56 volumes of buffer and frozen (80 °C) in 1.5 mL portions containing about 1.5 mg protein/mL. 

#### 4.2.2. S1R Binding Assay Protocol 

The test compound solutions were prepared by dissolving approximately 10 µmol (usually 2–4 mg) of test compound in dimethyl sulfoxide (DMSO, unless otherwise specified), so that a 10 mM stock solution was obtained. To obtain the required test solutions for the assay, the DMSO stock solution was diluted with the respective assay buffer. The filtermats were presoaked in 0.5% aqueous polyethylenamine solution for 2 h at r.t. before use. All the binding experiments were carried out in duplicate in 96-well multiplates. The concentrations given are the final concentrations in the assay. Generally, the assays were performed by the addition of 50 µL of the respective assay buffer, 50 µL of test compound solution at various concentrations (10^- 5^, 10^−6^, 10^−7^, 10^−8^, 10^−9^, and 10^−10^ M), 50 µL of corresponding radioligand solution, and 50 µL of the respective receptor preparation into each well of the multiplate (total volume 200 µL). The receptor preparation was always added last. During the incubation, the multiplates were shaken at a speed of 500–600 rpm at the specified temperature. The assays were terminated after 120 min by rapid filtration using the harvester. During the filtration each well was washed five times with 300 µL of water. Subsequently, the filtermats were dried at 95 °C. The solid scintillator was melted on the dried filtermats at 95 °C for 5 min. After the solidifying of the scintillator at r.t., the trapped radioactivity in the filtermats was measured with the scintillation analyzer. Each position on the filtermat corresponding to one well of the multiplate was measured for 5 min with the [^3^H]-counting protocol. The overall counting efficiency was 20%. The IC_50_ values were calculated with GraphPad Prism 3.0 (GraphPad Software, San Diego, CA, USA) by nonlinear regression analysis. The IC_50_ values were subsequently transformed into *K*_i_ values using the equation of Cheng and Prusoff. All the experiments were carried out in duplicates. Compounds with high S1R affinity were tested in three independent experiments, and their *K*_i_ values are given as mean value ± SEM from the three measurements. The assay was performed with the radioligand [^3^H](+)-pentazocine (22.0 Ci/mmol; Perkin-Elmer). The thawed membrane preparation of guinea pig brain (≈100 µg protein) was incubated with various concentrations of test compounds, 2 nM [^3^H](+)-pentazocine, and TRIS buffer (50 mM, pH 7.4) at 37 °C. The nonspecific binding was determined with 10 µM unlabeled (+)-pentazocine. The *K*_d_ value of (+)-pentazocine was 2.9 nM. 

### 4.3. NGF-Induced Neurite Outgrowth Assay in PC12 Cells

#### 4.3.1. Cell Culture

The PC12 cells were cultured (37 °C; 5% CO_2_) in proliferation medium (PM) containing RPMI-1640 (Carlo Erba, Cornaredo, Italy) supplemented with 10% heat inactivated horse serum (HS) (Gibco, Thermo Fisher Scientific, Segrate, Italy), 5% fetal bovine serum (FBS) (GE Healthcare HyClone, Milan, Italy), 1% GlutaMAX Supplement (Gibco, Thermo Fisher Scientific, Segrate, Italy), and 1% Penicillin/Streptomycin 10.000 U/mL (Gibco). At 80% confluence, the cells were either split to allow a further culture expansion or plated onto 48-well flat-bottom plates previously coated with poly-L-ornithine (Sigma Aldrich, Milan, Italy) at the final concentration of 10 μg/cm^2^. The cells were plated at low density (2500 cells/well) to allow proper neurite elongation. The PC12 cells were mechanically detached from the flask to avoid cell-surface receptor cleavage and to preserve the correct binding of NGF to the TrkA receptors. To minimize detachment stress, the cells were left in PM for 24 h to recover. Then, the PM was replaced by differentiation medium (DM) containing RPMI-1640 supplemented with 0.5% HS, 1% GlutaMAX, 1% Penicillin/Streptomycin, 2.5 ng/mL of NGF, and the S1R ligands (*R*)-RC-33, compounds 2 and 7. Each compound was dissolved in DMSO to obtain a 10mM stock solution, and then, it was further diluted in ultra-pure and sterile water up to the final concentration: 0.25 µM and 2.5 µM. PRE-084 (10 µM), an S1R agonist, was used to verify that the effects of the selected compounds were due to the S1R modulation. In CTR conditions, the cells were incubated with the drug vehicle. For all the ligands, two replicates per concentration were performed in at least 3 experiments with different PC12 batches. 

#### 4.3.2. Quantification of Neurite Outgrowth 

After 72 h incubation in DM, the cells were fixed with a 4% paraformaldehyde solution (pH 7.4). To allow the neurite visualization with a bright-field microscope, the cells were stained with the Harris Hematoxylin solution (Sigma Aldrich). After staining, images of six fields per well were acquired with a digital camera linked to a bright-field inverted microscope (Optika) (20× magnification). Morphometric analysis was performed using ImageJ software (free software released by NIH, https://imagej.nih.gov/ij, accessed on 30 May 2023). Only the cells bearing a neurite as long as their soma’s diameter were identified as differentiated cells. The number of differentiated cells was expressed as the percentage of differentiated cells over the total number of cells.

### 4.4. Water Permeability Assay

Osmotic water permeability was measured in the HeLa cell suspension by the stopped-flow light scattering method, as previously described [65]. The experiments were performed at 25 °C on the stopped-flow apparatus (RX2000, Applied Photophysics, Leatherhead, UK) with a pneumatic drive accessory (DA.1, Applied Photophysics) straightforwardly coupled with a Varian Cary 50 spectrometer (Varian Australia Pty Ltd., Mulgrave, Australia). Briefly, scattered light intensity with a dead time of 6 ms was recorded at a wavelength of 450 nm. The time course of the cell swelling caused by exposure to the hypotonic gradient (150 mosm/L) was measured for 60 s at the acquisition rate of one point/0.0125 s. The initial rate constant of the cell volume changes (k) was obtained by setting the time course light scattering with a single exponential equation (GraphPad Prism 4.00, 2003). To evaluate the effect of the test compounds on water permeability, the HeLa cells were divided into different groups: (1) controls, cells incubated at r.t. (21 °C) in the presence of the same volume of methanol as that of the treated cells; (2) heat-stressed cells, cells heat-treated in a water thermostatic and shacking bath at 42 °C for 3 h; (3) heat-stressed cells pre-treated with the test compounds at a 20 µM final concentration (dissolved in methanol). 

### 4.5. In Vivo Toxicity

#### 4.5.1. Animal Care and Maintenance 

The Tg(GFAP:GFP) zebrafish (*Danio rerio*) were a kind gift from the Department of Biology, University of Padova (Italy), and were maintained in a re-circulatory system at 28 °C in a light/dark cycle of 14/10 h. They were fed three times a day with dry food and Artemia. The zebrafish embryos and larvae were used for the experiments until 5 days post-fertilization (dpf); mating between 2 females and 1 male was set up the day before the test. The eggs were preserved from predation by adult fish using a plastic separator in the tanks. The eggs were collected right after spawning and used in the teratogenicity experiments. The zebrafish husbandry procedures were performed according to the Directive 2010/63/EU and in compliance with the local animal welfare regulations (authorization n. prot. 18,311/2016; released by the “Comune di Meldola”, 9 November 2016). 

#### 4.5.2. Toxicity Evaluation 

The toxicity evaluation was performed as previously reported [73]. Briefly, the eggs were collected, rinsed out with embryo medium (for 1 L of distilled water: 0.1 g sodium bicarbonate, 0.1 g instant ocean, 0.19 g calcium sulphate) and put in different Petri dishes with the same medium; only the unscathed embryos were selected. Generally, 8 embryos per untreated control and treatment concentrations were cultured. The eggs were treated with an increasing concentration of (R/S)-**1c** and (R/S)-**1d**, starting from 1 h post-fertilization. They were incubated at 28 °C for about 5 days (without changing the media) in order to allow exposure to the compounds throughout all development stages. The teratogenic effects were evaluated after 5 days from treatment with morphological and viability observations. The experiment was performed in octuplicate, and the data were assessed using a sample score sheet, as reported by Brannen KC and colleagues [74].

### 4.6. In Vitro Metabolic Stability

Mouse liver microsomes (Sigma Aldrich, CD-1 male, pooled) at 0.5 mg/mL were preincubated with the test compound (*R*/*S*)-**1d** dissolved in DMSO at 1 μM in phosphate buffer 50 mM, pH 7.4, and 3 mM MgCl2 for 10 min at 37 °C [75]. The reaction was then started by adding the cofactor mixture solution (NADP, glucose-6-phosphate, glucose-6-phosphate dehydrogenase in 2% NaHCO3). The samples were taken at 0, 10, 30, 45, and 60 min and added to acetonitrile to stop the reaction (n = 3). The samples were then centrifuged, and the supernatant was analyzed by LC-MS/MS to quantify the amount of compound. A control sample without cofactor was always added to check the chemical stability of the test compound. Two reference compounds of known metabolic stability, 7-ethoxycoumarin (7-EC) and propranolol, were present in parallel testing. A fixed concentration of verapamil was added to every sample as an internal standard for LC-MS/MS analyses. The percentage of the area of the test compound remaining at the various incubation times was calculated with respect to the area of the compound at time 0 min.

The intrinsic clearance (Cli) was calculated by the equation:

Cli (μL/min/mg) = k/microsomal conc. × 1000

where k is the rate constant (min^−1^) and the microsomal protein conc. = 0.5 mg protein/mL. The rate constant k (min^−1^), derived for the exponential decay equation (peak area/IS vs. time), was used to calculate the rate of Cli. The classification of the in vitro stability is presented in Table 2.

#### LC–MS/MS Analytical Method

The samples were analyzed under the following conditions: UFLC LC-20 Shimadzu coupled with an API 3200 triple-quadrupole (ABSciex); eluents, (phase A) water + 0.1% trifluoroacetic acid (TFA), (phase B) CH_3_CN + 0.1% TFA; flow rate, 0.3 mL/min; column, Gemini-Nx 5 μm C18 110A (50 × 2.00 mm) at 35 °C; and injection volume, 5 μL. LC–MS/MS analyses were carried out using an ESI(+) interface in a multiple reaction monitoring mode (MRM). The conditions and MRM transitions applied to the compounds are described in Table 3. The source conditions were ESI positive: T 400 °C, Gas 1 30, Gas 2 35, CUR 20, IS 5500, and CAD 5. 

### 4.7. MTS Cytotoxicity Assay

#### Cell Proliferation Assay

A CellTiter 96^®^ AQueous One Solution Cell Proliferation Assay (Promega, Milan, Italy) was used on cells seeded onto a 96-well plate at a density of 9 × 10^3^ cells per well. The effect of the test compound was evaluated at four different concentrations (0.1, 1, 10, and 100 μM) after 24 h of continued exposure. Two independent experiments were performed for each concentration. The optical density (OD) of the treated and untreated cells was determined at a wavelength of 490 nm using a plate reader. The data were elaborated with the GraphPad Prism 5 program.

*Statistical analysis.* All statistical analyses were performed using the standard software package GraphPad Prism (GraphPad Software, San Diego, CA, USA). Different tests were applied according to different datasets, as specified in each image’s caption.

### 4.8. In Vivo Assays

#### 4.8.1. Animals

ICR male mice weighing 20–25 g (5 weeks old) and SD rats weighing 200–250 g (7 weeks old) were used in the experiments. All the animals were purchased from SAMTACO (Osan-si, Korea). Six mice/group and eight rats/group were used for this study. Four groups of mice were utilized for the assessment of the antinociceptive activity in the formalin test (one vehicle and three doses; 1, 10, and 20 mg/kg given i.p.), while five groups of rats were used for the assessment of the antinociceptive activity in Chung’s neuropathic pain model (one vehicle and four doses; 5, 10, 25 and 50 mg/kg given i.p.). The ICR mice and SD rats were maintained on a 12 hr light–dark cycle (light on between 7:00 p.m. and 7:00 a.m.) and allowed free access to food and water. The temperature and humidity of the animal room were maintained at 22 ± 2 °C and 50 ± 5%, respectively. In this study, we followed the national guidelines for conducting animal experiments. All the procedures for the animal tests were approved by the Medifron Animal Care and Use Committee (approval number, Medifron 2014-8, IACUC). All efforts were made to minimize animal suffering and reduce the number of animals used.

#### 4.8.2. Formalin-Induced Licking Paw Test

The formalin-induced licking paw test was modified from the method described by Dubuisson and Dennis [79]. Each mouse was acclimated to an acrylic observation chamber for at least 5 min before the injection of formalin. Twenty microliters of 2% formalin was injected subcutaneously into right side of the hind paw. Each mouse was then placed in an individual clear plastic observational chamber (15 × 15 × 15), and the pain response was recorded for a period of 30 min. The summation of time (in seconds) spent in licking and biting responses of the injected paw during each 5 min block was measured as an indicator of the pain response. The first period (early phase) was recorded 0–5 min after the injection of formalin, and the second period (late phase) was recorded 20–30 min after the injection. The test compound was administered intraperitoneally 30 min before the formalin injection at three different doses: 1, 10, and 20 mg/Kg. The vehicle was DMSO/Cremophor EL/D.W. (10/10/80). The data were expressed as mean values (SEM). Statistical analyses were performed through two-way repeated measures ANOVA followed by a post hoc Bonferroni test.

#### 4.8.3. Sciatic Nerve Ligation Model

Ligation of the left L5 and L6 spinal nerves in the rats was used as an experimental model of neuropathic pain. The rats were anesthetized by inhalation of 4% isoflurane in 95% of O_2_, and anesthesia was maintained throughout the surgery. The surgical procedure was performed according to the method described by Kim and Chung [80]. The left L5 spinal nerve was isolated and ligated tightly with 6–0 black silk. The wound was closed in anatomical layers, with the skin being closed with stainless steel wound clips. The animals were then allowed to recover after the surgery. The behavioral signs representing neuropathic pain (mechanical allodynia) were examined in all the rats for 2 weeks postoperatively. The mechanical withdrawal threshold to the application of a von Frey filament (Stoelting, Wood Dale, IL, USA) was measured by using the up–down method [81].The most sensitive area was first determined by poking various areas of the paw with a von Frey hair. Next, the actual test was conducted by gently poking various areas of the spot with the filament. A von Frey filament was applied 10 times (once every 3–4 sec) to each hind paw. The forces of the von Frey filaments ranged from 0.8 to 15 g. The frequency of foot withdrawal expressed as a percentage was used as the index of mechanical allodynia. The animals received the vehicle or drug by intraperitoneal injection. The vehicle was DMSO/Cremophor EL/D.W. (10/10/80). The test compound was administered at four different doses: 5, 10, 25, and 50 mg/Kg. The measurements to assess mechanical allodynia were taken at 30 min, 60 min and 90 min after dosing. The results were calculated using the one-way analysis of variance (ANOVA) followed by Bonferroni’s post hoc test. The results are presented as the means ± SEM.

### 4.9. Statistical Analysis

The results from the PC12 differentiation assay were reported as mean ± SEM of three different experiments and were evaluated using ANOVA followed by Dunnett’s post hoc test. The results from the water permeability assay were expressed as means ± SEM of 4–15 single shots (time course curves) for each of the 4–6 different experiments and were analyzed by ANOVA, followed by the Newman–Keuls Q test. The results from the in vivo toxicity in the zebrafish model were reported as the mean of the values assessed using a sample score sheet, as reported by Brannen KC and colleagues. The results from the formalin assay were expressed as the means ± SEM of 8 mice per group and were analyzed by two-way ANOVA followed by Bonferroni’s post hoc test. Lastly, the results from Chung’s neuropathic pain model were reported as means ± SEM and evaluated using one-way ANOVA followed by Bonferroni’s post hoc test. The data were considered to be statistically significant if *p* < 0.05 (*), *p* < 0.01 (**), *p* < 0.001 (***), and *p* < 0.0001 (****). Statistical analysis was performed using GraphPad Prism Software (version 9.3).

## Figures and Tables

**Table 1:** Chemical structures and binding affinities and selectivity of compounds **1a**–**3e** toward S1R and S2R.

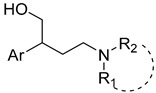						
Cmpd	Ar	R_1_	R_2_	S1R *K*_i_ (nM)	S2R *K*_i_ (nM)	Selectivity *(K*_i_ S2R/*K*_i_ S1R)
**1a**	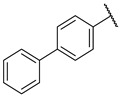	-CH_2_CH_2_OCH_2_CH_2_-		13 ± 3	310	24
**1b**		CH_3_CH_2_-	CH_3_CH_2_-	20 ± 4	677	34
**1c**		CH_3_-	-Bn	0.95 ± 0.08	121	127
**1d**		-CH_2_CH_2_CH_2_CH_2_CH_2_-		6.2 ± 0.9	360	58
**1e**		-CH_2_CH_2_CH(CH_2_Ph)CH_2_CH_2_-		94 ± 12	197	2
**2a**	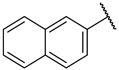	-CH_2_CH_2_OCH_2_CH_2_-		206 ± 37	6300	31
**2b**		CH_3_CH_2_-	CH_3_CH_2_-	110 ± 25	1100	10
**2c**		CH_3_-	-Bn	9.5 ± 1.8	35 ± 3	4
**2d**		-CH_2_CH_2_CH_2_CH_2_CH_2_-		15 ± 6	167	11
**2e**		-CH_2_CH_2_CH(CH_2_Ph)CH_2_CH_2_-		25 ± 4	331	13
**3a**	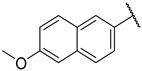	-CH_2_CH_2_OCH_2_CH_2_-		324 ± 10	1300	4
**3b**		CH_3_CH_2_-	CH_3_CH_2_-	87 ± 28	1200	14
**3c**		CH_3_-	-Bn	20 ± 3	138	7
**3d**		-CH_2_CH_2_CH_2_CH_2_CH_2_-		38 ± 9	1200	32
**3e**		-CH_2_CH_2_CH(CH_2_Ph)CH_2_CH_2_-		85 ± 11	53	0.6

**Table 2:** In vitro clearance classification *^a^*.

Cli (µL/min/mg Protein)			
Classification	Low Cli	Medium Cli	High Cli
Mouse	≤2.5	2.5–66	>66

*^a^* Data obtained from Refs. [76,77,78].

**Table 3:** Compounds, MRM transitions, and conditions.

Compound	Parent Ion	Product Ion	DP (V)	CE (eV)
**7-EC**	190.9	163.0	56	23
**Propranolol**	260.4	183.2	40	25
**Verapamil**	455.4	165.1	31	35
**(R/S)-1d**	310.4	179.3	66	45

## Data Availability

The data presented in this study are available within the article and supplementary materials.

## Supplementary Materials

The following supporting information can be downloaded at: https://www.mdpi.com/article/10.3390/ph16070962/s1, Table S1: Physicochemical properties for compounds **1c** and **1d** computed by SwissADME; Table S2: Sample score sheet; Figure S1: Cytotoxicity evaluation (expressed as % of cell survival) for compounds (*R*/*S*)-**1c** and (*R*/*S*)-**1d** against CHME-5 and MRC-5 cell lines; Figure S2: Kinetic stability profile of (R/S)-**1d** in mouse liver microsomes; ^1^H-NMR spectra for compound **1a**–**3e**; Representative UPLC-MS traces of compounds **1a**, **1c**,**1d** and **2d**.

## References

[1] Finnerup N.B., Kuner R., Jensen T.S. (2021). Neuropathic Pain: From Mechanisms to Treatment. Physiol. Rev..

[2] Alles S.R.A., Smith P.A. (2018). Etiology and Pharmacology of Neuropathic Pain. Pharmacol. Rev..

[3] Gierthmühlen J., Baron R. (2016). Neuropathic Pain. Semin. Neurol..

[4] Baron R., Binder A., Wasner G. (2010). Neuropathic Pain: Diagnosis, Pathophysiological Mechanisms, and Treatment. Lancet Neurol..

[5] Attal N., Martinez V., Bouhassira D. (2021). Potential for Increased Prevalence of Neuropathic Pain after the COVID-19 Pandemic. Pain Rep..

[6] Magdy R., Eid R.A., Fathy W., Abdel-Aziz M.M., Ibrahim R.E., Yehia A., Sheemy M.S., Hussein M. (2022). Characteristics and Risk Factors of Persistent Neuropathic Pain in Recovered COVID-19 Patients. Pain Med..

[7] McFarland A.J., Yousuf M.S., Shiers S., Price T.J. (2021). Neurobiology of SARS-CoV-2 Interactions with the Peripheral Nervous System: Implications for COVID-19 and Pain. Pain Rep..

[8] Ellul M.A., Benjamin L., Singh B., Lant S., Michael B.D., Easton A., Kneen R., Defres S., Sejvar J., Solomon T. (2020). Neurological Associations of COVID-19. Lancet Neurol..

[9] Bouhassira D. (2019). Neuropathic Pain: Definition, Assessment and Epidemiology. Rev. Neurol..

[10] Finnerup N.B., Attal N., Haroutounian S., McNicol E., Baron R., Dworkin R.H., Gilron I., Haanpää M., Hansson P., Jensen T.S. (2015). Pharmacotherapy for Neuropathic Pain in Adults: A Systematic Review and Meta-Analysis. Lancet Neurol..

[11] Humo M., Lu H., Yalcin I. (2019). The Molecular Neurobiology of Chronic Pain–Induced Depression. Cell Tissue Res..

[12] Cherif F., Zouari H.G., Cherif W., Hadded M., Cheour M., Damak R. (2020). Depression Prevalence in Neuropathic Pain and Its Impact on the Quality of Life. Pain Res. Manag..

[13] Mathieson S., Lin C.-W.C., Underwood M., Eldabe S. (2020). Pregabalin and Gabapentin for Pain. BMJ.

[14] Senderovich H., Jeyapragasan G. (2018). Is There a Role for Combined Use of Gabapentin and Pregabalin in Pain Control? Too Good to Be True?. Curr. Med. Res. Opin..

[15] Duehmke R.M., Derry S., Wiffen P.J., Bell R.F., Aldington D., Moore R.A. (2017). Tramadol for Neuropathic Pain in Adults. Cochrane Database Syst. Rev..

[16] Freo U., Romualdi P., Kress H.G. (2019). Tapentadol for Neuropathic Pain: A Review of Clinical Studies. J. Pain Res..

[17] Cooper T., Chen J., Wiffen J., Derry S., Carr B., Aldington D., Cole P., Moore R. (2017). Morphine for Chronic Neuropathic Pain in Adults. Cochrane Database Syst. Rev..

[18] Insights Q.M. Non-Opioid Pain Treatment Market Size to Grow at a CAGR of 16.8% from 2021 to 2030. https://www.grandviewresearch.com/industry-analysis/non-opioid-pain-treatment-market-report.

[19] Linciano P., Rossino G., Listro R., Rossi D., Collina S. (2020). Sigma-1 Receptor Antagonists: Promising Players in Fighting Neuropathic Pain. Pharm. Pat. Anal..

[20] Ruiz-Cantero M.C., González-Cano R., Tejada M.Á., Santos-Caballero M., Perazzoli G., Nieto F.R., Cobos E.J. (2021). Sigma-1 Receptor: A Drug Target for the Modulation of Neuroimmune and Neuroglial Interactions during Chronic Pain. Pharmacol. Res..

[21] Díaz J.L., Zamanillo D., Corbera J., Baeyens J.M., Maldonado R., Pericàs M.A., Vela J.M., Torrens A. (2009). Selective Sigma-1 (Sigma1) Receptor Antagonists: Emerging Target for the Treatment of Neuropathic Pain. Cent. Nerv. Syst. Agents Med. Chem..

[22] Merlos M., Romero L., Zamanillo D., Plata-Salamán C., Vela J.M. (2017). Sigma-1 Receptor and Pain. Handb. Exp. Pharmacol..

[23] Bravo-Caparros I., Ruiz-Cantero M.C., Perazzoli G., Cronin S.J.F., Vela J.M., Hamed M.F., Penninger J.M., Baeyens J.M., Cobos E.J., Nieto F.R. (2020). Sigma-1 Receptors Control Neuropathic Pain and Macrophage Infiltration into the Dorsal Root Ganglion after Peripheral Nerve Injury. FASEB J. Off. Publ. Fed. Am. Soc. Exp. Biol..

[24] de la Puente B., Zamanillo D., Romero L., Carceller A., Vela J.M., Merlos M., Portillo-Salido E. (2022). Comprehensive Preclinical Assessment of Sensory, Functional, Motivational-Affective, and Neurochemical Outcomes in Neuropathic Pain: The Case of the Sigma-1 Receptor. ACS Pharmacol. Transl. Sci..

[25] Schmidt H.R., Kruse A.C. (2019). The Molecular Function of σ Receptors: Past, Present, and Future. Trends Pharmacol. Sci..

[26] Morales-Lázaro S.L., González-Ramírez R., Rosenbaum T. (2019). Molecular Interplay Between the Sigma-1 Receptor, Steroids, and Ion Channels. Front. Pharmacol..

[27] Carlsson A., Ohsawa M., Hallberg M., Nyberg F., Kamei J. (2010). Substance P(1-7) Induces Antihyperalgesia in Diabetic Mice through a Mechanism Involving the Naloxone-Sensitive Sigma Receptors. Eur. J. Pharmacol..

[28] Cendan C.M., Pujalte J.M., Portillo-Salido E., Montoliu L., Baeyens J.M. (2005). Formalin-Induced Pain Is Reduced in Sigma(1) Receptor Knockout Mice. Eur. J. Pharmacol..

[29] Entrena J.M., Sánchez-Fernández C., Nieto F.R., González-Cano R., Yeste S., Cobos E.J., Baeyens J.M. (2016). Sigma-1 Receptor Agonism Promotes Mechanical Allodynia After Priming the Nociceptive System with Capsaicin. Sci. Rep..

[30] Bravo-Caparrós I., Perazzoli G., Yeste S., Cikes D., Baeyens J.M., Cobos E.J., Nieto F.R. (2019). Sigma-1 Receptor Inhibition Reduces Neuropathic Pain Induced by Partial Sciatic Nerve Transection in Mice by Opioid-Dependent and -Independent Mechanisms. Front. Pharmacol..

[31] Gris G., Cobos E.J., Zamanillo D., Portillo-Salido E. (2015). Sigma-1 Receptor and Inflammatory Pain. Inflamm. Res..

[32] Wilson L., Eans S., Ramadan-Siraj I., Modica M., Romeo G., Intagliata S., McLaughlin J. (2022). Examination of the Novel Sigma-1 Receptor Antagonist, SI 1/28, for Antinociceptive and Anti-Allodynic Efficacy against Multiple Types of Nociception with Fewer Liabilities of Use. Int. J. Mol. Sci..

[33] Nieto F.R., Cendan C.M., Sanchez-Fernandez C., Cobos E.J., Entrena J.M., Tejada M.A., Zamanillo D., Vela J.M., Baeyens J.M. (2012). Role of Sigma-1 Receptors in Paclitaxel-Induced Neuropathic Pain in Mice. J. Pain Off. J. Am. Pain Soc..

[34] Cavaletti G., Alberti P., Argyriou A.A., Lustberg M., Staff N.P., Tamburin S., on behalf of the Toxic Neuropathy Consortium of the Peripheral Nerve Society (2019). Chemotherapy-Induced Peripheral Neurotoxicity: A Multifaceted, Still Unsolved Issue. J. Peripher. Nerv. Syst..

[35] Argyriou A.A., Bruna J., Marmiroli P., Cavaletti G. (2012). Chemotherapy-Induced Peripheral Neurotoxicity (CIPN): An Update. Crit. Rev. Oncol. /Hematol..

[36] Peng Y., Zhang Q., Welsh W.J. (2021). Novel Sigma 1 Receptor Antagonists as Potential Therapeutics for Pain Management. J. Med. Chem..

[37] Tesei A., Cortesi M., Pignatta S., Arienti C., Dondio G.M., Bigogno C., Malacrida A., Miloso M., Meregalli C., Chiorazzi A. (2019). Anti-Tumor Efficacy Assessment of the Sigma Receptor Pan Modulator RC-106. A Promising Therapeutic Tool for Pancreatic Cancer. Front. Pharmacol..

[38] Cortesi M., Zamagni A., Pignatta S., Zanoni M., Arienti C., Rossi D., Collina S., Tesei A. (2020). Pan-Sigma Receptor Modulator RC-106 Induces Terminal Unfolded Protein Response In In Vitro Pancreatic Cancer Model. Int. J. Mol. Sci..

[39] Abatematteo F.S., Niso M., Lacivita E., Abate C. (2021). Σ2 Receptor and Its Role in Cancer with Focus on a MultiTarget Directed Ligand (MTDL) Approach. Molecules.

[40] James M.L., Shen B., Zavaleta C.L., Nielsen C.H., Mesangeau C., Vuppala P.K., Chan C., Avery B.A., Fishback J.A., Matsumoto R.R. (2012). New Positron Emission Tomography (PET) Radioligand for Imaging σ-1 Receptors in Living Subjects. J. Med. Chem..

[41] Díaz J.L., Cuberes R., Berrocal J., Contijoch M., Christmann U., Fernández A., Port A., Holenz J., Buschmann H., Laggner C. (2012). Synthesis and Biological Evaluation of the 1-Arylpyrazole Class of Σ1 Receptor Antagonists: Identification of 4-{2-[5-Methyl-1- (Naphthalen-2-Yl)-1H-Pyrazol-3-Yloxy]Ethyl}morpholine (S1RA, E-52862). J. Med. Chem..

[42] AdisInsight Drugs E-52862. https://adisinsight.springer.com/drugs/800031669.

[43] clinicaltrials.gov. [18F]FTC-146 PET/MRI in Healthy Volunteers and in CRPS and Sciatica. https://clinicaltrials.gov/ct2/show/NCT02753101.

[44] James M.L., Shen B., Nielsen C.H., Behera D., Buckmaster C.L., Mesangeau C., Zavaleta C., Vuppala P.K., Jamalapuram S., Avery B.A. (2014). Evaluation of σ-1 Receptor Radioligand 18 F-FTC-146 in Rats and Squirrel Monkeys Using PET. J. Nucl. Med..

[45] Shen B., Park J.H., Hjørnevik T., Cipriano P.W., Yoon D., Gulaka P.K., Holly D., Behera D., Avery B.A., Gambhir S.S. (2017). Radiosynthesis and First-In-Human PET/MRI Evaluation with Clinical-Grade [18F]FTC-146. Mol. Imaging Biol..

[46] Bruna J., Videla S., Argyriou A.A., Velasco R., Villoria J., Santos C., Nadal C., Cavaletti G., Alberti P., Briani C. (2018). Efficacy of a Novel Sigma-1 Receptor Antagonist for Oxaliplatin-Induced Neuropathy: A Randomized, Double-Blind, Placebo-Controlled Phase IIa Clinical Trial. Neurother. J. Am. Soc. Exp. NeuroTherapeutics.

[47] Weickhardt A., Wells K., Messersmith W. (2011). Oxaliplatin-Induced Neuropathy in Colorectal Cancer. J. Oncol..

[48] Gris G., Portillo-Salido E., Aubel B., Darbaky Y., Deseure K., Vela J.M., Merlos M., Zamanillo D. (2016). The Selective Sigma-1 Receptor Antagonist E-52862 Attenuates Neuropathic Pain of Different Aetiology in Rats. Sci. Rep..

[49] EU Clinical Trials Register. Clinical Trials for 2012-000398-21. https://www.clinicaltrialsregister.eu/ctr-search/search?query=2012-000398-21.

[50] Rossino G., Rui M., Pozzetti L., Schepmann D., Wünsch B., Zampieri D., Pellavio G., Laforenza U., Rinaldi S., Colombo G. (2020). Setup and Validation of a Reliable Docking Protocol for the Development of Neuroprotective Agents by Targeting the Sigma-1 Receptor (S1R). Int. J. Mol. Sci..

[51] Rui M., Rossino G., Coniglio S., Monteleone S., Scuteri A., Malacrida A., Rossi D., Catenacci L., Sorrenti M., Paolillo M. (2018). Identification of Dual Sigma1 Receptor Modulators/Acetylcholinesterase Inhibitors with Antioxidant and Neurotrophic Properties, as Neuroprotective Agents. Eur. J. Med. Chem..

[52] Rui M., Rossi D., Marra A., Paolillo M., Schinelli S., Curti D., Tesei A., Cortesi M., Zamagni A., Laurini E. (2016). Synthesis and Biological Evaluation of New Aryl-Alkyl(Alkenyl)-4-Benzylpiperidines, Novel Sigma Receptor (SR) Modulators, as Potential Anticancer-Agents. Eur. J. Med. Chem..

[53] Listro R., Stotani S., Rossino G., Rui M., Malacrida A., Cavaletti G., Cortesi M., Arienti C., Tesei A., Rossi D. (2020). Exploring the RC-106 Chemical Space: Design and Synthesis of Novel (E)-1-(3-Arylbut-2-En-1-Yl)-4-(Substituted) Piperazine Derivatives as Potential Anticancer Agents. Front. Chem..

[54] Pascual R., Almansa C., Plata-Salamán C., Vela J.M. (2019). A New Pharmacophore Model for the Design of Sigma-1 Ligands Validated on a Large Experimental Dataset. Front. Pharmacol..

[55] Lagorce D., Sperandio O., Galons H., Miteva M.A., Villoutreix B.O. (2008). FAF-Drugs2: Free ADME/Tox Filtering Tool to Assist Drug Discovery and Chemical Biology Projects. BMC Bioinform..

[56] Jagtap S. (2017). Heck Reaction—State of the Art. Catalysts.

[57] Loddo G., Azzolina O., Magnani A., Urbano M., Collina S. (2006). Efficient Microwave and Phosphane-Free Synthesis of Trisubstituted Olefins via Heck Coupling. Lett. Org. Chem..

[58] Rossi D., Rui M., Di Giacomo M., Schepmann D., Wünsch B., Monteleone S., Liedl K.R., Collina S. (2017). Gaining in Pan-Affinity towards Sigma 1 and Sigma 2 Receptors. SAR Studies on Arylalkylamines. Bioorg. Med. Chem..

[59] Ishima T., Hashimoto K. (2012). Potentiation of Nerve Growth Factor-Induced Neurite Outgrowth in PC12 Cells by Ifenprodil: The Role of Sigma-1 and IP3 Receptors. PLoS ONE.

[60] Takebayashi M., Hayashi T., Su T.-P. (2002). Nerve Growth Factor-Induced Neurite Sprouting in PC12 Cells Involves ς-1 Receptors: Implications for Antidepressants. J. Pharmacol. Exp. Ther..

[61] Rossi D., Pedrali A., Urbano M., Gaggeri R., Serra M., Fernández L., Fernández M., Caballero J., Ronsisvalle S., Prezzavento O. (2011). Identification of a Potent and Selective Σ1 Receptor Agonist Potentiating NGF-Induced Neurite Outgrowth in PC12 Cells. Bioorganic Med. Chem..

[62] Motawe Z.Y., Abdelmaboud S.S., Cuevas J., Breslin J.W. (2020). PRE-084 as a Tool to Uncover Potential Therapeutic Applications for Selective Sigma-1 Receptor Activation. Int. J. Biochem. Cell. Biol..

[63] Medraño-Fernandez I., Bestetti S., Bertolotti M., Bienert G.P., Bottino C., Laforenza U., Rubartelli A., Sitia R. (2016). Stress Regulates Aquaporin-8 Permeability to Impact Cell Growth and Survival. Antioxid. Redox Signal..

[64] Pellavio G., Rui M., Caliogna L., Martino E., Gastaldi G., Collina S., Laforenza U. (2017). Regulation of Aquaporin Functional Properties Mediated by the Antioxidant Effects of Natural Compounds. Int. J. Mol. Sci..

[65] Pellavio G., Rossino G., Gastaldi G., Rossi D., Linciano P., Collina S., Laforenza U. (2021). Sigma-1 Receptor Agonists Acting on Aquaporin-Mediated H_2_O_2_ Permeability: New Tools for Counteracting Oxidative Stress. Int. J. Mol. Sci..

[66] Marra A., Rossi D., Pignataro L., Bigogno C., Canta A., Oggioni N., Malacrida A., Corbo M., Cavaletti G., Peviani M. (2016). Toward the Identification of Neuroprotective Agents: G-Scale Synthesis, Pharmacokinetic Evaluation and CNS Distribution of (R)-RC-33, a Promising Sigma1 Receptor Agonist. Future Med. Chem..

[67] Okuyama S., Nakazato A. (1996). NE-100: A Novel Sigma Receptor Antagonist. CNS Drug Rev..

[68] Lee K.H., Lee D.W., Kang B.C. (2020). The ‘R’ Principles in Laboratory Animal Experiments. Lab. Anim. Res..

[69] Gross D., Tolba R.H. (2015). Ethics in Animal-Based Research. Eur. Surg. Res..

[70] Daina A., Michielin O., Zoete V. (2017). SwissADME: A Free Web Tool to Evaluate Pharmacokinetics, Drug-Likeness and Medicinal Chemistry Friendliness of Small Molecules. Sci. Rep..

[71] Cassar S., Adatto I., Freeman J.L., Gamse J.T., Iturria I., Lawrence C., Muriana A., Peterson R.T., Van Cruchten S., Zon L.I. (2020). Use of Zebrafish in Drug Discovery Toxicology. Chem. Res. Toxicol..

[72] Hosszu A., Antal Z., Veres-Szekely A., Lenart L., Balogh D.B., Szkibinszkij E., Illesy L., Hodrea J., Banki N.F., Wagner L. (2018). The Role of Sigma-1 Receptor in Sex-Specific Heat Shock Response in an Experimental Rat Model of Renal Ischaemia/Reperfusion Injury. Transpl. Int..

[73] Harris C., Hansen J.M. (2012). Developmental Toxicology: Methods and Protocols.

[74] Brannen K.C., Panzica-Kelly J.M., Danberry T.L., Augustine-Rauch K.A. (2010). Development of a Zebrafish Embryo Teratogenicity Assay and Quantitative Prediction Model. Birth Defects Res. B Dev. Reprod Toxicol..

[75] Clarke S.E., Jeffrey P. (2001). Utility of Metabolic Stability Screening: Comparison of in Vitro and in Vivo Clearance. Xenobiotica.

[76] Brian Houston J. (1994). Utility of in Vitro Drug Metabolism Data in Predicting in Vivo Metabolic Clearance. Biochem. Pharmacol..

[77] Riley R.J., McGinnity D.F., Austin R.P. (2005). A Unified Model for Predicting Human Hepatic, Metabolic Clearance from in Vitro Intrinsic Clearance Data in Hepatocytes and Microsomes. Drug Metab. Dispos..

[78] Davies B., Morris T. (1993). Physiological Parameters in Laboratory Animals and Humans. Pharm. Res..

[79] Dubuisson D., Dennis S.G. (1977). The Formalin Test: A Quantitative Study of the Analgesic Effects of Morphine, Meperidine, and Brain Stem Stimulation in Rats and Cats. Pain.

[80] Ho Kim S., Mo Chung J. (1992). An Experimental Model for Peripheral Neuropathy Produced by Segmental Spinal Nerve Ligation in the Rat. Pain.

[81] Bonin R.P., Bories C., De Koninck Y. (2014). A Simplified Up-Down Method (SUDO) for Measuring Mechanical Nociception in Rodents Using von Frey Filaments. Mol. Pain.

