# Supplementary material for: Discovery of RC-752, a Novel Sigma-1 Receptor Antagonist with Antinociceptive Activity: A Promising Tool for Fighting Neuropathic Pain
Source: Pharmaceuticals (Basel). 2023 Jul 5;16(7):962. doi: 10.3390/ph16070962 (PMC10386076; doi:10.3390/ph16070962)

pharmaceuticals

Discovery of RC-752, a Novel Sigma-1 Receptor Antagonist with Antinociceptive Activity: A Promising Tool for Fighting Neuropathic Pain

Giacomo Rossino [1], Annamaria Marra [1], Roberta Listro [1], Marco Peviani [2], Elena Poggio [2], Daniela Curti [2], Giorgia Pellavio [3], Umberto Laforenza [3], Giulio Dondio [4], Dirk Schepmann [5], Bernhard Wünsch [5], Martina Bedeschi [6], Noemi Marino [6], Anna Tesei [6], Hee-Jin Ha [7], Young-Ho Kim [7], Jihyae Ann [8,9], Jeewoo Lee [8,9], Pasquale Linciano [1], Marcello Di Giacomo [1], Daniela Rossi [1,*] and Simona Collina [1]

1 Department of Drug Sciences, University of Pavia, 27100 Pavia, Italy; giacomo.rossino@unipv.it (G.R.);
annamariamarra86@gmail.com (A.M.); roberta.listro@unipv.it (R.L.); pasquale.linciano@unipv.it (P.L.);
marcello.digiacomo@unipv.it (M.D.G.); simona.collina@unipv.it (S.C.)
2 Department of Biology and Biotechnology "L. Spallanzani", University of Pavia, 27100 Pavia, Italy; marco.peviani@unipv.it (M.P.); elena.poggio01@universitadipavia.it (E.P.); daniela.curti@unipv.it (D.C.)
3 Human Physiology Unit, Department of Molecular Medicine, University of Pavia, 27100 Pavia, Italy;
giorgia.pellavio@unipv.it (G.P.); lumberto@unipv.it (U.L.)
4 Aphad SrL, Via della Resistenza, 65, 20090 Buccinasco, Italy; g.dondio@aphad.eu
5 Institut für Pharmazeutische und Medizinische Chemie, Westfälische Wilhelms-Universität Münster, Corrensstraße 48, D-48149 Münster, Germany; dirk.schepmann@uni-muenster.de (D.S.);
wuensch@uni-muenster.de (B.W.)
6 BioScience Laboratory, IRCCS Istituto Romagnolo per lo Studio dei Tumori (IRST) "Dino Amadori",
47014, Meldola, Italy; martina.bedeschi@irst.emr.it (M.B.); noemi.marino@irst.emr.it (N.M.); anna.tesei@irst.emr.it (A.T.)
7 Medifron DBT, Seoul 08502, Republic of Korea; hjha95@gmail.com (H.H.); youngho1963@gmail.com (Y.K.)
8 Laboratory of Medicinal Chemistry, College of Pharmacy, Seoul National University, Seoul 08826, Republic of Korea; jihuya@snu.ac.kr (J.A.); jeewoo@snu.ac.kr (J.L.)
9 JMackem Co. Ltd., Seoul 08826, Republic of Korea
* Correspondence: daniela.rossi@unipv.it

Table of Content

Table S1. Physicochemical properties for compounds **1c** and **1d** computed by SwissADME — Page 2
Table S2. Sample score sheet — Page 2
Figure S1. Cytotoxicity evaluation (expressed as % of cell survival) for compounds *(R/S)*-**1c** and *(R/S)*-**1d** against CHME-5 and MRC-5 cell lines. — Page 4
Figure S2. Kinetic stability profile of (R/S)-**1d** in mouse liver microsomes — Page 5
^{1}H-NMR spectra for compound **1a-3e** — Page 6
Representative UPLC-MS traces of compounds **1a, 1c,1d** and **2d**. — Page 14

Table S1. Physicochemical properties for compounds **1c** and **1d** computed by SwissADME

	**1c**	**1d**
**MW**	345.48 g/mol	309.45 g/mol
**N. Heavy Atoms**	26	23
**Fraction Csp3**	0.25	0.43
**N. Rotatable bonds**	8	6
**HBA**	2	2
**HBD**	1	1
**TPSA**	23.47	23.47
**cLogP**	4.63	4.07
**Water solubility**	Moderately soluble	Moderately soluble
**GI absorption**	High	High
**BBB Permeant**	Yes	Yes
**P-gp Substrate**	Yes	No
**Lipinski's RO5**	Yes	Yes

Table S2. Sample score sheet

The sample score sheet was edited on the basis of already existing sheets (Book of Developmental Toxicology,2012, Harris C. et al. and development of a zebrafish embryo teratogenicity assay and quantitative prediction model, 2010, Brannen KC et al.). Tail, heart, face, swim bladder, pigmentation, yolk and abdomen edema were taken into consideration as fundamental parameters to evaluate embryos development's impairment. Scores were determined comparing treated and untreated larvae (control set). According to Harris and Hansen a score of 5-0.5 is assigned to each structure. A score of 5 denotes a normal morphology, a score of 4 denotes a slight anomaly or late development, a sore of 3 indicates mild deformations, a score of 2 indicates a moderate malformation, a score of 1 denotes a severe malformation and a score of 0.5 is assigned when the structure is completely absent. The "X" is used to identify the specific malformation and the relative concentration of treatment.

PL16	[μM]	CTRL	0,1	1	10	50
Treatment group						
Date	25/05/2022					
Operator	MB/NM					
viable (normalized on total)		7 (10)	8 (8)	7 (8)	4 (8)	0 (8)
Body shape abnormal					X	
Tail score		5	5	4	1	0,5
Tail-Kinked					x	
Tail-Bent/curved				x	X	
Tail-Short					x	
Heart score		5	5	3	1	0,5
Slow heartbeat					x	
No heartbeat						
Heart—Small						
Heart—Enlarged				x	X	
Face score		5	5	5	1	0,5
Optic vesicle(s) small					x	
Swim bladder—Not well inflated/not evident					x	
Poor pigmentation					X	
Excess pigmentation						
Yolk ball—Remnant excessive					X	
Yolk ball—Remnant, dark						
Abdomen distended or edema					X	

PL24	[µM]	CTRL	0,1	1	10	50
Treatment group						
Date	25/05/2022					
Operator	MB/NM					
viable (normalized on total)		7 (10)	7 (8)	4 (8)	8 (8)	0 (8)
Body shape abnormal						x
Tail score		5	5	5	4	0,5
Tail-Kinked					x	
Tail-Bent/curved						
Tail-Short						
Heart score		5	5	5	3	0,5
Slow heartbeat						
No heartbeat						
Heart—Small						
Heart—Enlarged					x	
Face score		5	5	5	5	0,5
Optic vesicle(s) small						
Swim bladder—Not well inflated/not evident					x	
Poor pigmentation						
Excess pigmentation						
Yolk ball—Remnant excessive						
Yolk ball—Remnant, dark						
Abdomen distended or edema						

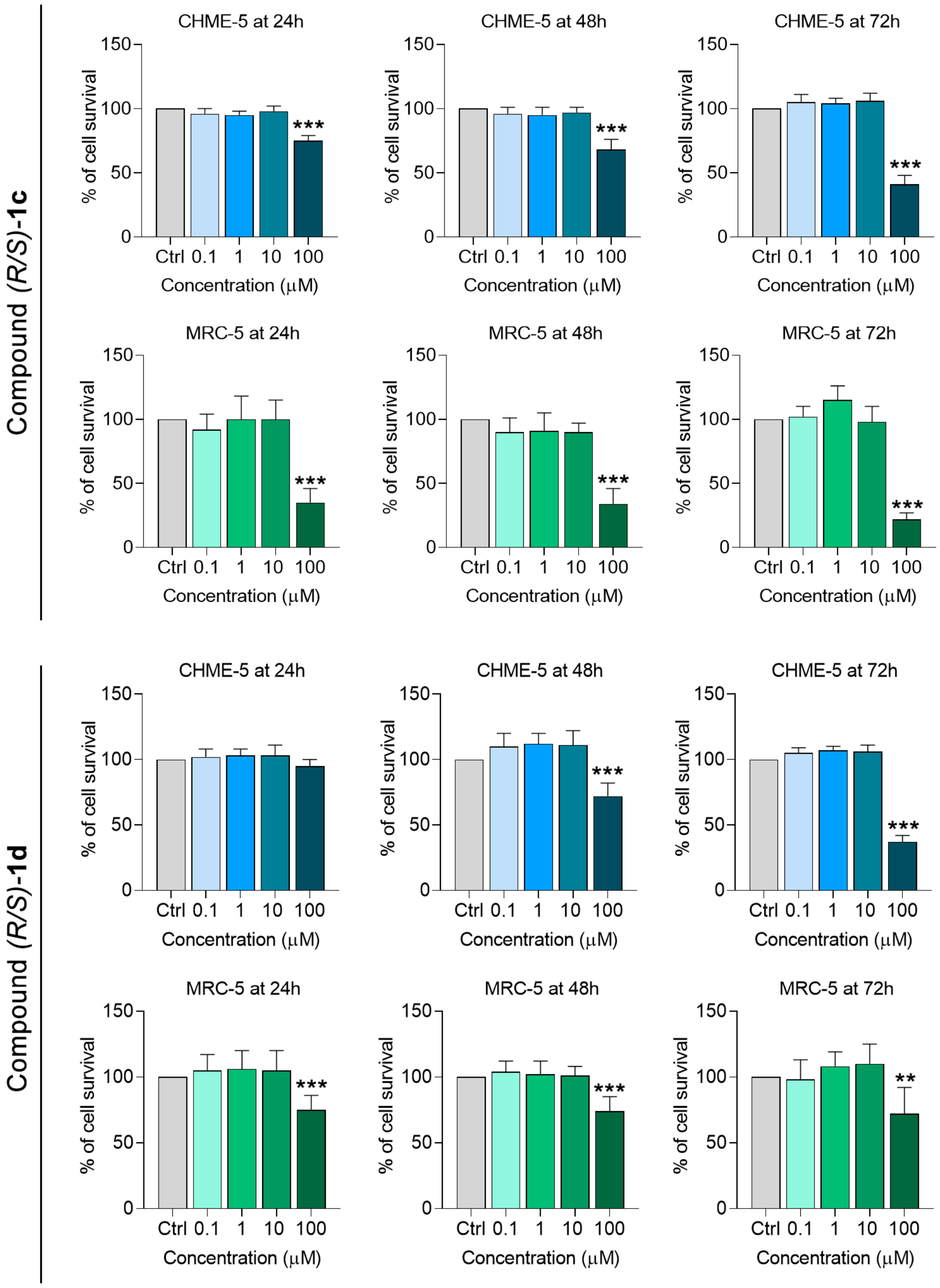

Figure S1. Cytotoxicity evaluation (expressed as % of cell survival) for compounds *(R/S)*-**1c** and *(R/S)*-**1d** against CHME-5 and MRC-5 cell lines. Cell survival was evaluated at 0.1, 1, 10, 100 μM at 24, 48, 72 h after exposure. * $p< 0.05$; ** $P<0.01$; *** $p<0.0001$.

Figure S2. Kinetic stability profile of (R/S)-**1d** in mouse liver microsomes.

^{1}H-NMR spectra for compound **1a**

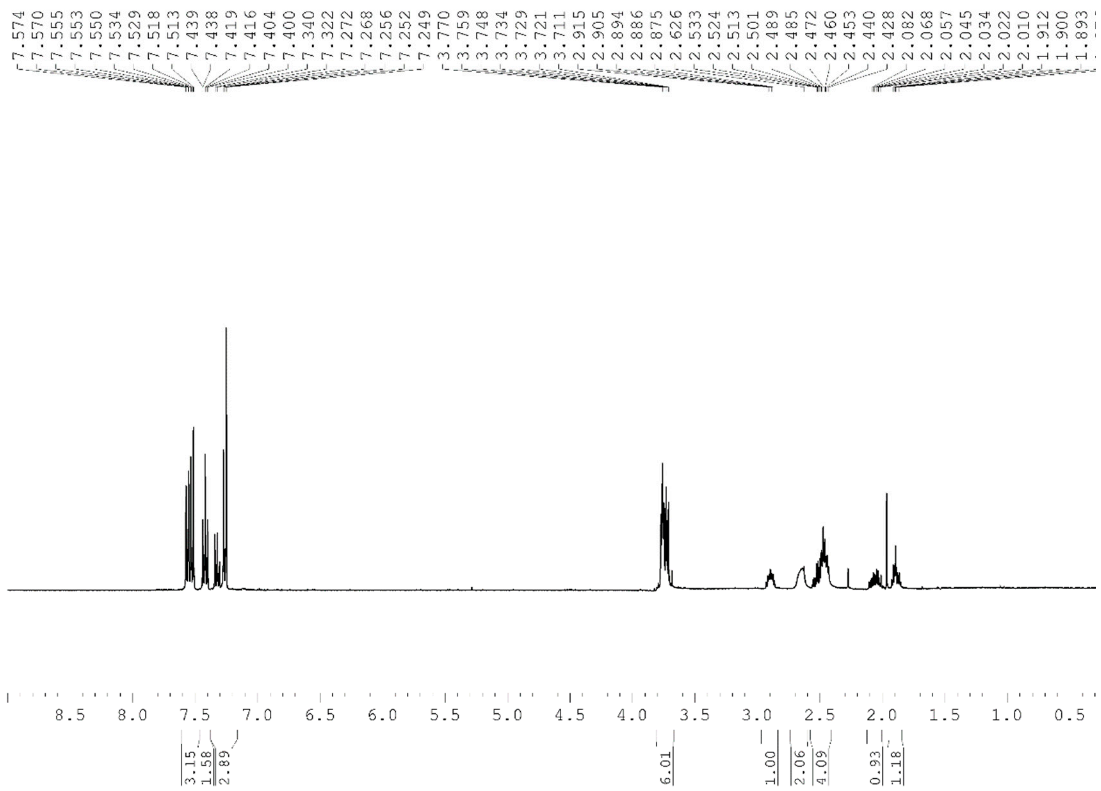

^{1}H-NMR spectra for compound **1b**

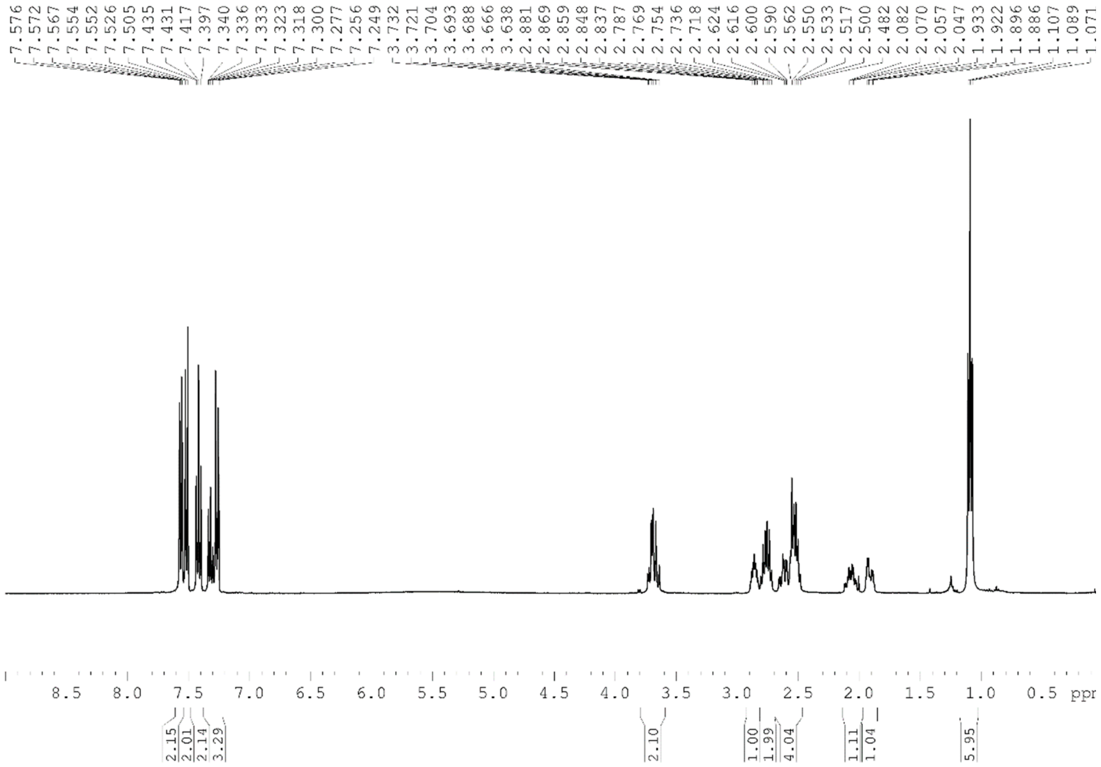

^{1}H-NMR spectra for compound **1c**

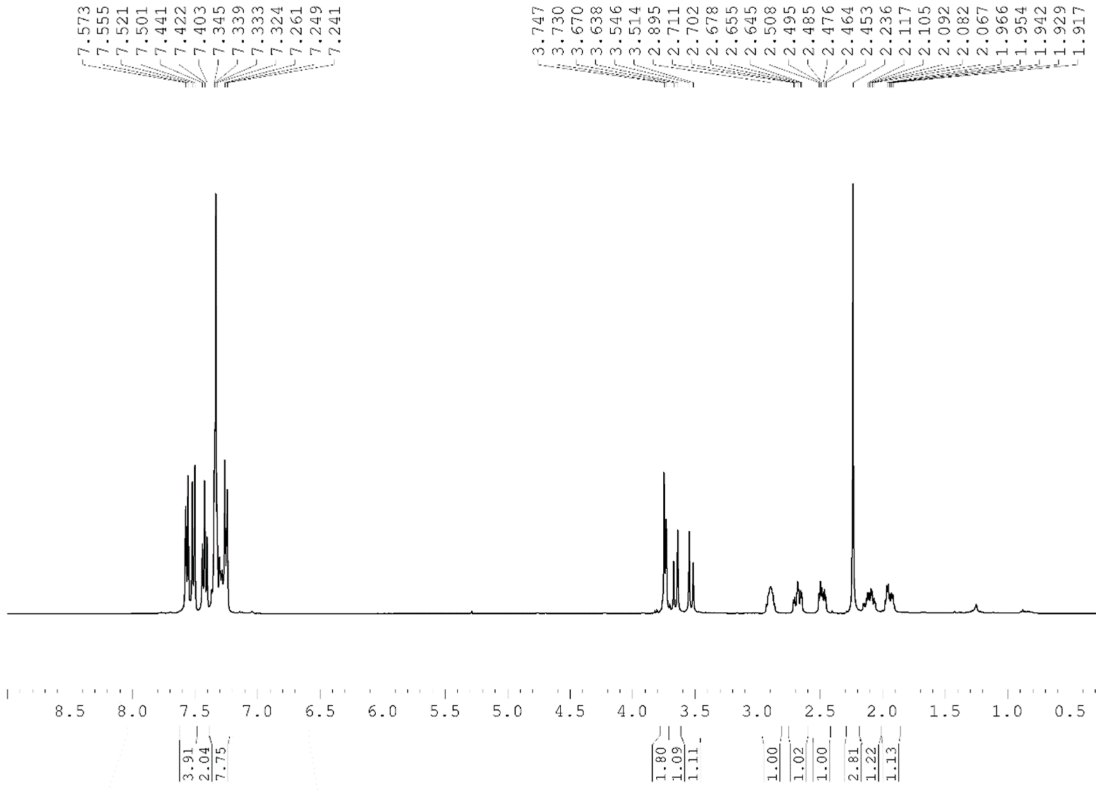

^{1}H-NMR spectra for compound **1d**

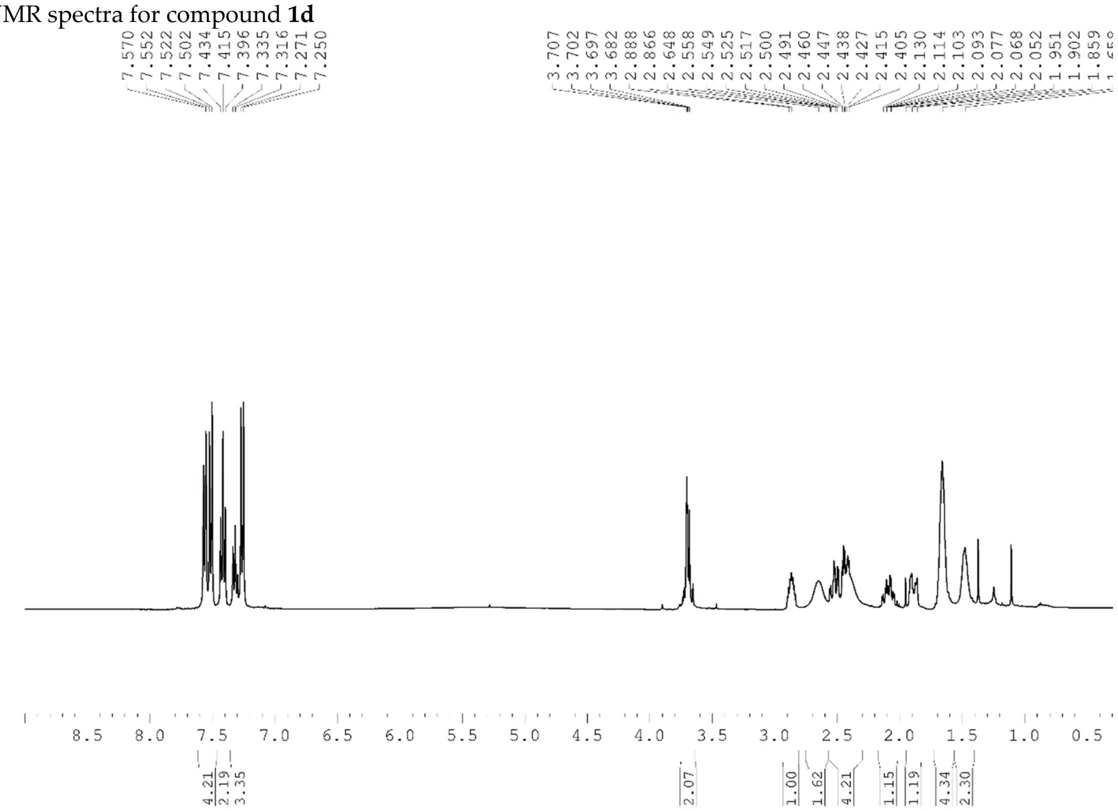

^{1}H-NMR spectra for compound **1e**

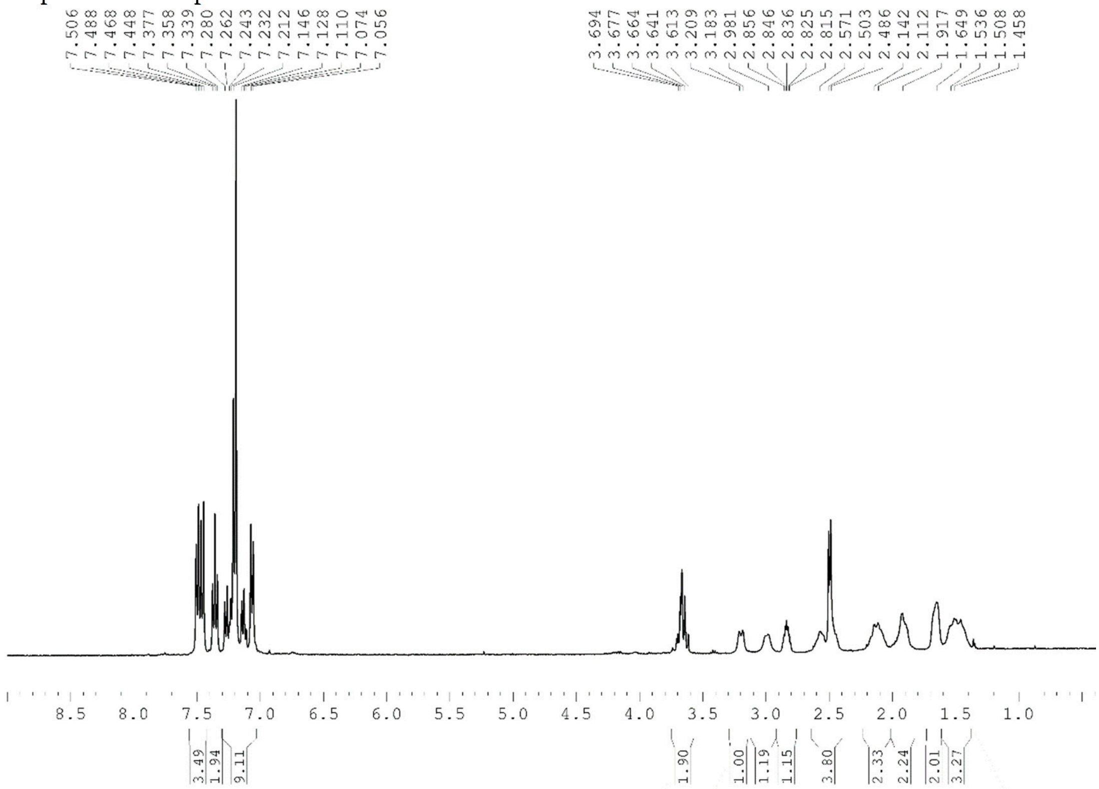

^{1}H-NMR spectra for compound **2a**

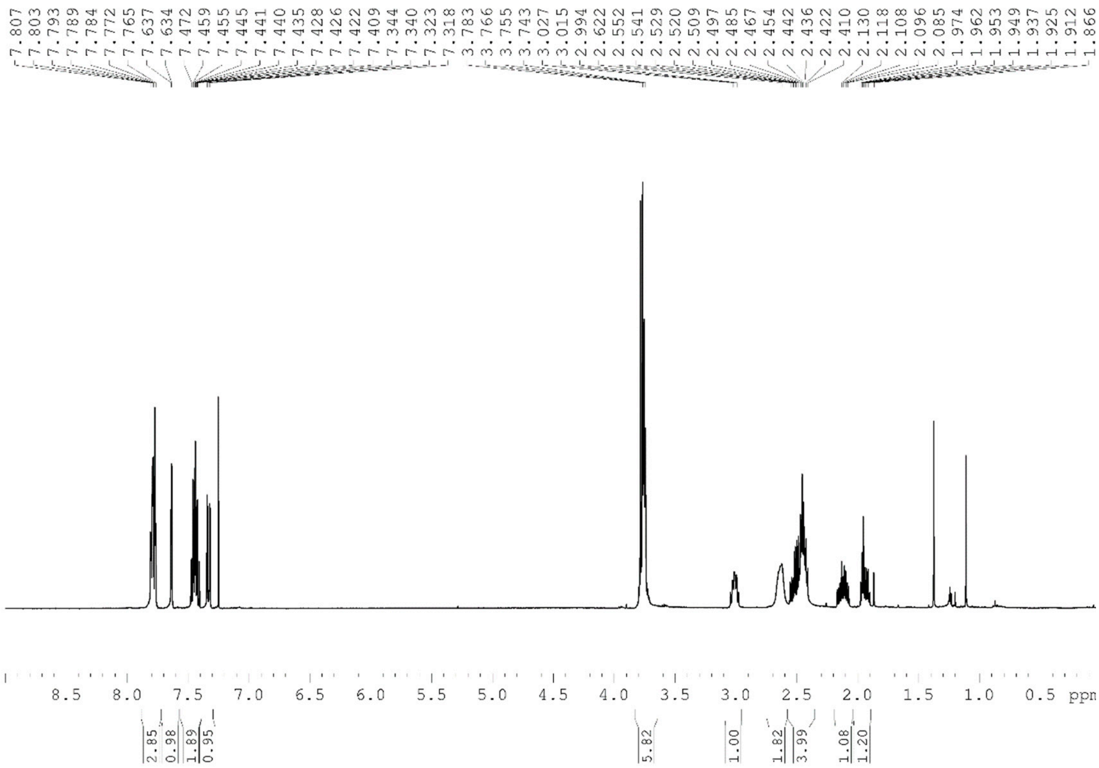

^{1}H-NMR spectra for compound **2b**

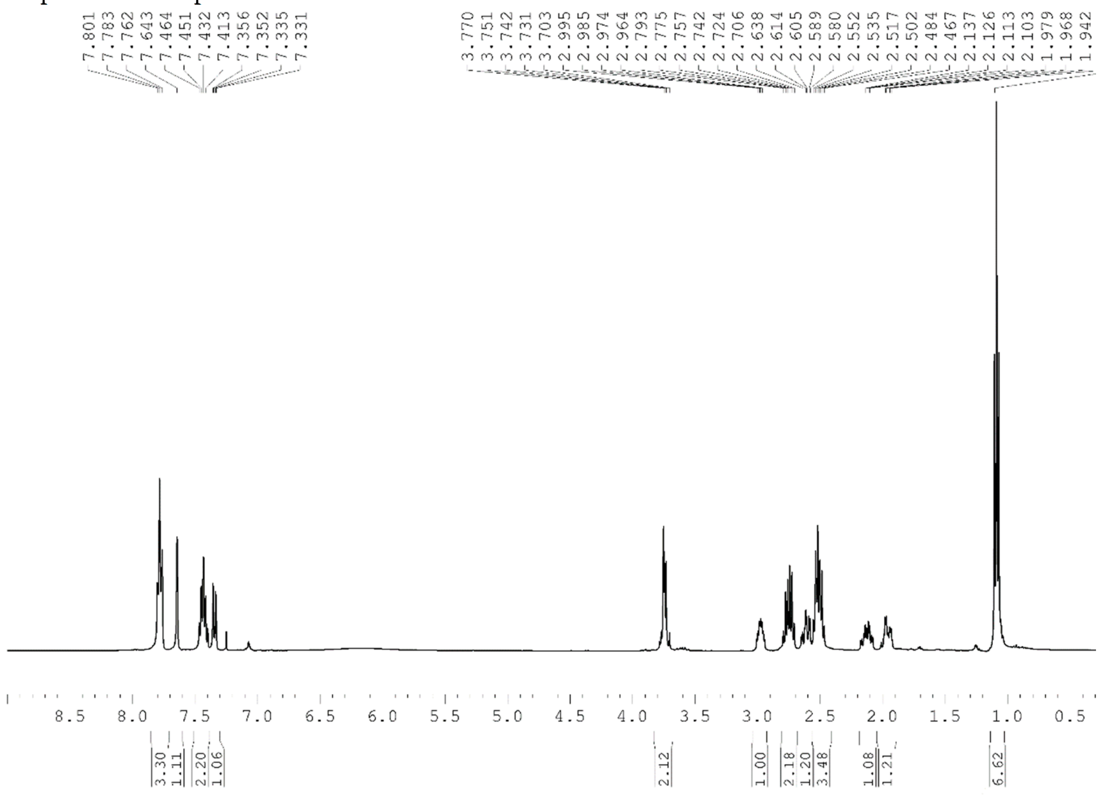

^{1}H-NMR spectra for compound **2c**

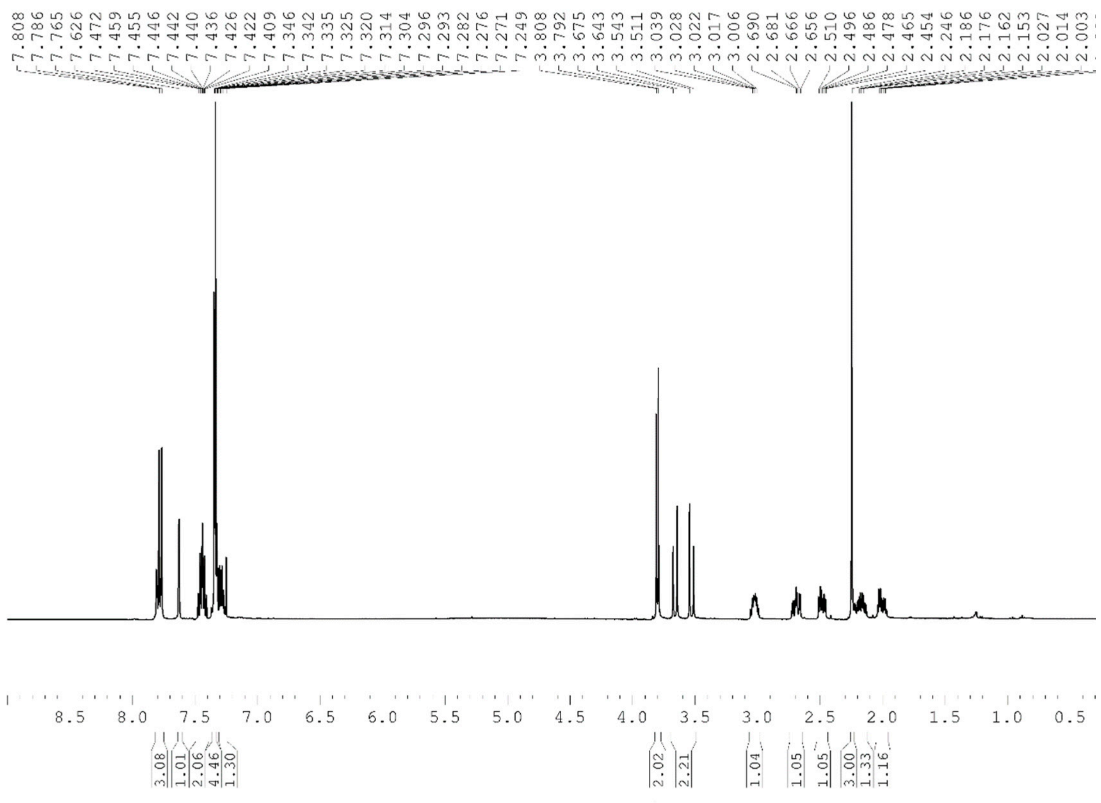

^{1}H-NMR spectra for compound **2d**

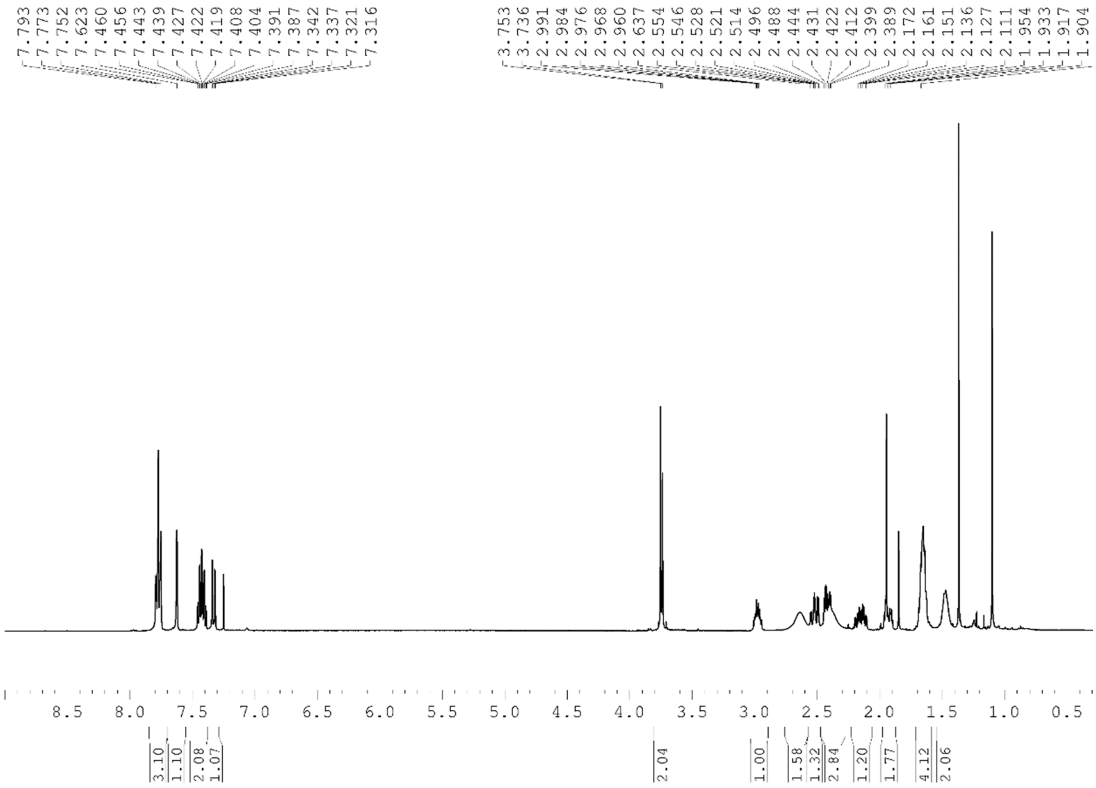

^{1}H-NMR spectra for compound **2e**

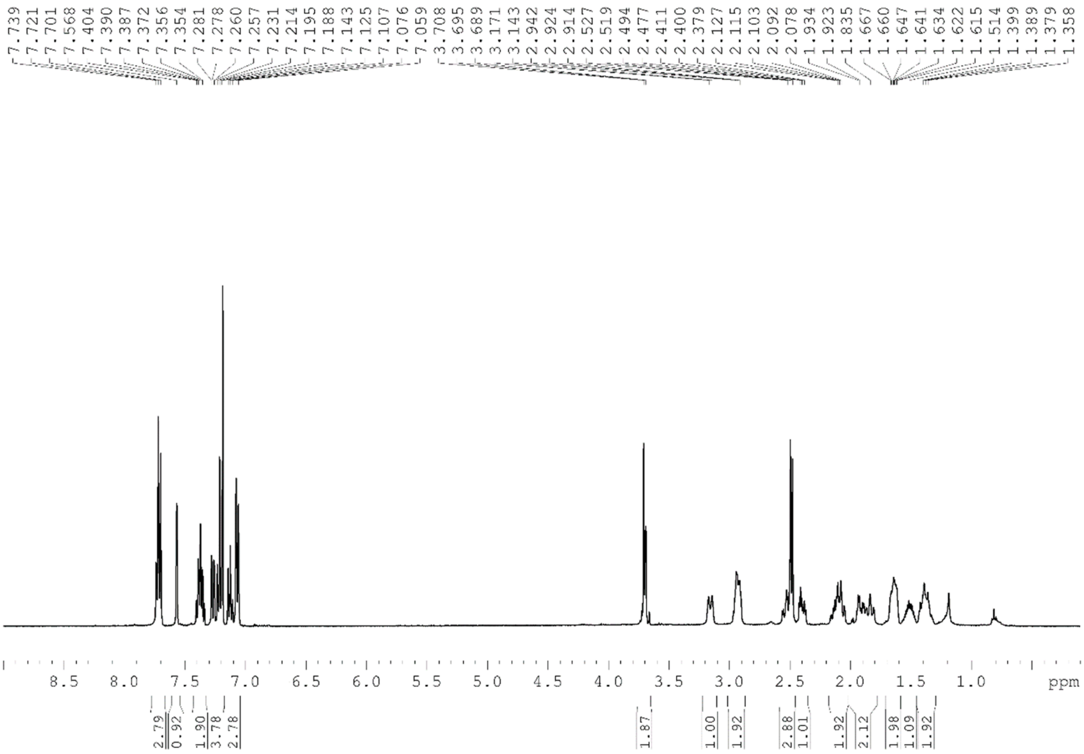

^{1}H-NMR spectra for compound **3a**

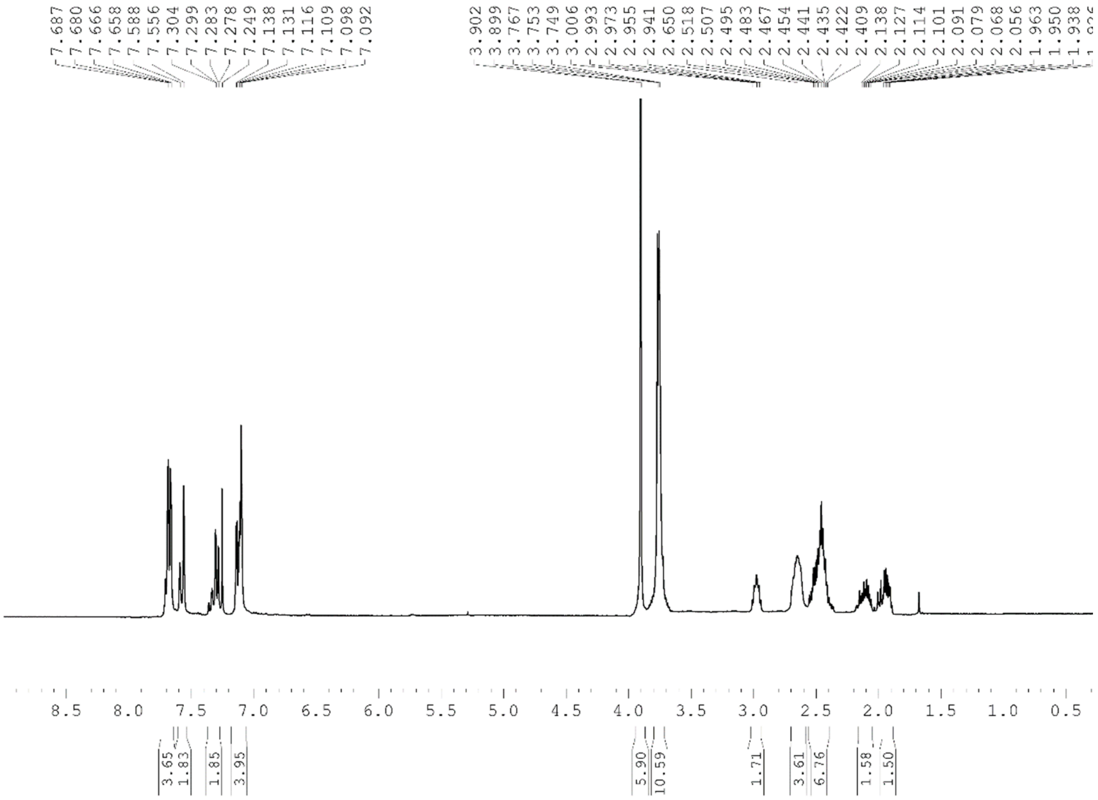

^{1}H-NMR spectra for compound **3b**

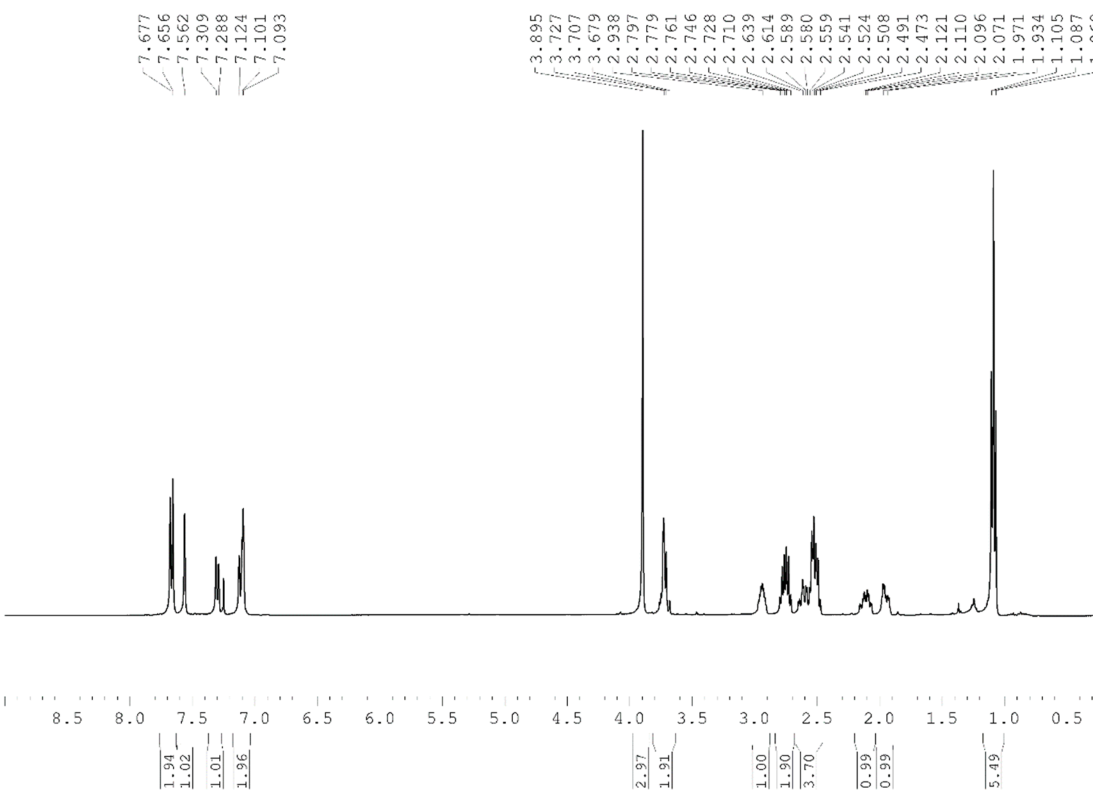

^{1}H-NMR spectra for compound **3c**

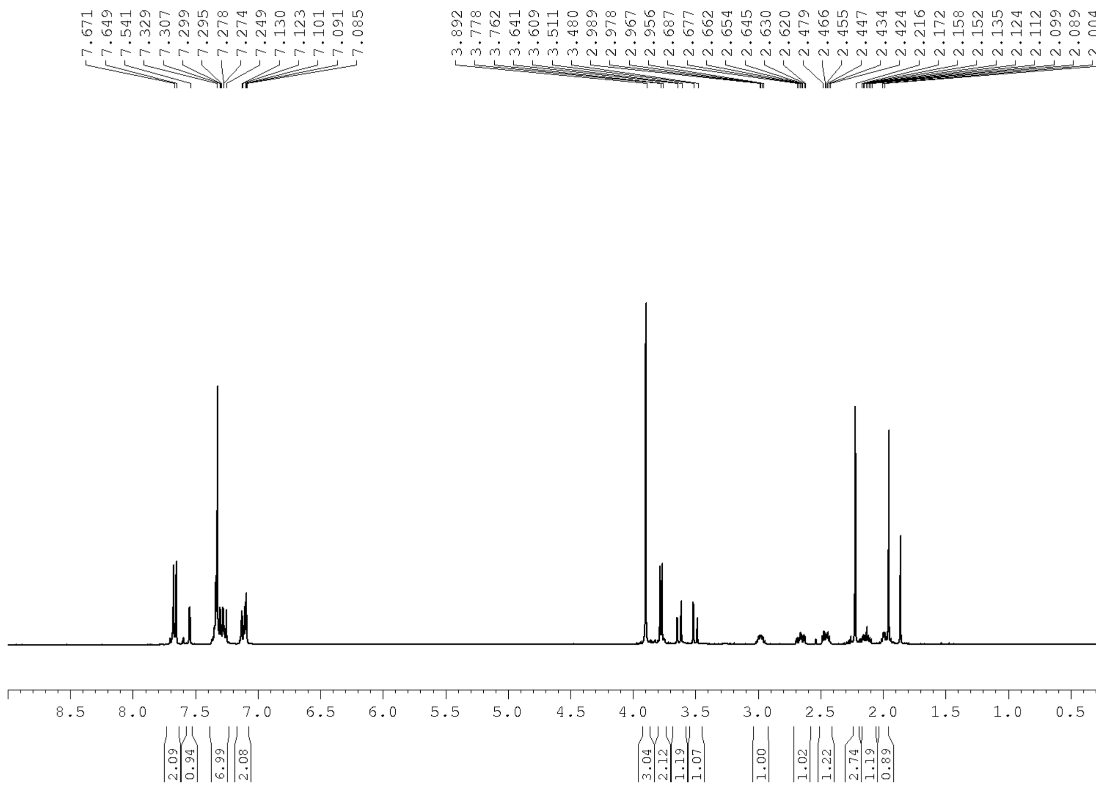

^{1}H-NMR spectra for compound **3d**

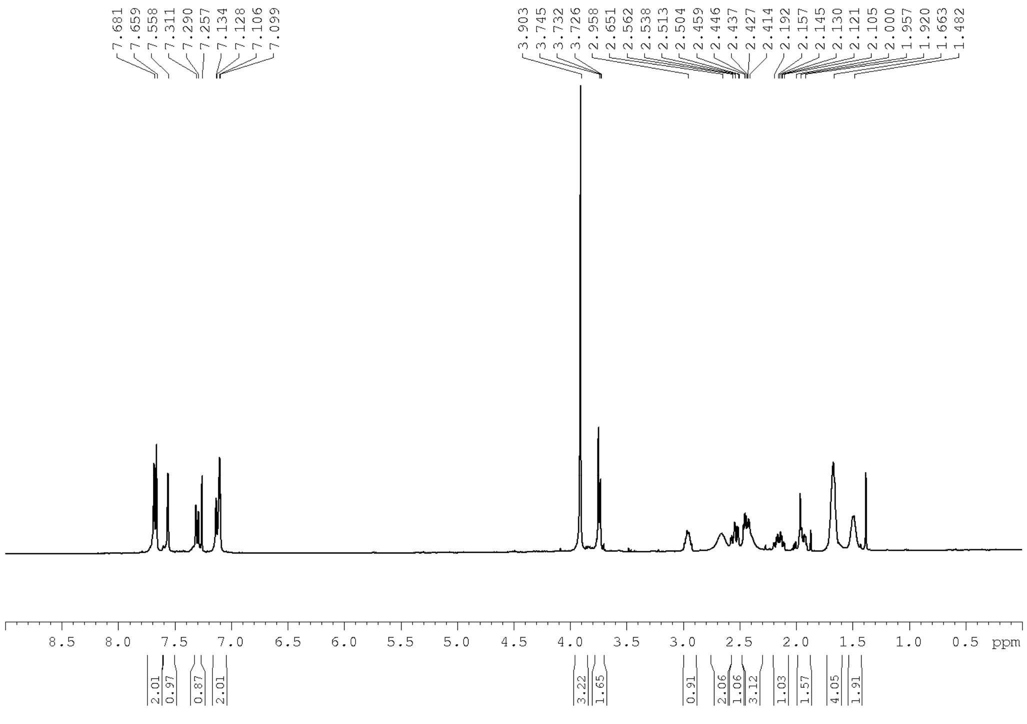

^{1}H-NMR spectra for compound **3e**

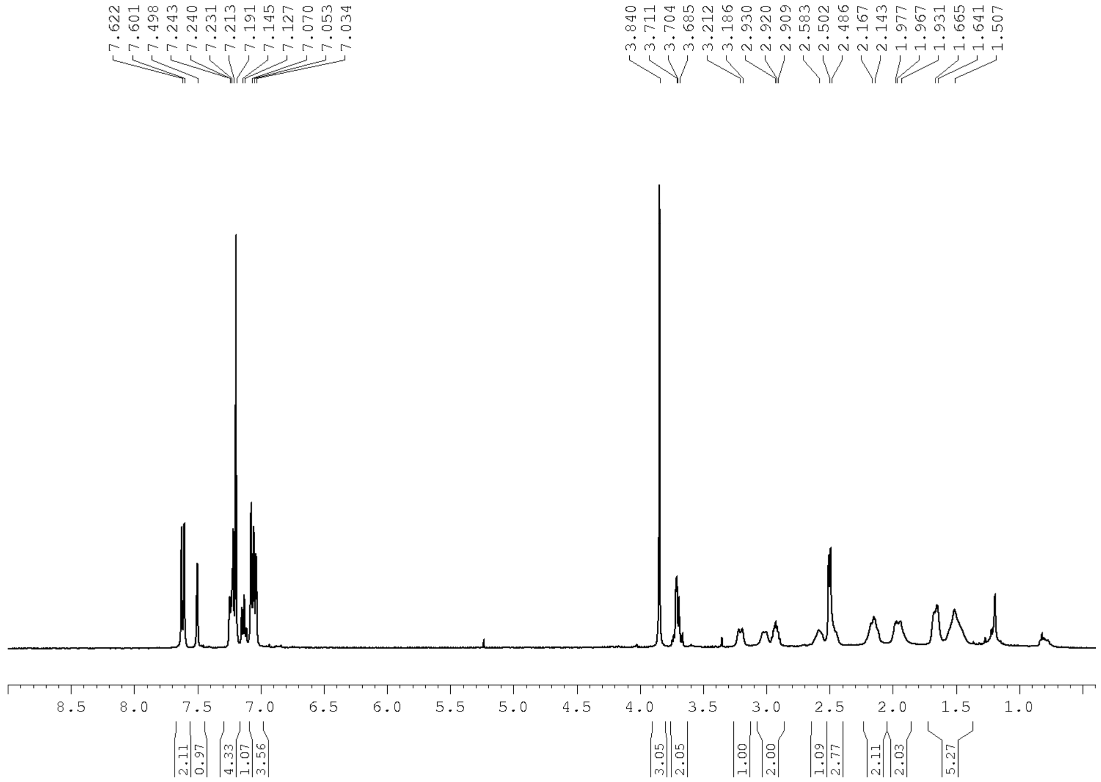

Representative UPLC traces of compounds tested in vivo and in vitro.
Compound **1a**
PV1582 MW311.19

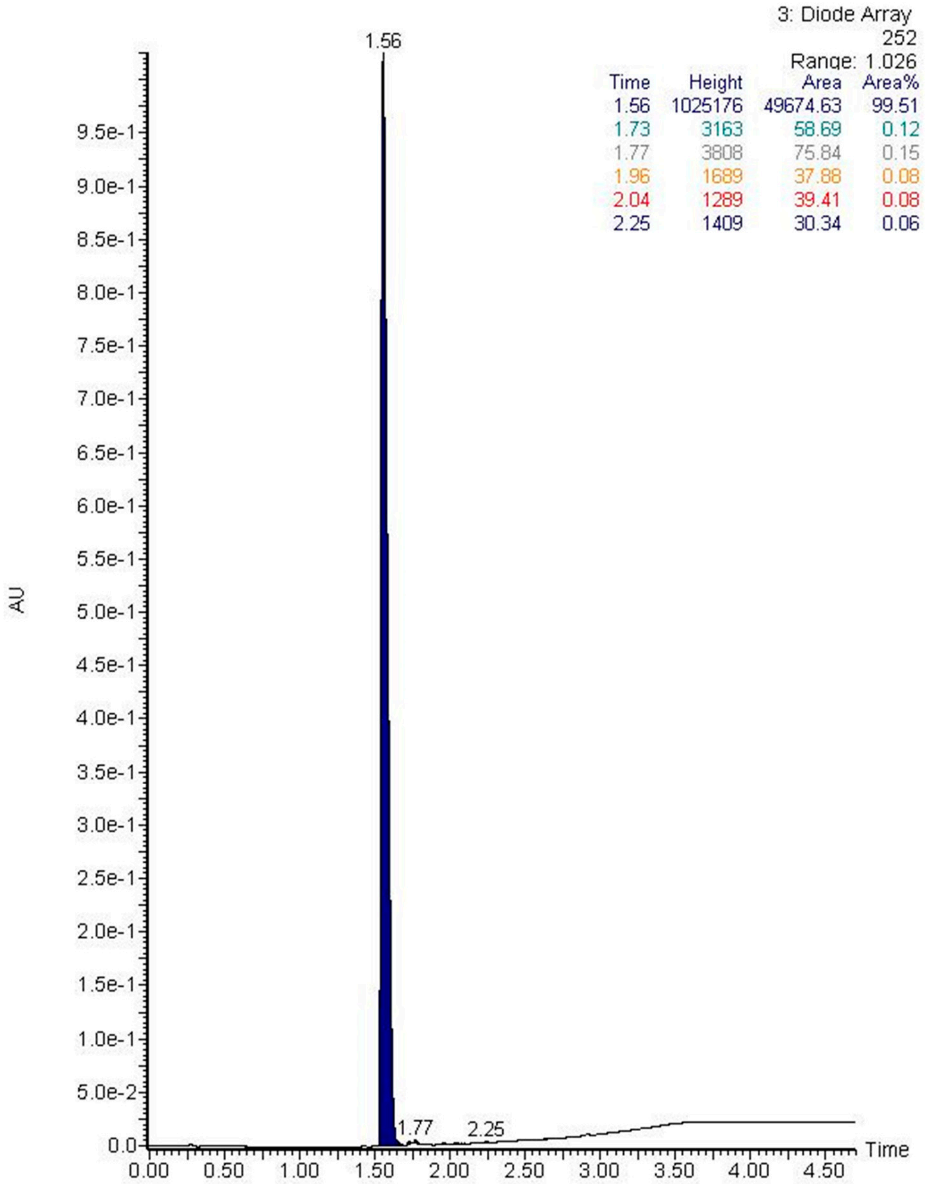

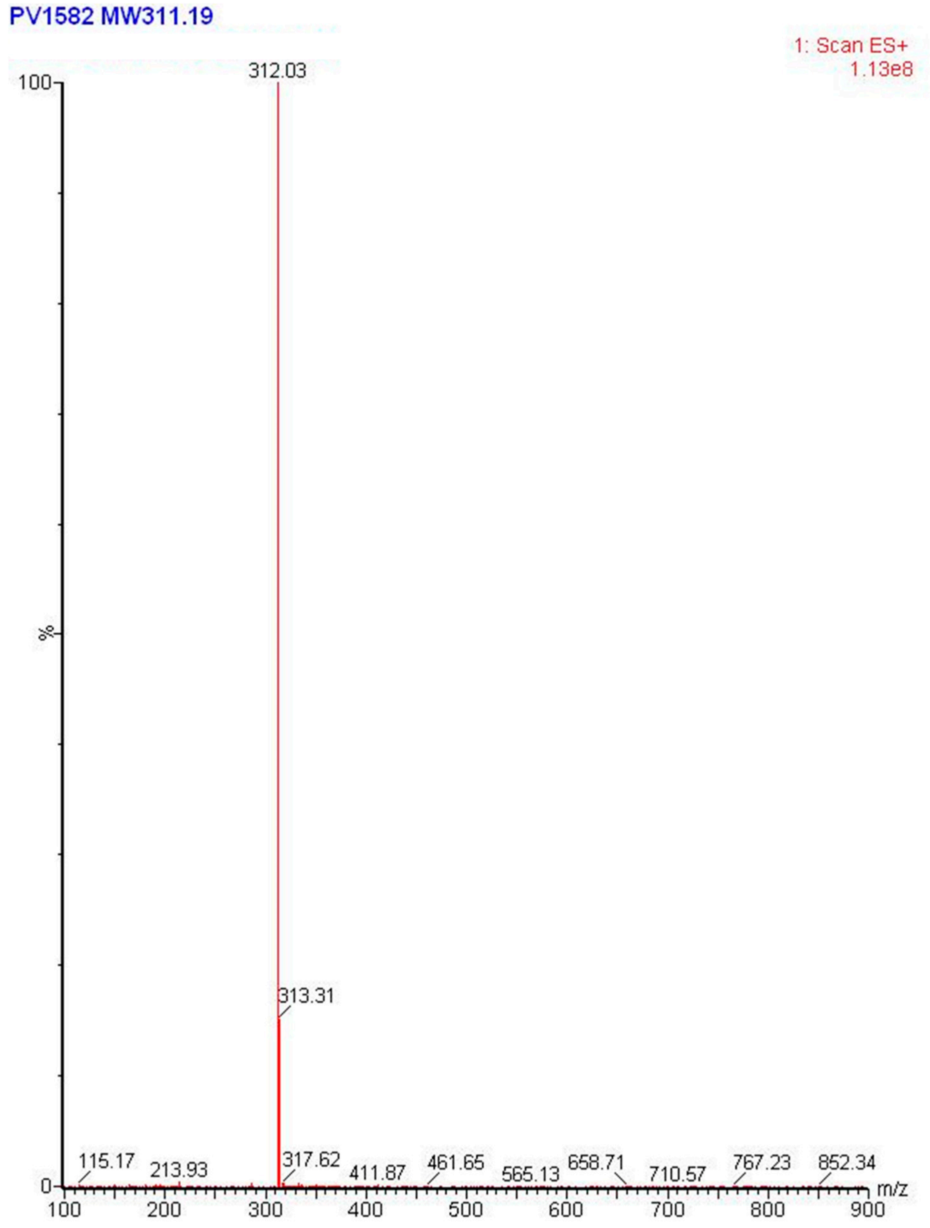
PV1582 MW311.19
1: Scan ES+
1.13e8
312.03
313.31
115.17
213.93
317.62
411.87
461.65
565.13
658.71
710.57
767.23
852.34
100
%
0
100
200
300
400
500
600
700
800
900
m/z

Compound **1c**

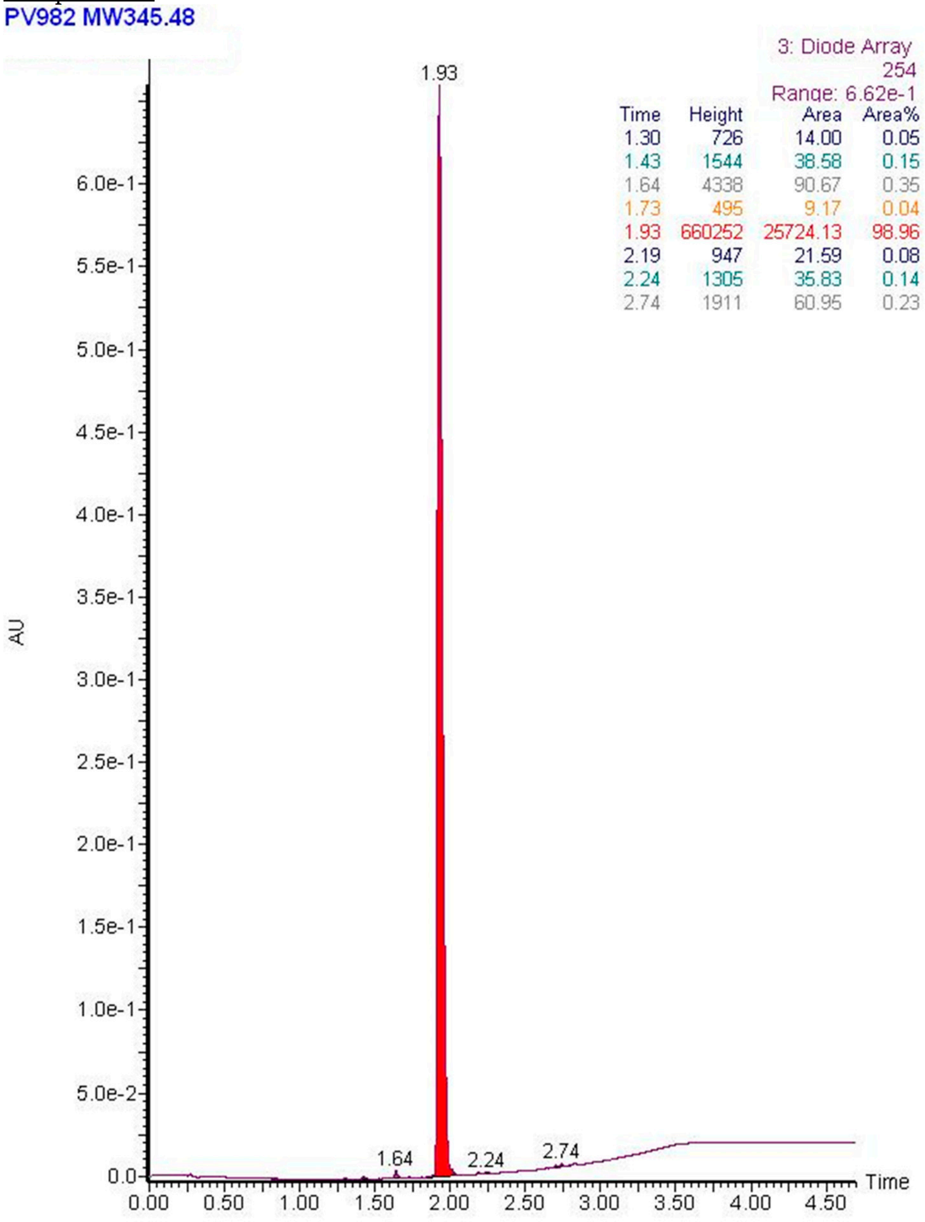
PV982 MW345.48
3: Diode Array
254
Range: 6.62e-1
Time Height Area Area%
1.30 726 14.00 0.05
1.43 1544 38.58 0.15
1.64 4338 90.67 0.35
1.73 495 9.17 0.04
1.93 660252 25724.13 98.96
2.19 947 21.59 0.08
2.24 1305 35.83 0.14
2.74 1911 60.95 0.23
1.93
1.64
2.24
2.74
AU
Time

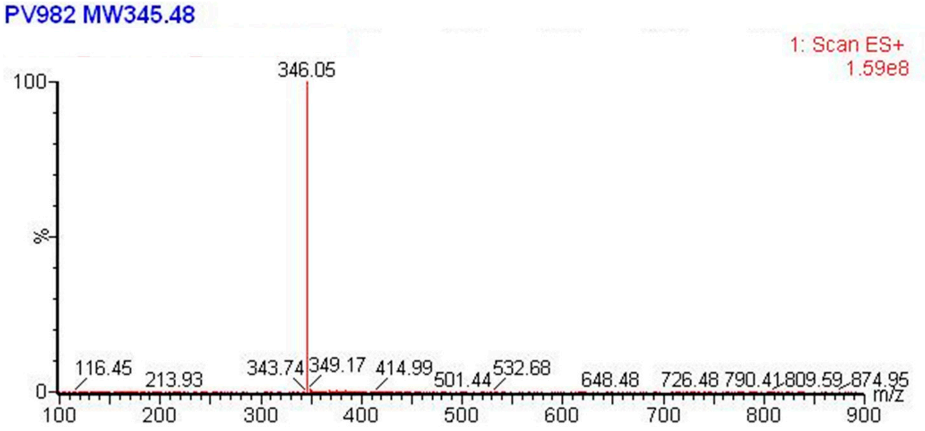
PV982 MW345.48
1: Scan ES+
1.59e8
346.05
116.45
213.93
343.74
349.17
414.99
501.44
532.68
648.48
726.48
790.41
809.59
874.95
%
m/z

Compound **1d**

PV752 MW309.45

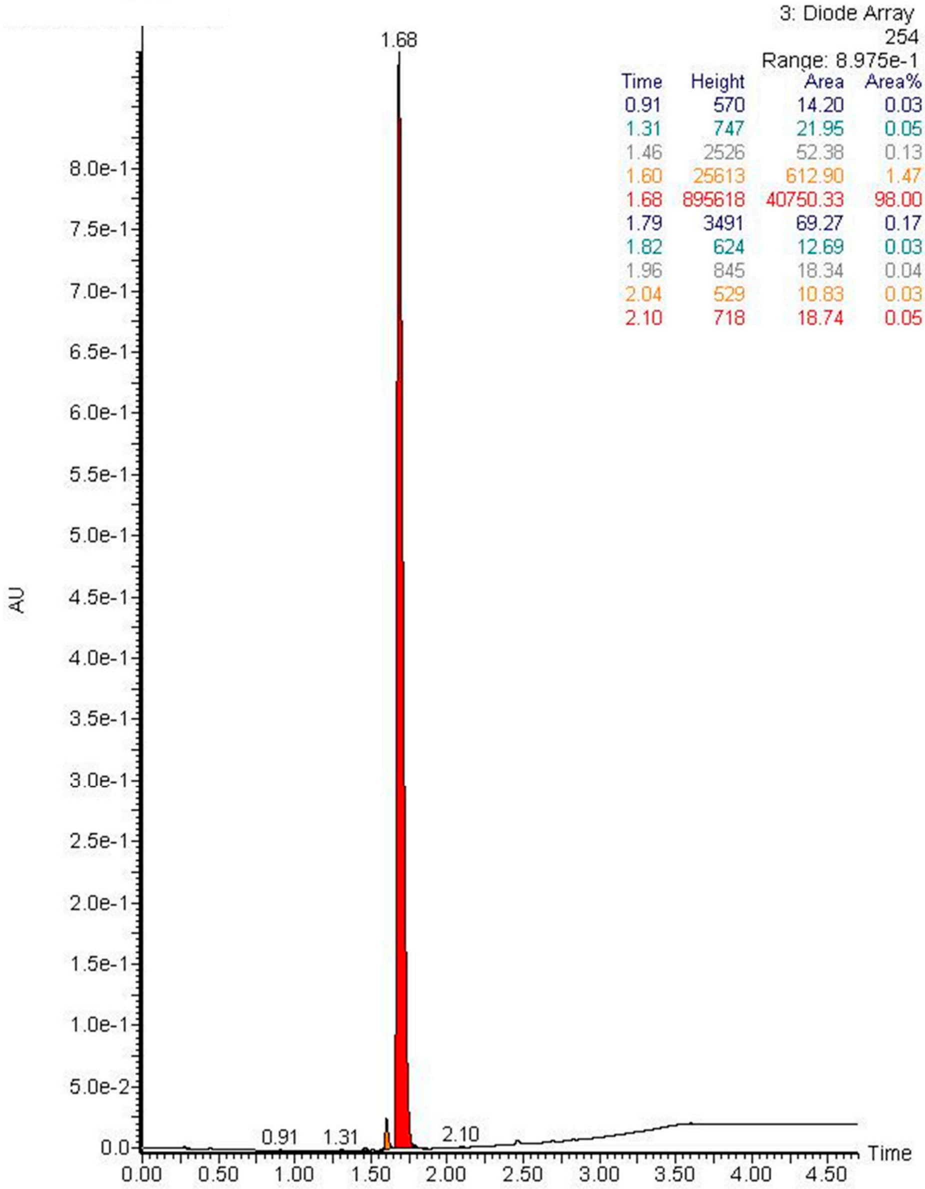

PV752 MW309.45

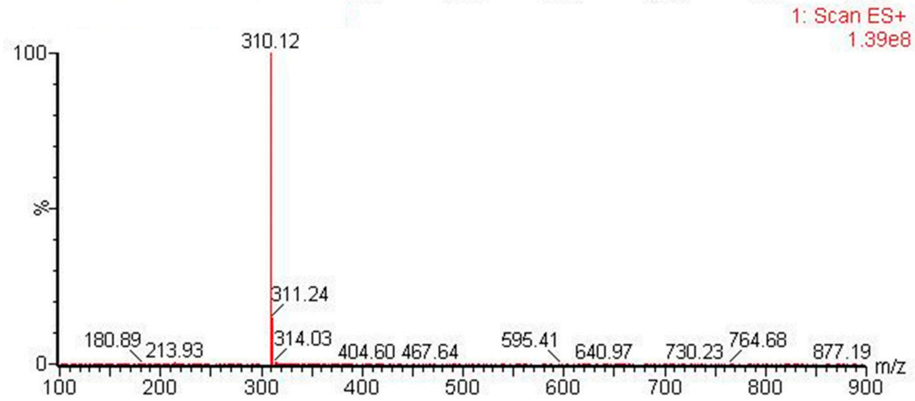

<u>Compound **2d**</u>

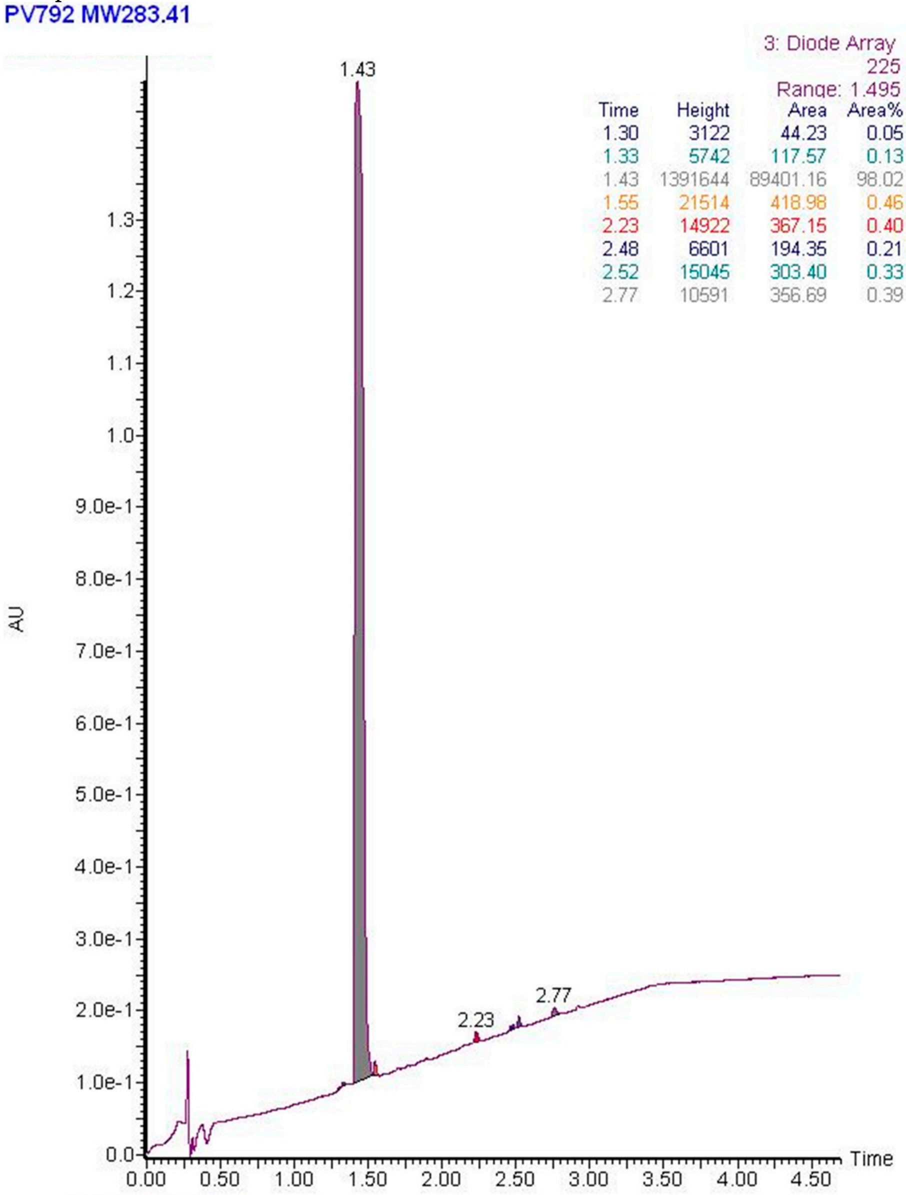

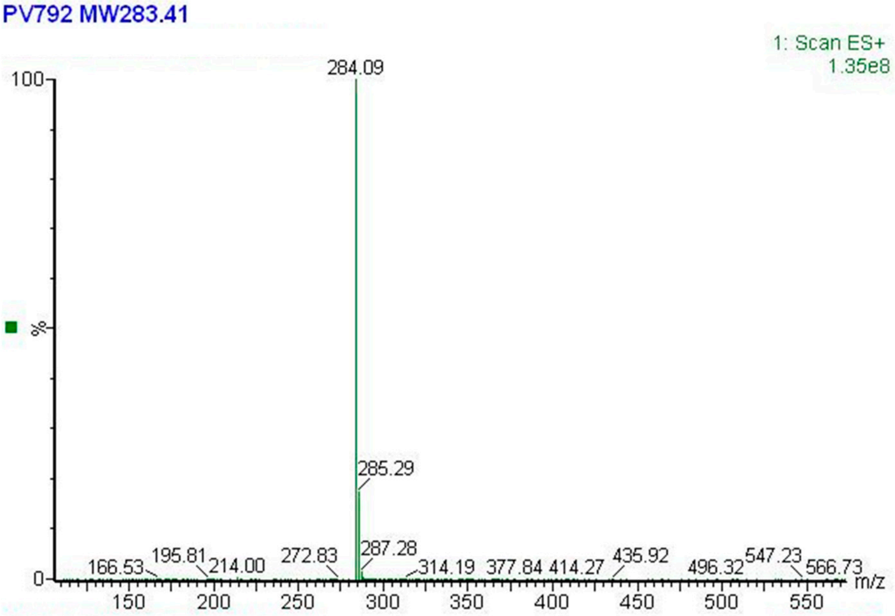

Supplement: Supplementary file 1 [file pharmaceuticals-16-00962-s001.zip › pharmaceuticals-2452069-supplementary.pdf]
